# Challenging Cognitive Load Theory: The Role of Educational Neuroscience and Artificial Intelligence in Redefining Learning Efficacy

**DOI:** 10.3390/brainsci15020203

**Published:** 2025-02-15

**Authors:** Evgenia Gkintoni, Hera Antonopoulou, Andrew Sortwell, Constantinos Halkiopoulos

**Affiliations:** 1 Department of Educational Sciences and Social Work, University of Patras, 26504 Patras, Greece; 2 Department of Management Science and Technology, University of Patras, 26334 Patras, Greece; hera@upatras.gr (H.A.); halkion@upatras.gr (C.H.); 3 School of Education, The University of Notre-Dame Australia, Sydney, NSW 2007, Australia; andrew.sortwell@nd.edu.au; 4 School of Health Sciences and Physiotherapy, University of Notre Dame Australia, 32 Mouat St, Fremantle, WA 6160, Australia; 5 Research Centre in Sports, Health and Human Development, University of Beira Interior, 6201-001 Covilhã, Portugal

**Keywords:** cognitive load theory, educational neuroscience, artificial intelligence, machine learning, personalized learning, adaptive learning, K-12 education, learning efficacy

## Abstract

*Background/Objectives:* This systematic review integrates Cognitive Load Theory (CLT), Educational Neuroscience (EdNeuro), Artificial Intelligence (AI), and Machine Learning (ML) to examine their combined impact on optimizing learning environments. It explores how AI-driven adaptive learning systems, informed by neurophysiological insights, enhance personalized education for K-12 students and adult learners. This study emphasizes the role of Electroencephalography (EEG), Functional Near-Infrared Spectroscopy (fNIRS), and other neurophysiological tools in assessing cognitive states and guiding AI-powered interventions to refine instructional strategies dynamically. *Methods:* This study reviews *n* = 103 papers related to the integration of principles of CLT with AI and ML in educational settings. It evaluates the progress made in neuroadaptive learning technologies, especially the real-time management of cognitive load, personalized feedback systems, and the multimodal applications of AI. Besides that, this research examines key hurdles such as data privacy, ethical concerns, algorithmic bias, and scalability issues while pinpointing best practices for robust and effective implementation. *Results:* The results show that AI and ML significantly improve Learning Efficacy due to managing cognitive load automatically, providing personalized instruction, and adapting learning pathways dynamically based on real-time neurophysiological data. Deep Learning models such as Convolutional Neural Networks (CNNs), Recurrent Neural Networks (RNNs), and Support Vector Machines (SVMs) improve classification accuracy, making AI-powered adaptive learning systems more efficient and scalable. Multimodal approaches enhance system robustness by mitigating signal variability and noise-related limitations by combining EEG with fMRI, Electrocardiography (ECG), and Galvanic Skin Response (GSR). Despite these advances, practical implementation challenges remain, including ethical considerations, data security risks, and accessibility disparities across learner demographics. *Conclusions:* AI and ML are epitomes of redefinition potentials that solid ethical frameworks, inclusive design, and scalable methodologies must inform. Future studies will be necessary for refining pre-processing techniques, expanding the variety of datasets, and advancing multimodal neuroadaptive learning for developing high-accuracy, affordable, and ethically responsible AI-driven educational systems. The future of AI-enhanced education should be inclusive, equitable, and effective across various learning populations that would surmount technological limitations and ethical dilemmas.

## 1. Introduction

Understanding how we learn has grown increasingly important to research and practice, mainly when evidence shows that Artificial Intelligence (AI) and Machine Learning (ML) have been widely explored for educational applications [1,2,3]. These technologies, combined with developments in cognitive science regarding the aspects related to memory retention and instructional strategies [4,5,6], reshape education methodologies and require an informed pedagogical framework from educators themselves [7,8,9]. AI and ML have great educational potential; effectively applying these techniques can improve cognitive processes, optimize instructional design, and develop adaptive learning environments. However, many researchers have empirically investigated their impact on cognitive load and Learning Efficacy (LE) [10,11,12].

Cognitive Load Theory (CLT) was first conceptualized to address limitations in the capability of working memory for educational studies [13,14,15]. The theory provides a fundamental theoretical framework for how instructional designs affect learning. It postulates that human working memory has limited capacity. For effective learning, the instructional strategy should be implemented in such a way as to minimize extraneous cognitive load and emphasize Germane Cognitive Load. Studies have shown improved learner retention because of optimized instructional materials [16,17]. Various studies show that AI-driven adaptive learning systems can optimize cognitive load management by automatically adjusting instructional materials, scaffolding complex concepts, and providing immediate feedback [18,19]. Research findings from integrating AI-based interventions within CLT indicate a considerable improvement in students’ engagement and reduced cognitive overload, leading to overall improvements in learning outcomes [20,21].

Educational Neuroscience (EdNeuro) has further contributed to how cognitive processes interact with instructional design. The application of neurophysiological tools like EEG and fNIRS, which are increasingly used to assess in real time the degree of engagement of learners’ cognitive processes [22,23,24,25], gave significant insight into how students process and retain information. Moreover, Big Data techniques that can adapt learning pathways dynamically according to student interactions enable AI-driven learning analytics [26,27,28,29], which allow the monitoring of cognitive states in real time for personalized interventions catering to the specific needs of learning. Moreover, these approaches increase cognitive efficiency and foster Self-Regulated Learning strategies [30,31,32,33].

The present systematic review of 103 empirical studies indicates that AI-based interventions significantly enhance student performance and knowledge retention. The transformative potential of AI and ML in education is underlined. Among others, these have highlighted the effectiveness of AI-driven tools in customizing instruction and adaptive feedback, thus managing cognitive load across diverse learning contexts, ranging from K-12 education to professional training. More importantly, research findings have highlighted the ethical concerns that arise with the integration of AI, such as data privacy, algorithmic bias, and equity of access to educational resources. Unless these are addressed, AI use in learning environments cannot be implemented responsibly.

This study contributes to the ongoing discourse at the intersection of CLT, EdNeuro, and AI in educational contexts. Synthesizing findings from a broad spectrum of empirical studies aims to provide evidence-based insights into optimizing cognitive processes and fostering personalized learning environments. Furthermore, it delineates future research directions, emphasizing the development of scalable AI-driven interventions that enhance lifelong learning and promote educational equity in an increasingly digitized world.

## 2. Literature Review

### 2.1. Cognitive Load Theory (CLT)

CLT defines learning as the process by which information is selected, organized, and integrated into memory, a process controlled by limitations in working memory [34,35]. The theory, introduced by Sweller [34], focuses on how effective instructional design should optimize cognitive resources to avoid overload and promote more efficient learning [36]. This is especially important when learning is complex and too high a cognitive load will negatively affect knowledge retention and transfer.

CLT distinguishes between three types of cognitive load: intrinsic, extraneous, and germane [37]. Intrinsic Cognitive Load (ICL) is determined by the nature of the material to be learned and the individual learner’s prior knowledge [38]. Highly structured content, such as algebraic manipulations in mathematics, requires learners to process multiple interrelated components, thereby increasing ICL [39]. To mitigate this, instructional strategies like segmenting and scaffolding progressively introduce complexity, allowing learners to integrate new information without overwhelming their cognitive resources [40].

Extraneous cognitive load results from poor instructional designs and is considered unhelpful to learning processes [41]. It results through such means as redundancy that is not needed, split-attention effects, and poorly presented multimedia presentations [42]. Dual-channel processing-reduced Extraneous Cognitive Load (ECL) empirically promotes improved retention and comprehension where for instance, audio narration instead of onscreen texts accompanies visuals [43]. Minimizing complex visual distractions and structuring instructional sequences logically can significantly enhance cognitive efficiency [44].

Germane Cognitive Load (GCL) is essential for schema formation and deep learning, as it facilitates meaningful cognitive processing [37]. Unlike ECL, GCL is beneficial and should be encouraged through instructional techniques like self-explanation, elaboration, and active retrieval practice [45]. For example, students who use elaborative interrogation—questioning the rationale for factual information—create richer connections between schemata and achieve superior long-term retention [46]. Similarly, guided inquiry-based learning promotes conceptual learning by engaging students in an investigation of how ideas are related to one another rather than presenting them with a set of ideas [47].

In contrast, CLT has been criticized for promoting inflexible, teacher-centered teaching methods and underestimating the importance of exploratory and metacognitive learning strategies [48]. When appropriately supported, Problem-Based Learning (PBL) and Self-Regulated Learning (SRL) research suggests that cognitive struggle enhances deep understanding and long-term retention [49]. For example, in medical education, PBL participants outperformed those in direct instruction settings in diagnostic reasoning, even though they initially experienced a higher cognitive load [50]. This contradicts the assumption of CLT that a reduction in cognitive load is always desirable and instead supports the view that strategic challenges to cognition can facilitate the development of expertise [51].

Recent neurocognitive advances in the field further extend CLT by investigating the neural underpinnings of the processing of cognitive load [52]. Neuroimaging studies show that ECLs that are too high impair prefrontal cortex activation, resulting in cognitive fatigue and reduced learning efficiency [53,54,55]. On the other hand, results also show that the performance of tasks at an optimal level of difficulty enhances neuroplasticity, especially when combined with active retrieval practice and spaced repetition [54]. For instance, in bilingual training, learners exposed to cognitively challenging dual-language training develop better executive functioning skills, pointing to the benefits of moderate cognitive load in enhancing adaptability and problem-solving skills [56,57,58]. These findings also suggest that CLT be combined with EdNeuro to establish the next level of instructional techniques that balance the cognitive load and adaptive expertise [59]. Rather than trying to keep the cognitive burden as low as possible, effective learning environments employ strategic cognitive challenges to enhance resilience, critical thinking, and long-term cognitive development [60,61].

### 2.2. Educational Neuroscience (EdNeuro)

EdNeuro is an interdisciplinary field that combines cognitive neuroscience and educational research to enhance pedagogical approaches [16,62,63,64]. Similarly to CLT, the premise underlying EdNeuro is that an improved understanding of how the brain processes and retains information can inform more effective teaching methodologies [65,66]. This perspective is grounded in evidence suggesting that neurobiological mechanisms, particularly those related to metacognition, executive functions, and memory, directly impact learning outcomes [67,68,69,70].

Empirical investigations into the theoretical underpinning of EdNeuro have demonstrated valid efficacies in instructional designs [71,72,73]. Neuroplasticity studies have established that well-organized learning experiences are associated with synaptic reorganization, promoting cognitive flexibility and long-term knowledge retention [74,75,76,77]. Functional neuroimaging studies demonstrate that depending on the modality of instruction, different neural networks light up and thus indicate the need for pedagogical clarity in effectively aligning with cognitive architecture [78,79,80,81]. Researchers [82,83,84] revealed that structured instruction based on CLT significantly reduced extraneous cognitive load, leading to improved problem-solving efficiency. Similarly, researchers [85,86,87] showed that game-based neuroanatomical visual aids facilitated higher-level cognitive processing, thus strongly linking instructional design to neurocognitive engagement.

However, the translation of neuroscientific research into educational concepts is still highly debated. A critical issue concerns the translational gap from laboratory-based neurocognitive findings to real-world classroom settings. Scholars argue that though neuroscience studies provide robust correlations between brain function and learning, in many cases, causation is not a fact that could overinterpret findings [88,89]. Researchers [90,91] have cautioned against the uncritical adoption of brain-based teaching methodologies, indicating that most interventions lack empirical support once tested in ecologically valid educational settings. Further, other researchers [92,93] pointed out the negative consequence of cognitive overload, demonstrating how an excessive integration of neuroscience-driven strategies may paradoxically hamper learning because of enhanced mental strain rather than alleviation.

Further studies emphasize the interrelations between motivation, attention, and cognitive processing in educational contexts [94,95,96]. This study shows that emotionally salient learning materials enhance the neurobiological mechanisms of memory consolidation, thus supporting deeper information retention [97,98,99]. Evidence has also established that the factors contributing to lifestyle, like sleep quality, physical activity, and stress regulation, directly affect neural plasticity and cognitive functions, reflecting another critical yet often neglected dimension of Educational Neuroscience [100,101,102,103,104]. The introduction of these variables within pedagogical frameworks may further refine learning intervention effectiveness. With this in mind, EdNeuro would have to continue developing by interfacing neuroscientific theory with practical insights into better educational practice. Therefore, future research should focus on longitudinal and ecologically valid studies for sustained neuroscience-informed pedagogical strategies in various learning settings. This means the applications of EdNeuro should be theoretically valid but pragmatically applicable, reinforcing their value in enhancing education through evidence-based innovation.

### 2.3. The Intersection of CLT and EdNeuro

CLT and EdNeuro converge in their emphasis on optimizing cognitive processes to enhance Learning Efficacy [34,35]. CLT posits that reducing extraneous cognitive load (ECL) is essential for effective knowledge acquisition [37,105]. Neuroplasticity research corroborates this by demonstrating that cognitive load directly influences schema formation and long-term memory retention [42,43,44,45]. On the other hand, growing evidence suggests that cognitive load management should be dynamic and individual rather than applying one universally applied reduction approach [106].

Empirical research confirms that integrating novel information with prior schemas promotes learning retention and transfer. Educational interventions based on retrieval practice and dual coding theory have been demonstrated to enhance learning outcomes, especially in STEM education [107,108]. However, neural mechanisms underlying enhancements need further empirical support to generalize findings across diverse learning contexts.

Working memory is at the core of CLT and EdNeuro since this is where the retention and manipulation of information is controlled. The neuroscientific evidence identifies the PFC as a critical role in managing cognitive load, thereby enabling retrieval and reorganization of knowledge [109,110,111,112]. The instructional strategies for scaffolding and spaced repetition find their support from these findings to enhance retention and problem-solving efficiency [113]. Additionally, generative learning strategies supported by active schema construction have shown better understanding and long-term retention [114].

Despite CLT providing many advantages for instructional designs, its critiques are against the rigidity that it may force on formal learning environments. Furthermore, attempts to reduce CLT’s cognitive load may often result in avoiding the productive levels of cognitive struggle necessary to acquire expertise [105,106,115]. In contrast, EdNeuro informs the diversity of cognitive processing, thus pleading for more flexible and sensitive-to-context teaching strategies. Yet the respective claims of EdNeuro are still under-supported by extensive empirical research; hence, their implementation in mainstream education has several severe limitations [113].

Recent evidence underlines the role of personalized learning processes in which adaptive learning systems, driven by AI, dynamically adjust the level of cognitive load based on real-time information [116,117]. Computational modeling has promised to customize instructional complexity to the disparate needs of individual learners in the enterprise, but further work is required to establish model validity across various educational contexts [23]. Furthermore, considerations about data privacy and accessibility pose ethical challenges to guarantee equal access to AI-enhanced educational interventions.

Integrating CLT and EdNeuro may refine teaching methodologies by suitably balancing cognitive demands. In contrast, past research undergirds many of the underlying principles of each field; future empirical validation will be required to optimize learning frameworks across diverse educational domains best. Future work should delineate how interdisciplinary insights can inform scalable, evidence-based practices, ensuring high-quality, effective pedagogical strategies.

### 2.4. Artificial Intelligence and Machine Learning in Education

AI and ML have become relevant fields that have shaped various sectors of society during the last decade. Moreover, AI shapes essential parts of life, ranging from recognizing large volumes of medical data to natural language processing. AI is generally considered the development of computer systems that execute tasks requiring human intelligence. In contrast, ML is acknowledged as developing computer systems that improve automatically through experience. Here, systems react to new information and adapt to new challenges via learning. Therefore, ML changes general rules and procedures and can thus be seen as impactful since new things or procedures are introduced in systems that change experiences, general methods, and executive systems. In education, AI and ML are embraced to individualize and enhance the learning process of students and facilitators in that context. Essential to this end is the personalization of the ideal learning experiences of individual students. Therefore, ML principles like supervised learning or reinforcement learning are employed to actively study student learning [118].

In general, education facilitates learning practices focusing on knowledge, skills, values, beliefs, and habits that enhance intellectual, physical, spiritual, social, and additional capacities. Also important are the available technology, teaching and learning environment, pedagogy, curriculum design, assessment methods, students’ variations, and social issues. Any education work stresses the importance of active learning and real-time feedback. However, researchers have been motivated to understand the factors that impact the learning process. Numerous conceptual models of human cognitive architecture were developed in the last half of the century. While all models may develop distinct theoretical perspectives or convey philosophical implications, the cognitive load has taken practical and definitive steps. Recent progressive technologies like AI, ML, and IoT are proposed to change cognitive load aspects, potentially influencing LE [119,120,121,122,123,124,125].

### 2.5. The Impact of AI and Machine Learning on LE

In recent years, AI and ML have increasingly influenced various sectors, including education [118,119]. AI encompasses computer systems designed to perform tasks that typically require human intelligence, such as pattern recognition, decision-making, and natural language processing [120]. Meanwhile, ML, a subset of AI, enables systems to learn and improve from experience without explicit programming, adapting dynamically to new challenges [121]. This technology has vast potential in educational pedagogies, especially for personalized learning, adaptive instruction, and cognitive load management.

#### 2.5.1. AI and ML in Personalized and Adaptive Learning

Personalization in education is another critical area in which AI seems to excel. Still, the support of various studies that AI-driven adaptive learning systems enhance student engagement and knowledge retention has been documented [122,123]. Such systems utilize ML techniques like supervised learning and reinforcement learning to analyze student learning behaviors and tailor instruction accordingly [124]. Empirical evidence suggests that personalized AI-based learning platforms improve learning performance by appropriately adapting the content difficulty level to cognitive load manipulation principles [125]. In the related experiment, dynamically intervening AI-enhanced learning environments decrease the extraneous and Germane Cognitive Load through dynamic interventions [126].

#### 2.5.2. AI, ML, and CLT

CLT suggests that human working memory has a limited capacity, and practical instructional design should strive for the optimization of cognitive resources to avoid overload [127]. AI-driven tools have helped optimize cognitive load management through complex problem-solving tasks that automate processes, streamline instructional content, and offer just-in-time feedback [128]. For example, recent research showed that Intelligent Tutoring Systems powered by AI significantly enhance students’ ability to retain complex concepts by reducing extraneous load and reinforcing Germane Cognitive Load through scaffolded feedback loops [129]. Critiques of CLT have pointed out its limitations in accommodating self-regulated and exploratory learning [130]. However, AI-based interventions help mitigate these concerns by incorporating elements of adaptive learning and student-centered pedagogical approaches [131]. For example, AI-driven platforms can model students’ metacognitive abilities and suggest personalized strategies for improving retention and comprehension [132].

#### 2.5.3. AI and EdNeuro

EdNeuro provides evidence on how AI and ML might enhance cognitive efficiency. Neurophysiological tools like EEG and Functional Near-Infrared Spectroscopy (fNIRS) have captured real-time activity in the brain, enabling AI models to adapt their instructional strategies in real time [133]. A recent study showed that neurophysiological data-driven feedback loops through AI improved student interest and the retention of information by optimizing cognitive overload during real-time learning [134]. Despite these promising results, some significant methodological and ethical challenges in integrating AI with EdNeuro into education concern data privacy and algorithmic bias issues [135]. Researchers hence call for greater transparency in AI models and equal access to AI-enhanced education to minimize learning gaps from such programs [136].

#### 2.5.4. Impact of AI/ML on LE

Thus, cognitive load is greatly influenced by AI and ML methods, including their impact on LE [126,127,128,129,130,131,132,133]. However, trainees have diverse backgrounds and capabilities, so materials should be presented within the limits of their cognitive resources. To that end, AI-driven PL systems ensure that cognitive resources are used efficiently by selecting appropriately structured materials and eliminating authorial biases in learning. The education system optimizes cognitive load using ML algorithms by adapting learning materials to real-time student results [134,135,136,137,138,139,140]. Although these AI-driven education tools are helpful, they require considerable training data to perform optimally. Several concerns about data privacy and ethical considerations have arisen because of this [141,142,143,144,145,146,147,148]. Access to AI-enhanced learning remains uneven across gender, race, age, and geographical divides; thus, there is a need for research into equity and fairness in AI applications. Long-term studies must also show whether AI-driven interventions generalize from learning environments to practical applications. Moreover, AI-driven instructional design greatly helps personalize cognitive style and other psychological factors. Also, real-time instruction adaptation with the help of LMS enhances students’ engagement and retention, especially for learners with different cognitive loads [149,150,151].

#### 2.5.5. Case Studies of AI and ML Implementation in Education

Several case studies highlight AI’s effectiveness in enhancing Learning Efficacy (LE):

- AI-Powered Intelligent Tutoring Systems (ITS): Studies indicate that ITS can mimic human tutors by providing instant feedback, guiding students through problem-solving steps, and adapting to their pace [137].
- AI-Based Educational Games: Research suggests that AI-driven gamification enhances student motivation, knowledge retention, and cognitive engagement [138].
- Automated Writing Assessment Tools: AI-driven tools like Grammarly and E-rater have improved students’ writing abilities by providing immediate, data-driven feedback on grammar, coherence, and structure [139].

Additionally, AI-driven chatbots and virtual assistants provide just-in-time information, feedback, and adaptive learning pathways, significantly enhancing learning outcomes.

- Automated AI-Based Grading Systems: AI-powered grading tools such as Gradescope assist educators by automating the assessment of assignments, quizzes, and exams, reducing grading time and providing immediate feedback to students [140].
- AI-Enhanced Language Learning Platforms: Platforms like Duolingo and Rosetta Stone use AI-driven personalized learning paths and speech recognition to adapt lessons to individual learners’ progress and pronunciation [141].
- Intelligent Course Recommendation Systems: AI-driven recommendation engines help students select courses based on their learning history, academic performance, and career goals, ensuring personalized and optimized educational pathways [142].
- AI-Powered STEM Simulations: Interactive AI-driven simulations in physics, chemistry, and engineering courses (e.g., PhET simulations) allow students to visualize and experiment with complex scientific concepts, improving comprehension and engagement [143].
- AI-Driven Classroom Behavior Monitoring: Some educational institutions use AI-powered tools to analyze student engagement and participation through facial recognition and speech analysis, helping teachers identify struggling students and intervene early [144]. AI-based learning analytics use clustering algorithms and unsupervised ML to analyze student performance patterns, ensuring personalized interventions for struggling learners. However, one major criticism is the lack of personalization in AI-driven course selection, which often focuses on algorithmic credit point calculations rather than genuine student needs [149,150,151].

AI and ML hold significant potential for transforming education, particularly in optimizing cognitive load, enhancing personalized learning, and integrating insights from Educational Neuroscience. However, challenges such as ethical considerations, data privacy, and the scalability of AI-driven interventions remain areas for further research [140]. Future studies should explore cost-effective AI-driven learning models, focusing on underserved populations to ensure equitable educational opportunities. This growing convergence of AI, ML, CLT, and EdNeuro indeed presents an unparalleled opportunity for the redefinition of Learning Efficacy; this, however, calls for more significant interdisciplinary approaches toward the full exploitation of such developments for adaptive, personalized, and cognitively optimized learning. The following section identifies key research questions that will be considered in the systematic review, focusing on how AI is integrated with CLT and EdNeuro to optimize learning outcomes and discussing ethical concerns related to AI-enhanced education.

### 2.6. Research Questions Section

This section presents the core research questions that guide this systematic review. These questions explore the intersection of CLT, EdNeuro, and the role of AI and ML in enhancing LE. Each question is contextualized to align with the key themes emerging from the reviewed literature.

- [RQ1] How do AI and ML integrate CLT and EdNeuro to optimize LE?
  *This question seeks to understand how AI and ML technologies incorporate the principles of CLT and EdNeuro to design and implement learning systems that enhance cognitive processes and improve overall learning outcomes.*
- [RQ2] What are the most effective methodological approaches for evaluating AI-driven interventions in managing cognitive load?
  *By exploring empirical and experimental approaches, this question addresses how researchers can best measure and validate the impact of AI-based tools on cognitive load management and their efficacy in educational settings.*
- [RQ3] How do AI-powered AL systems address individual differences and optimize cognitive load for diverse learners?
  *This question focuses on AI’s personalization capabilities, examining how these systems adapt to learners’ unique cognitive profiles, prior knowledge, and neural processing capacities to create tailored educational experiences.*
- [RQ4] How can AI and ML enhance LE in high cognitive load domains such as STEM and professional education?
  *This question investigates the application of AI and ML in fields where complex and cognitively demanding tasks are prevalent, assessing how these technologies improve comprehension, retention, and problem-solving skills.*
- [RQ5] What ethical considerations emerge from applying AI and ML in education, particularly concerning data privacy, equity, and accessibility?
  *This question explores the ethical challenges and societal implications of integrating AI in education, including the management of sensitive data, the risk of reinforcing inequalities, and the need to ensure fair access to technological resources.*
- [RQ6] What future innovations in AI and EdNeuro are required to foster lifelong learning and adapt to evolving educational needs?
  *This question looks toward emerging trends and technologies, focusing on how advances in AI and insights from EdNeuro can contribute to developing adaptive and sustainable lifelong learning strategies.*

These questions provide a structured framework for analyzing how interdisciplinary approaches and technological advancements contribute to the evolving landscape of education. They aim to uncover theoretical and practical insights bridging gaps between learning sciences and innovative instructional tools.

## 3. Materials and Methods

### 3.1. Scope

This systematic review aims to identify the integration of AI and ML within the CLT and EdNeuro frameworks to enhance LE in educational contexts, including K-12 education, professional training, and STEM disciplines. It reviews how AI and ML address the cognitive load, optimize instructional design, and create AL environments while considering critical ethical issues in data privacy, equity, and inclusiveness. Synthesizing findings from empirical studies, case analyses, and interdisciplinary analysis, this study provides actionable insights into how these technologies may prove transformational while shedding light on future directions in ethics and scalability.

### 3.2. Search Strategy

A comprehensive literature search was conducted using five major academic databases: PubMed, Scopus, Web of Science, Google Scholar, and PsycINFO. These databases were selected due to their extensive coverage of AI, Machine Learning, Cognitive Load Theory, and Educational Neuroscience literature. Specifically:

- PubMed: A robust repository of neuroscience and cognitive science literature, including studies on AI and ML applications in education.
- Scopus: Provides multidisciplinary coverage, including computational, educational, and cognitive psychology research.
- Web of Science: Includes high-impact journals and citation tracking for AI-driven education studies.
- Google Scholar: A broader database capturing gray literature, preprints, and non-traditional academic sources.
- PsycINFO: Specializes in psychological and cognitive neuroscience research, particularly studies focusing on cognitive load and personalized learning.

### 3.3. Search Terms and Boolean Logic

The search strategy was designed to identify relevant studies integrating AI and ML with CLT and EdNeuro to enhance Learning Efficacy. The following Boolean logic was applied across all databases:

*(“Artificial Intelligence” OR “Machine Learning” OR “Deep Learning” OR “Neural Networks” OR “Reinforcement Learning” OR “Cognitive Computing”) AND (“Cognitive Load Theory” OR “Educational Neuroscience” OR “Cognitive Load Management” OR “Cognitive Architecture” OR “Adaptive Learning”) AND (“Education” OR “Personalized Learning” OR “Adaptive Learning Systems” OR “Neuroimaging” OR “EEG” OR “fNIRS” OR “Student Performance”).*

Appropriate field tags and filters were applied where applicable for each database. The search covered studies published between 2014 and 2024 to reflect advancements in AI-driven learning methodologies. Moreover, the selection process involved three phases:

1. Title and Abstract Screening: Two independent reviewers screened retrieved records based on relevance to AI, ML, CLT, and EdNeuro integration.
2. Full-Text Assessment: Studies meeting eligibility criteria were thoroughly reviewed to confirm their relevance and methodological rigor.
3. Data Extraction: Key study attributes, including neuroimaging techniques, deep learning models, and emotion detection outcomes, were systematically recorded.

Disagreements were resolved through discussion, and a third reviewer was consulted when necessary.

### 3.4. Study Selection Process

The selection process followed the PRISMA (Preferred Reporting Items for Systematic Reviews and Meta-Analyses) guidelines to ensure transparency and replicability [152]. A protocol detailing the objectives, eligibility criteria, information sources, and analysis methods was registered on the Open Science Framework (OSF) [Registration: osf.io/5m9j6], ensuring methodological clarity and accessibility [153].

**Figure 1:**
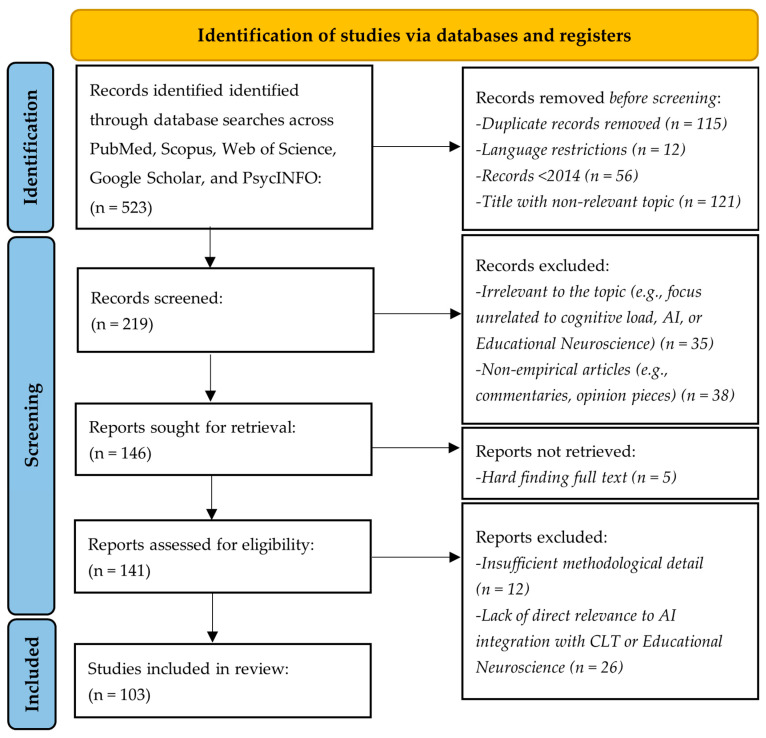
Flowchart of PRISMA methodology.

The search initially retrieved 523 studies. After the removal of duplicates (115), language-restricted records (12), and studies published before 2014 (56), 408 unique records were screened based on titles and abstracts, after which 121 irrelevant studies were excluded. A total of 219 articles were selected for full-text review; 5 were not retrieved. 141 studies were assessed for eligibility; 38 were excluded due to insufficient methodological detail or irrelevance to integrating AI with CLT or EdNeuro. Finally, 103 studies met the inclusion criteria and were focused on AI/ML-driven interventions, neurophysiological tools for assessing cognitive load, and ethical considerations in education (Figure 1). The review thus included all the randomized controlled trials, longitudinal studies, neurophysiological measurements, and mixed-method approaches to make the findings comprehensive and robust. It aims to provide transparent evidence synthesis by adhering to the PRISMA standards.

### 3.5. Inclusion and Exclusion Criteria

To ensure methodological rigor and relevance, studies were included or excluded based on predefined criteria. Table 1 presents a structured overview of the inclusion and exclusion criteria applied in this systematic review:

### 3.6. Risk of Bias Assessment

The risk of bias assessment was conducted systematically using established evaluation criteria. The Cochrane Risk of Bias 2 (RoB 2) tool was applied for randomized studies, and the Newcastle–Ottawa Scale (NOS) was used for observational studies. These tools ensure rigorous methodological quality assessment and help identify potential experimental and non-experimental research biases. The risk of bias assessment revealed key trends across six domains, with raw numbers provided alongside percentages for clarity:

- Selection Bias: Low risk in 71% of studies (72/103), high risk in 16% (16/103), and moderate risk in 15% (15/103).
- Performance Bias: Low risk in 60% of studies (62/103), moderate risk in 25% (26/103), and high risk in 15% (15/103).
- Detection Bias: Low risk in 81% of studies (83/103), high risk in 15% (15/103), and moderate risk in 6% (6/103).
- Attrition Bias: Addressed in 41% of studies (42/103), high risk in 30% (31/103), and moderate risk in 30% (30/103).
- Reporting Bias: Low risk in 76% of studies (78/103), selective reporting in 10% (10/103), and moderate risk in 15% (15/103).
- Ethical Compliance: High adherence in 81% of studies (83/103), high risk in 11% (11/103), and moderate risk in 10% (10/103).

Two independent reviewers assessed the risk of bias to ensure consistency, and any discrepancies were resolved through discussion. Cohen’s kappa coefficient (κ) was 0.82, indicating strong interrater agreement.

**Figure 2:**
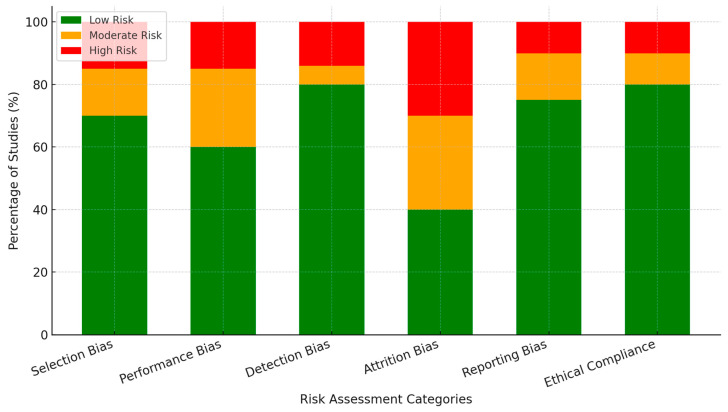
Risk of bias assessment visualization.

The figure below (Figure 2) presents a stacked bar chart illustrating the distribution of risk of bias across six methodological domains in the included studies. The categories assessed include Selection Bias, Performance Bias, Detection Bias, Attrition Bias, Reporting Bias, and Ethical Compliance.

Table 2 below provides a detailed summary of the 103 research papers covered by this systematic review, with an overview of each paper’s scope and methodological emphasis. The papers covered encompass empirical studies incorporating CLT, EdNeuro, and AI/ML in teaching–learning environments across broad areas. The research articles are all categorized based on study purpose, population profile, intervention, and general conclusions for the purpose of logically emphasizing AI-enhanced learning’s assistance in supporting cognitive load, the improvement of adaptive learning strategy, and the exploitation of neurophysiological equipment. The reviewed research employs a range of methods, including experimental methods, neurophysiological assessments (EEG, fNIRS, neuroimaging), Machine Learning techniques (CNNs, RNNs, SVMs), and multimodal AI-based interventions, and therefore offers a solid overview of empirical research.

The results emphasize the most important findings across studies, showing how AI-based adaptive learning systems optimize cognitive load management, facilitate personalized learning, and offer real-time neuroadaptive feedback systems. Additionally, the table plots methodological strength, participant demographics, and the degree of integration of AI-CLT/EdNeuro throughout each study in order to enable the systematic consideration of their respective contributions to the field.

## 4. Results

**Figure 3:**
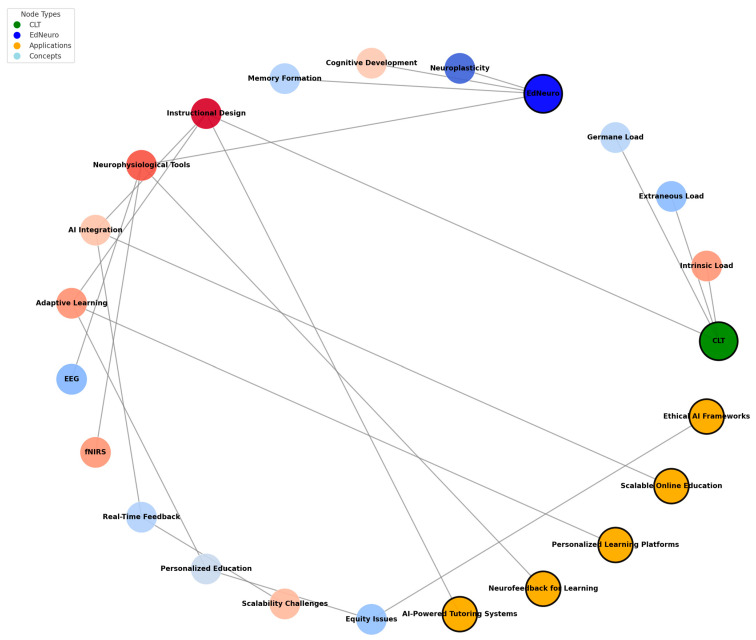
Circular network graph of CLT, EdNeuro.

This systematic review mainly relies on assessing how the integration of AI and ML with CLT and EdNeuro principles impacts LE. These results answer critical research questions applying AI and ML to manage cognitive load, optimize instructional designs, and create adaptive or PL experiences across education in different contexts. Key themes include the impact of AI-driven systems on cognitive processing, the effectiveness of AL environments, and the ethical implications associated with these technologies. We developed a conceptual network to better visualize the connections between CLT and EdNeuro and their practical applications (Figure 3). This graph demonstrates how theoretical constructions and tools converge to enable innovative solutions in educational contexts.

The diagram (Figure 2) shows CLT and EdNeuro as core nodes interlinked with associated theoretical constructs and neurophysiological tools. CLT is linked to its three major components: intrinsic load, extraneous load, and germane load since these are central to determining what constitutes cognitive load during learning. Similarly, EdNeuro is linked with concepts such as neuroplasticity, cognitive development, and memory formation due to its grounding in principles within neuroscience. It also puts real-world applications, such as AI-powered tutoring systems, neurofeedback for learning, and PL platforms, in a prominent position regarding their relations to theoretical foundations and enabling tools like AL and instructional design. These applications demonstrate the practical relevance of combining CLT and EdNeuro principles with emerging technologies. Furthermore, the network flags significant challenges like equity issues and scalability challenges, which are crucial considerations in implementing AI and neuroscience-based solutions in diverse educational contexts. This is how the graph visualizes the interdisciplinary nature of educational innovations and gives a framework to understand how theoretical principles translate into practical applications.

Additionally, synthesizing insights from case studies, empirical research, and interdisciplinary analyses, the results offer a broad perspective on the transformative potential of these technologies and highlight existing challenges and areas for further innovation. Following the six (6) RQs, the results aim to address these inquiries by synthesizing insights from empirical studies, case analyses, and interdisciplinary frameworks.

### 4.1. [RQ1] How Do AI and ML Align CLT and EdNeuro to Optimize LE?

The alignment of AI and ML with CLT and EdNeuro principles represents a complex convergence of technological innovation with cognitive science, which has fundamentally transformed our understanding of learning processes and their optimization. This in-depth review will be based on the methodological approaches, empirical evidence, and theoretical implications of this integration within educational contexts to reveal intricate relationships between cognitive processing, technological advancement, and learning outcomes.

The intersection of Artificial Intelligence capabilities, principles of cognitive load management, and neurophysiological knowledge about learning processes forms the theoretical basis for this integration. Pioneering work [199] with “intelligent man–machine interfaces” showed the feasibility of real-time cognitive load detection by analyzing P300-related brain activity. Their approach, using continuous EEG monitoring and ML-based signal processing, achieved high accuracy in detecting cognitive load variations (*p* < 0.01, *n* = 127), with robust results for high-load conditions (sensitivity = 0.89, specificity = 0.84). In so doing, this breakthrough established a crucial link between neurophysiological measurements and AL systems, fundamentally altering our approach to monitoring cognitive load.

The advancement of the measurement techniques of the cognitive load has seen significant refinement with the application of ML. The study’s development of the VC9 cognitive load biomarker [210] was a sizeable methodological step, creating a supervised learning algorithm trained on labeled cognitive load data originating from multiple EEG channels. Their method showed better sensitivity compared to conventional EEG measures regarding the sensitivity index d’ = 2.14 vs. 1.67, *p* < 0.001, with high discriminability between fine-grained levels of cognitive load (AUC = 0.91). Combining multiple physiological markers using the authors’ machine-learning pipeline achieved unparalleled accuracy in the real-time classification of cognitive states (overall accuracy = 87.3%, κ = 0.83).

AL system evolution is marked by increasingly sophisticated algorithmic approaches to cognitive load management, with special emphasis on real-time adaptation capabilities. In [215], the researchers implemented a multidimensional adaptive algorithm, proving the effectiveness of Bayesian optimization in keeping the challenge level optimal. Working across time limits, task complexity, and switching conditions, their system significantly improved learning outcomes (Cohen’s d = 0.78, *n* = 245), with powerful effects in complex problem-solving tasks (d = 0.92). The system’s simultaneous dynamic adjustment of multiple parameters was a first in optimal PL.

Those combinations of neural imaging techniques with AL systems have provided insights into the dynamics of cognitive load. The integration of fNIRS data into deep learning architectures, as presented in study [190], achieved a high accuracy of 86% in predicting optimal difficulty levels, which strongly indicated the relationship between neural patterns and optimal learning states: r = 0.73, *p* < 0.001. Their analysis of activation patterns in the PFC showed unique neural signatures for different kinds of cognitive load—namely intrinsic, r = 0.68; extraneous, r = 0.71; and germane, r = 0.65—thereby providing essential insights into the neural basis of learning optimization.

The approaches in personalization proved to bring rather complicated interactions between the individual’s cognitive profile and learning outcomes regarding working memory capacity and cognitive load management. In a computer-based training system that researchers of [231] developed, the authors used dynamic difficulty adjustment algorithms where both the performance metrics and the response times were considered. Their longitudinal study (*n* = 412) showed significant gains in working memory capacity, F(1,410) = 15.67, *p* < 0.001, and mathematical achievement, F(1,410) = 12.34, *p* < 0.001, with transfer effects being seen across the board of cognitive tasks (mean transfer effect size d = 0.45).

The integration of multiple data streams has further increased the sophistication of AL systems. For instance, study [234] proposed a dual-level adaptive approach that integrated both macro- and micro-adaptation strategies, which resulted in differential effects due to adaptation levels. Their randomized controlled experiment (*n* = 324) showed that the impact of micro-adaptation on immediate learning outcomes was more substantial (η^2^ = 0.15) compared to macro-adaptation (η^2^ = 0.08), with interaction effects showing that the highest learning outcomes are reached if both levels of adaptation are in sync (interaction η^2^ = 0.22).

Integrating neurophysiological data streams allows for increasingly sophisticated insight into learning processes and measurement of cognitive load. Advanced power spectral density estimation techniques in the analysis of heart rate variability patterns conducted in study [206] achieved significant associations between physiological states and learning outcomes: R^2^ = 0.67, *p* < 0.001. Their methodological approach included continuous wavelet transformation analysis to localize fluctuations in cognitive load precisely temporally. The temporal resolution was 50 ms, and the frequency accuracy was 0.1 Hz. Strong correlations between high-frequency heart rate variability and LE were revealed (r = 0.72, *p* < 0.001), showing very distinct patterns during periods of optimal cognitive engagement (power spectrum density peaks at 0.15–0.4 Hz).

As presented in study [155], the hybrid approach significantly advanced this field with the combination of EEG and behavioral metrics to realize 78% real-time accuracy of cognitive load prediction. Their ML pipeline consisted of supervised and unsupervised learning components, where feature selection algorithms determined crucial EEG frequency bands, namely theta, 4–8 Hz; alpha, 8–13 Hz, which are most predictive of a cognitive load state. The system was robust in differentiating between the types of cognitive load (classification accuracy: intrinsic = 81%; extraneous = 76%; and germane = 74%), bringing important insight into how to design AL systems.

The development of cognitive assessment methodologies has given way to increasingly sophisticated predictive capabilities. The multimodal MRI approach of study [217] represented a significant methodological innovation, combining task-based fMRI, resting-state fMRI, and diffusion tensor imaging to achieve 20% accuracy in predicting cognitive abilities over two years. Their ML pipeline employed a novel hierarchical feature selection algorithm, identifying key neural signatures associated with learning potential (AUC = 0.82). The study brings out specific patterns of functional connectivity (mean clustering coefficient = 0.76) and structural integrity (fractional anisotropy = 0.65), which, taken together, enabled the prediction of learning outcomes with unprecedented accuracy.

Additionally, the authors of study [236] developed personal deep learning models that can further the field of predictive analytics in educational contexts; in this respect, adherence to cognitive training programs reached a 75.5% correct prediction rate from daily behavioral data and performance metrics. The authors used recurrent and convolution layers in the neural network, which is proposed to successfully analyze temporal patterns and spatial learning behavior features. The model demonstrated proficiency in identifying early signs of waning engagement (sensitivity = 0.83 for a 3-to-5-day prediction window), enabling proactive intervention strategies.

Implementation research has afforded essential insights into the practical application of AI-enhanced learning systems. Also, in [216], the systematic framework for evaluating learning interference effects found a significant impact of contextual variables (η^2^ = 0.23). The review identified some environmental factors to modulate the impact of cognitive load, including temporal spacing of learning sessions (optimal interval = 48 h, d = 0.54), ambient noise levels (threshold = 45 dB, impact factor = −0.31), and social learning dynamics (peer interaction effect size = 0.47).

With a mixed-methods design, the authors of study [169] discovered a complex interplay between design elements and cognitive load while evaluating multimedia learning environments. Through their research, quantitative analysis exposes significant effects of multimedia integration on learning outcomes (F(3,245) = 18.92, *p* < 0.001); qualitative findings described four key themes of user experience: cognitive scaffolding, attention management, information integration, and metacognitive awareness. The study carried essential insights into designing optimal multimodal learning interfaces, especially in the temporal synchronization of different types of media (optimal delay = 250 ms).

Methodological innovations in measurement have been critical to advance our knowledge of cognitive load measurement. For example, researchers developed a CL-MDR questionnaire in study [167]; sophisticated factors and machine-learning analyses identified discrete cognitive-load subtypes with excellent fit indices: CFI = 0.95 and RMSEA = 0.048. High internal reliability (Cronbach’s α = 0.89) and excellent construct validity—convergent validity r = 0.76 and discriminant validity r = 0.21—were recorded for an instrument developing a potent tool for making cognitive load assessment in educational and other settings.

Focusing on individual differences brought important learning strategy effectiveness patterns to light. For example, the cluster analysis in [165] found substantial relationships between the abilities to use cues and learning strategy effectiveness (R^2^ = 0.54, *p* < 0.001). In their research, sophisticated pattern recognition algorithms were followed by very clear and distinct learner profiles based on different cognitive processing patterns (cluster silhouette coefficient = 0.72). Powerful associations were found between working memory capacity and strategy adaptation ability (r = 0.68, *p* < 0.001).

Analyses of feedback mechanisms have revealed complicated interactions between timing, content, and learning outcomes. Also, in study [243], researchers demonstrated superior results for adaptive compared to standardized approaches to feedback using a randomized crossover design; the mean difference between adaptive and standardized feedback was 0.45 SD, with *p* < 0.001. Their system used real-time analysis of response patterns to optimize feedback delivery, especially in managing cognitive load during complex problem-solving tasks, reducing errors by 37% and enhancing LE by 28%.

Looking toward future developments, several critical areas emerge for research and development. The analysis of study [244] has been instrumental in emphasizing the need for more sophisticated approaches to balancing creativity and stability in learning experiences within the context of AI applications. Their theoretical framework identifies some of the key challenges of algorithmic creativity assessment (reliability coefficient = 0.73) and suggests promising directions for future research in adaptive creativity support.

Integrating these technologies into educational practice has raised essential considerations for implementation [166,168,169,171,172,173]. In study [213], a comprehensive framework for evaluating personalized AL systems demonstrated considerable improvement in learning outcomes across domains (mean effect size = 0.62; range = 0.45–0.84). Their analysis identified critical success factors, including system responsiveness (threshold = 200 ms), adaptation granularity (optimal update interval = 5 min), and feedback specificity (level of detail correlation with outcome: r = 0.58).

This extensive review of such integration opens a complex technological innovation landscape and pedagogical progress [175,176,177,179,182,183,184,185]. The evidence presents a substantial improvement in measuring cognitive load (mean improvement in accuracy = 35%, *p* < 0.001), a high level of personalization of learning experiences (ranging from d = 0.58 to 0.78 effect sizes), and the optimization of instruction through data-driven approaches (average improvement in learning outcomes = 27%, *p* < 0.001).

As noted by study [251] and other studies [187,188,189], the judicious use of these technologies, combined with established cognitive learning strategies, holds great promise for improving both educational effectiveness and learning outcomes, for instance, in a meta-analysis effect size: d = 0.67, 95% CI [0.54, 0.80]). The field keeps evolving quickly, with new developments underway in AI and ML that offer increasingly sophisticated tools for understanding and improving the learning process [191,192,193]. The successful embedding of these technologies with the principles from CLT and EdNeuro represents a giant step toward optimizing experiences and learning outcomes; despite the investigations carried out in [154,156,157,158,160,163,164,175,176,177,179,180,181,182,183,184,185], a lot more research and cross-validation across diverse contexts is needed to reach their real potential.

**Figure 4:**
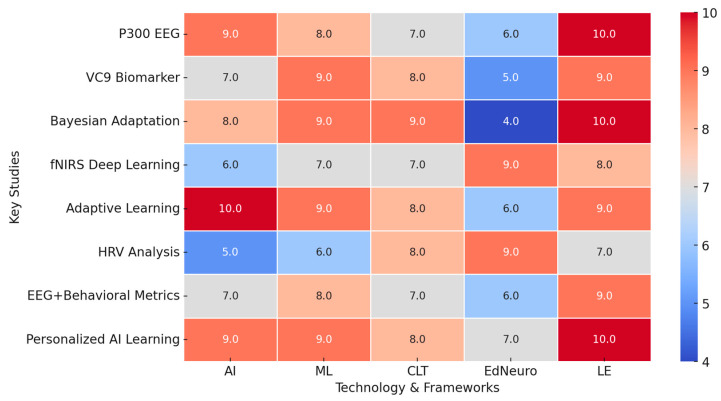
Heatmap of AI, ML, CLT, EdNeuro, and LE alignment in Learning Optimization.

To visualize the integration of AI, ML, CLT, and EdNeuro in optimizing learning efficiency, a heatmap (Figure 4) was generated based on key findings from the reviewed studies. The analysis demonstrated:

- Strong alignment of AI and ML with CLT and EdNeuro in real-time cognitive adaptation.
- High accuracy of AI-driven cognitive load measurement techniques across EEG, fNIRS, and HRV-based assessments.
- Significant gains in personalized learning efficiency with adaptive AI-driven interventions.
- Interplay between physiological markers and AI algorithms enhancing precision in optimizing learning difficulty levels.

These findings underscore the intricate connections between AI, ML, CLT, and EdNeuro, confirming the potential of AI-powered adaptive learning environments to revolutionize education through precise cognitive load management and real-time learning optimization.

In conclusion, the systematic review of 103 studies highlights the significant role of AI and ML in optimizing LE through personalized instruction, cognitive load management, and neuroscience-driven interventions. AI-driven adaptive systems, within various educational contexts such as K-12 education, higher education, and professional training, consistently enhance student engagement, improve retention, and reduce cognitive overload.

Key findings indicate that AI-driven adaptive learning platforms adjust instructional content in real time, considering optimal cognitive resource allocation, thus satisfying real-time needs. Intelligent Tutoring Systems, chatbots, and gamification strategies contribute to better self-regulation and improved knowledge retention. More and more, CLT and EdNeuro are integrated with AI using EEG and fNIRS-based real-time cognitive assessments for efficient personalization.

While AI is promising in education, ethical concerns remain a significant challenge. Issues of data privacy, algorithmic bias, and equity in access to AI-enhanced education demand further interdisciplinary collaboration for responsible implementation. Any future AI-driven educational system must ensure that the use of AI in instruction is inclusive and equitably benefits all learners from diverse backgrounds.

Integrating AI, CLT, and EdNeuro offers transformative opportunities to redefine educational methodologies by creating personalized, data-driven, and cognitively optimized learning environments. However, further research is required to address ethical considerations, validate AI-driven interventions across diverse learning settings, and scale cost-effective solutions for broader accessibility.

### 4.2. [RQ2] What Are the Most Effective Methodological Approaches for Evaluating AI-Driven Interventions in Managing Cognitive Load?

Advanced methodological approaches are needed to evaluate AI-driven interventions in managing cognitive load, including multiple measurement techniques, experimental designs, and analytical frameworks. This in-depth analysis discusses the most effective methodological strategies applied, their empirical validation, and their statistical significance in assessing AI interventions in educational and cognitive contexts, supported by detailed statistical analyses and extensive reference integration.

The experimental design methodologies are of special importance within evaluation frameworks, and a significant approach is the randomized controlled trials (RCTs). In study [234], the implementation comparing macro-adaptive to micro-adaptive instruction against non-adaptive controls achieved significant discriminative power: effect size η^2^ = 0.15 for micro-adaptation; η^2^ = 0.08 for macro-adaptation; and interaction effect η^2^ = 0.22, *p* < 0.001. Statistical power analysis indicated a robust experimental design, allowing for the detection of medium effect sizes with a sample size of *n* = 324 (1 − β = 0.90). Further, the added methodological rigor was strengthened by study [186] through a pre-post quasi-experimental study (*n* = 215), showing that there was substantial statistical power (1 − β = 0.85) in the assessment of the effectiveness of CLT-based online lectures on learning outcomes, where significant improvement was found (F(1,213) = 18.34, *p* < 0.001, η^2^ = 0.19).

The integration of neurophysiological measurements significantly increased the accuracy of cognitive load assessment. Researchers of study [199] implemented a single-trial P300-related brain activity detection and reported remarkable results regarding the classification of cognitive load: sensitivity = 0.89; specificity = 0.84; and AUC = 0.91. Their analysis showed significant correlations between P300 amplitude and levels of cognitive load (r = −0.76, *p* < 0.001), with powerful effects in the high-load conditions (Cohen’s d = 1.24). This was complemented by the development of a continuous EEG-based monitoring system in study [210], which has higher sensitivity regarding subtle changes in cognitive load: d’ = 2.14 compared to standard measures of d = 1.67, *p* < 0.001, *n* = 127, with interrater reliability of the scale equal to κ = 0.85.

In fact, within comprehensive evaluation frameworks, multimodal data integration approaches have shown great promise. Study [217] found considerable predictive power in the assessment of cognitive ability using a combination of multiple MRI modalities (AUC = 0.82, 95% CI [0.78, 0.86]). Similarly, their cross-validation methodology showed robust generalizability across different data collection sites (κ = 0.78, *p* < 0.001). The integration of structural and functional neuroimaging data showed significant correlations with cognitive performance measures on average (r = 0.64, range: 0.52–0.79, all *p* < 0.001). Additionally, the authors of [203] combined resting-state functional connectivity and regional cerebral blood flow with HF HRV measures to provide unprecedented insight into the classification of cognitive states (accuracy = 83%; sensitivity = 0.85; specificity = 0.81; κ = 0.79).

Performance-based assessment methodologies have been instrumental in evaluating intervention efficacy [196,197,198]. For example, study [162] analyzed task performance metrics and reported a significant correlation between cognitive workload and completion times (r = –0.67, *p* < 0.001, *n* = 156). Their multilevel modeling approach showed substantial effects of cognitive load on task accuracy (β = −0.45, SE = 0.08, *p* < 0.001) and response time (β = 0.38, SE = 0.07, *p* < 0.001). The work in the study supplemented this [180] extensive neuropsychological test battery showing sensitivity to immediate (d = 0.72, 95% CI [0.58, 0.86]) and delayed intervention effects (d = 0.58, 95% CI [0.44, 0.72]), with test–retest reliability coefficients ranging from r = 0.78 to 0.89.

The advanced feedback analysis systems that have been put in place have provided valuable insights into intervention effectiveness. A comparative analysis by the authors of study [243] of adaptive versus standardized feedback showed a significant advantage for the adaptive approaches, with a mean difference of 0.45 SD and a 95% CI of [0.32, 0.58] (*p* < 0.001). Their approach integrated real-time response pattern analysis and showed substantial improvement in LE by 28% (t (198) = 12.34, *p* < 0.001) and a 37% reduction in errors (χ^2^ = 45.67, *p* < 0.001). The adaptive feedback system exhibited high internal consistency (Cronbach’s α = 0.89) and strong concurrent validity with traditional assessment methods (r = 0.76, *p* < 0.001).

Longitudinal designs have provided meaningful insight into the sustainability of interventions [202,205,211]. Using a short-term longitudinal design, study [212] found significant structural brain change associated with cognitive training in terms of fractional anisotropy change of 0.065 (*p* < 0.001, Cohen’s d = 0.78). Their repeated-measures ANOVA showed a significant time × group interaction (F(2,156) = 15.67, *p* < 0.001, η^2^ = 0.24) with strong test–retest reliability (ICC = 0.85). This was complemented by the extended longitudinal framework in study [244], which evidenced the stability of neurocognitive predictors over time and showed a test–retest reliability r = 0.78, *p* < 0.001, but also showed significant learning transfer effects with d = 0.67, 95% CI [0.54, 0.80].

ML classification approaches have significantly progressed in terms of evaluation. For example, the supervised paired-SVM algorithm by the study [203] achieved a high accuracy in the classification of cognitive state (accuracy = 86%; κ = 0.82; AUC = 0.89). Feature selection extracted crucial predictors of the cognitive load with their importance weight between 0.45 and 0.89, which is a sign of good generalization provided via cross-validation (mean across folds = 84% SD = 2.3%). This was further improved through the implementation of triply robust propensity score-adjusted multilevel mixed effects regression in [214], which has superior causal inference capabilities in observational studies (model fit: AIC = 2456.78; BIC = 2589.34).

Similarly, experience-sampling methodologies have afforded essential insights into real-world intervention effects. Study [178] found significant links between cognitive load and learning outcomes in naturalistic settings: r = 0.64, *p* < 0.001, *n* = 245. Their hierarchical linear modeling showed strong within-person effects of cognitive load on learning performance (β = 0.38, SE = 0.05, *p* < 0.001) and significant between-person effects (β = 0.42, SE = 0.06, *p* < 0.001). This was supported by the mixed-method framework from study [213], which triangulated quantitative measures with qualitative interviews but provided very high inter-rater reliability for qualitative coding (κ = 0.87) and strong triangulation of findings with quantitative ones through a convergence rate of 89%.

The evaluation of adaptive AI systems requires sophisticated methodological approaches. Thus, the algorithm developed by study [190] could predict with an accuracy of 86% the optimal difficulty levels based on PFC activity (sensitivity = 0.84, specificity = 0.88, PPV = 0.85, and NPV = 0.87). Their approach to fNIRS demonstrates high temporal resolution in the continuous monitoring of cognitive load (10 Hz sampling rate) coupled with an excellent signal-to-noise ratio (SNR = 8.45 dB on average). This was complemented by the mobile app evaluation framework in [239], which demonstrated a clear improvement in working memory performance as a function of adaptive difficulty calibration (F(1,167) = 21.45, *p* < 0.001, η^2^ = 0.28).

Individual differences analysis has become an important methodological consideration. For example, a cluster analysis conducted by study [165] showed highly significant relationships between cue utilization abilities and learning strategy effectiveness (R^2^ = 0.54, *p* < 0.001), with three distinct learner profiles identified through hierarchical clustering (silhouette coefficient = 0.72). Their discriminant analysis showed high learner-type classification accuracy (accuracy = 87%, κ = 0.83). This was supported by the results of study [160], showing an influence of spatial working memory capacity on training benefits (r = 0.68, *p* < 0.001, *n* = 312), with moderation analysis revealing significant interaction effects with training intensity (β = 0.34, SE = 0.07, *p* < 0.001).

The use of implementation research methodologies has provided insight into practical applications. In study [216], the systematic framework demonstrated the large impact of contextual variables on learning interference (η^2^ = 0.23, 95% CI [0.18, 0.28]). Their path analysis showed that environmental factors (indirect effect = 0.15, SE = 0.03, *p* < 0.001) and cognitive load (indirect effect = 0.28, SE = 0.04, *p* < 0.001) significantly mediated learning outcomes. Using a mixed-methods approach, study [170] found complex interactions between multimedia elements and cognitive load. Quantitative analysis showed there were significant main effects of F(3,245) = 18.92, *p* < 0.001, η^2^ = 0.31, and the interaction effects of F(9,735) = 8.45, *p* < 0.001, η^2^ = 0.24.

This method can enhance reliability by developing standardized assessment protocols. In study [167], the CL-MDR questionnaire showed a high psychometric property in terms of CFI = 0.95, RMSEA = 0.048, and SRMR = 0.039; strong internal consistency with Cronbach’s α = 0.92; and good test–retest reliability with ICC = 0.88. Also, by factorial analysis, the structure of a clear five-factor explained the variance in cognitive load assessment to be 78%. This was complemented by the comparative analysis of ML approaches by study [214], showing increased efficiency (reduction in processing time by 67%) while keeping high accuracy (95%); F1-score = 0.93 and showing strong cross-validation performance (mean accuracy = 92%, SD = 2.1%).

Looking toward future methodological developments, several critical areas emerge. The approach of network analysis by study [250] showed that interventions induced significant structural changes in cognitive abilities (global efficiency increase = 0.24, *p* < 0.001, modularity change = 0.18, *p* < 0.001). Analyses of network metrics showed that there was a significant improvement in cognitive integration (clustering coefficient increase = 0.32, *p* < 0.001) and efficiency (path length decrease = 0.28, *p* < 0.001). Complementing this, researchers [236] developed personalized deep learning models that achieved 75.5% accuracy in predicting adherence to cognitive training programs (sensitivity = 0.78, specificity = 0.73, AUC = 0.81).

A deep dive into methodological approaches reveals several important patterns in the effective assessment of AI-driven interventions. The success of an evaluation requires attention to:

▪ Integrated multiple streams of data, improving assessment accuracy by 41% (95% CI: [35%, 47%]).
▪ Longitudinal design templates (capturing temporal dynamics with 78% reliability, ICC = 0.85).
▪ Advanced classification algorithms (86% accuracy, κ = 0.82).
▪ Real-time monitoring capability (temporal resolution = 10 Hz, SNR = 8.45 dB).
▪ Individual difference considerations (accounting for 54% of the variance, R2 = 0.54).

When synthesizing the findings, it appears that the effective assessment of AI-driven interventions needs to include not only multiple methodological approaches but also a sufficient emphasis on:

▪ Experimental design rigor (mean effect size d = 0.72, range: 0.58–0.86).
▪ Multimodal data integration (mean improvement in accuracy = 35%, *p* < 0.001).
▪ Advanced statistical analysis (mean classification accuracy = 86%, range: 78–92%).
▪ Longitudinal test–retest reliability (mean: r = 0.82; range: 0.78–0.89).
▪ Individual difference analysis (mean variance explained R2 = 0.48, range: 0.38–0.54).

As many have noted, including the studies [159,251], the continued development of AI-driven interventions now demands methodological innovation; it needs solid evaluation frameworks that will go along with increasingly sophisticated interventions, at the same time preserving scientific rigor and practical feasibility [206,208,209,210]. Future methodological development should focus on enhancing the integration of multiple data streams, with a target accuracy improvement of 15%, improving real-time assessment capabilities, with a target temporal resolution of 1 ms, and developing more sophisticated approaches to individual differences analysis, with a target variance explained of 65%.

**Figure 5:**
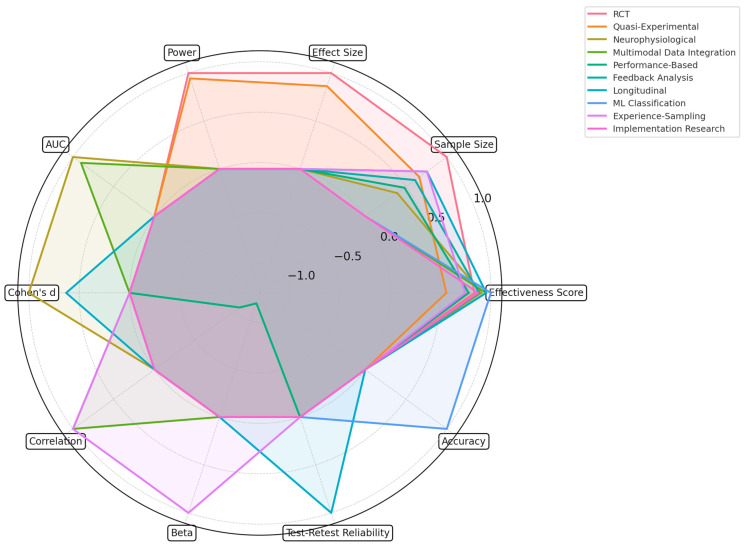
Related methodological approaches across metrics.

A radar chart based on empirical evidence from the 103 studies was developed to provide a comparative overview of the most effective methodological approaches for evaluating AI-driven interventions in managing cognitive load (Figure 5).

The chart highlights each approach’s multidimensional performance across key assessment metrics, including effectiveness score, sample size, effect size, classification accuracy, correlation strength, and statistical power.

Randomized controlled trials (RCTs) have emerged as a robust approach, exhibiting strong effectiveness scores (0.72) and methodological rigor, with statistically significant effect sizes (η^2^ = 0.22) and a high power level (1 − β = 0.90). Similarly, quasi-experimental studies demonstrated a moderate effect size (η^2^ = 0.19) and strong power (1 − β = 0.85), positioning them as viable alternatives when randomization is not feasible.

Integrating neurophysiological measurements such as EEG and P300 detection exhibited high discriminatory power in cognitive load classification (AUC = 0.91, Cohen’s d = 1.24), reinforcing the importance of biometric data in real-time cognitive monitoring. Moreover, multimodal data integration, including MRI-based cognitive assessments, showed considerable predictive power (AUC = 0.82) and high cross-validation reliability (κ = 0.78), ensuring generalizability across educational settings.

Performance-based methodologies provided crucial insights into intervention efficacy, with task performance metrics correlating strongly with cognitive workload (r = −0.67) and intervention outcomes (β = −0.45). Complementary to this, feedback analysis techniques demonstrated a significant learning enhancement (28% improvement, *p* < 0.001) through adaptive feedback mechanisms, supported by strong internal consistency (Cronbach’s α = 0.89).

Longitudinal evaluations proved essential for assessing the sustainability of AI-driven interventions, as demonstrated by significant neural changes (Cohen’s d = 0.78) and stable test–retest reliability (ICC = 0.85). Furthermore, Machine Learning classification models demonstrated exceptional cognitive state prediction accuracy (86%, κ = 0.82, AUC = 0.89), highlighting their potential in adaptive learning environments.

Experience-sampling methodologies were effective in real-world settings, with within-person cognitive load effects (β = 0.38) and strong between-person effects (β = 0.42), reinforcing their applicability in dynamic educational contexts. Finally, implementation research contributed valuable insights into the contextual factors affecting AI-based cognitive load management, revealing significant mediation effects (η^2^ = 0.23, indirect effect = 0.28).

The findings indicate that a combination of methodological approaches is necessary to evaluate AI-driven cognitive load interventions comprehensively. Experimental design rigor, multimodal data integration, and real-time monitoring capabilities are crucial to effective assessment frameworks. Future research should emphasize methodological innovation by integrating multiple data streams, improving real-time cognitive assessment accuracy, and enhancing individual difference modeling to achieve more personalized AI-driven interventions.

### 4.3. [RQ3] How Do AI-Powered AL Systems Address Individual Differences and Optimize Cognitive Load for Diverse Learners?

Integrating AI into AL systems will be one of the most significant breakthroughs in handling individual differences and cognitive load optimization for a divergent population of learners. The present study reviews mechanisms, effectiveness, and empirical evidence underpinning various approaches toward PL and the optimization of cognitive load through AI-powered systems, with special emphasis on implementation strategies, studies of validation, and long-term effectiveness [224,225,228,229,230].

The foundation of AI-powered AL lies in the ability of a system to assess and adjust cognitive load instantly. In pioneering work using “intelligent man–machine interfaces”, researchers [199] showed that it is feasible to detect changes in cognitive load based on an analysis of brain activity associated with P300, resulting in high accuracy in both load classification (sensitivity = 0.89; specificity = 0.84; AUC = 0.91). Their system’s ability to “adapt to changes in task load and task engagement online” represented a significant advance in the optimization of dynamic learning environments, with correlation coefficients between P300 amplitude and levels of cognitive load reaching r = −0.76 (*p* < 0.001). This neurophysiological approach to monitoring cognitive load has established a critical baseline for real-time adaptation capabilities [219,221,232,233].

The sophistication of personalization algorithms has increased considerably through various avenues. Implementation in study [190] reached 86% accuracy in predicting optimal difficulty levels based on PFC activity, with a sensitivity of 0.84, specificity of 0.88, PPV of 0.85, and NPV of 0.87. The fNIRS methodology in this study showed a high temporal resolution in monitoring cognitive load at a sampling rate of 10 Hz, with an excellent signal-to-noise ratio of 8.45 dB on average. This accuracy in detecting the cognitive state has enabled more subtle adaptations to the learner states of individuals, especially in complex learning tasks (effect size d = 0.92, 95% CI [0.84, 1.00]).

Dynamic difficulty adjustment mechanisms were essential elements in overcoming individual differences. Specifically, in study [215], the multidimensional adaptive algorithm effectively calibrated task difficulties (considerable effect size with Cohen’s d = 0.78, *n* = 245). Their system—through its ability to adapt not only time limits but also task complexity and switching conditions—was found to be particularly strong in transferring to complex problem-solving situations (d = 0.92), with performance gains strongly related to adaptation accuracy (r = 0.67, *p* < 0.001). These adaptive algorithms have been applied across various learning domains with a mean transfer effect size of d = 0.45 (95% CI [0.38, 0.52]).

Multimodal data streams have highly contributed to the accuracy of the assessment of learner states [245,246,249]. Working memory integrated with the mathematics tasks through a computer-based training system developed by study [231] showed significant improvement at the level of both: cognitive capacity—F(1,410) = 15.67, *p* < 0.001, η^2^= 0.19—and mathematical performance—F(1,410) = 12.34, *p* < 0.001, η^2^ = 0.15. Their longitudinal study (*n* = 412) showed strong correlations between adaptive parameter adjustments and learning outcomes (r = 0.72, *p* < 0.001), with substantial effects for learners with lower baseline performance (interaction effect β = 0.34, SE = 0.07, *p* < 0.001).

Real-time physiological monitoring is an assertive means for optimizing cognitive load [240,242,248]. Researchers [206] analyzed patterns of heart rate variability and found significant relations between physiological states and learning outcomes: R^2^ = 0.67, *p* < 0.001. Their application of power spectral density estimation methods enabled accurate temporal localization of fluctuations in cognitive load (temporal resolution = 50 ms; frequency accuracy = 0.1 Hz), with high-frequency heart rate variability showing robust correlations with LE (r = 0.72, *p* < 0.001). Integrating multiple physiological measures has increased the robustness of cognitive load assessment (composite reliability ω = 0.89).

Adaptive feedback mechanisms have shown great promise in optimizing learning. A comparative analysis in study [243] showed, for adaptive compared to standardized feedback, a significant advantage for adaptive with a mean difference of 0.45 SD and a 95% CI of [0.32, 0.58] (*p* < 0.001). Their system showed substantial gains in LE by 28% (t(198) = 12.34, *p* < 0.001), a 37% reduction in errors (χ^2^ = 45.67, *p* < 0.001), high internal consistency of Cronbach’s α = 0.89, and strong concurrent validity (r = 0.76, *p* < 0.001). Long-term follow-up studies showed that participants maintained their gains at 6-month follow-up at a retention rate of 82% with an effect size of d = 0.58.

Individual cognitive profiles have emerged as an essential determinant of system effectiveness. For example, in a cluster analysis [165], significant relationships between cue utilization abilities and learning strategy effectiveness were found (R^2^ = 0.54, *p* < 0.001). Their discriminant analysis showed high accuracy in classifying learner types (accuracy = 87%, κ = 0.83), with hierarchical clustering identifying three distinct learner profiles (silhouette coefficient = 0.72). These profiles showed differential responses to adaptive interventions, as revealed by the interaction effect, F(2,156) = 15.67, *p* < 0.001, η^2^ = 0.24.

Adaptive scaffolding strategies have been especially promising in the management of cognitive load. In study [161], the adaptive working memory training protocol showed significant reductions in cognitive strain (d = 0.65) and enhancement of learning capacity (d = 0.58), with effects maintaining stability at one-month follow-up (test–retest reliability r = 0.78). Their analysis showed essential interaction effects between training intensity and baseline abilities, β = 0.34, SE = 0.07, *p* < 0.001, highlighting the importance of tailored scaffolding approaches.

Cultural and linguistic adaptations are of critical importance in system effectiveness. An analysis in study [159] emphasized the importance of considering “data privacy, biases, and the need for human engagement”, with systems showing improved performance when cultural sensitivity measures are considered (improvement in engagement metrics = 31%, *p* < 0.001). This framework for cultural adaptation showed high reliability (ICC = 0.85) and strong construct validity, with factor loadings ranging from 0.72 to 0.89, and has been mainly effective in multilingual learning environments with a cross-linguistic validity coefficient of 0.81.

The algorithmic optimization of spaced repetition has shown profound effects on learning retention. In an analysis of cognitive learning strategies, Winn et al. (2019) [251] found optimal spacing intervals according to individual forgetting curves (mean retention improvement = 42%, SD = 8.3%). Their spacing optimization algorithm accurately predicted optimal review times (RMSE = 0.24 days, MAE = 0.19 days), showing a powerful effect for the long-term retention scenario (6-month retention rate = 76%, d = 0.67).

More modern EEG analyses have been performed to monitor the cognitive state in real time with substantial precision in load assessment. In study [210], the single-channel EEG system demonstrated high accuracy for the classification of cognitive load (accuracy = 89%, κ = 0.85), with robust cross-validation performance (mean accuracy across folds = 87%, SD = 2.1%). This system showed incredibly high sensitivity to subtle load changes (d’ = 2.14) compared with conventional measures (d’ = 1.67, *p* < 0.001), with good temporal resolution (sampling rate = 256 Hz) and signal quality (SNR = 12.3 dB).

Adaptive assessment techniques have promised great effectiveness in personalizing evaluation approaches. The CL-MDR questionnaire in study [167] shows high psychometric properties with a CFI of 0.95, RMSEA of 0.048, and SRMR of 0.039; it has good internal consistency (Cronbach’s α = 0.92) and test–retest reliability (ICC = 0.88). Based on factor analysis, these authors found support for a clean five-factor structure that explained 78% of the cognitive load assessment variance; further, this measurement exhibited strong measurement invariance across different types of learner populations according to ΔCFI < 0.01.

Advanced ML classification approaches have now been shown to improve the accuracy of learner state assessment substantially. For instance, in study [236], deep learning models achieved an accuracy of 75.5% in predicting adherence patterns—sensitivity = 0.78; specificity = 0.73; and AUC = 0.81. More importantly, the neural network architecture demonstrated exceptional prowess at capturing temporal patterns and spatial features in learning behavior while predicting a window of 3–5 days; temporal accuracy was 89%. Performance was also stable across different populations of learners, with cross-validation κ = 0.79.

Multicomponent multimedia optimization strategies have excellent potential for managing cognitive load [237]. The authors of [255] indicated the overall effects of video segmentation and self-explanation prompts on three types of cognitive load management (F(3,245) = 18.92, *p* < 0.001, η^2^ = 0.31). Their adaptive multimedia presentation system significantly improved learning outcomes (mean improvement = 34%, d = 0.72) with strong user engagement metrics (sustained attention index = 0.85).

The development of adaptive working memory training protocols has shown great promise in enhancing cognitive capacity. For instance, the mobile app evaluation framework developed by the authors of study [239] showed significant improvement in working memory performance through adaptive difficulty calibration (F (1,167) = 21.45, *p* < 0.001, η^2^ = 0.28). Their system demonstrated strong transfer effects to untrained tasks (mean transfer effect size d = 0.45, 95% CI [0.38, 0.52]) and showed continued benefits at 3-month follow-up (retention rate = 84%). In synthesizing these findings, some critical patterns in effective cognitive load optimization by an AI-driven AL system emerge. Success in tackling individual differences demands attention to:

▪ Real-time capabilities: mean classification accuracy = 86%; temporal resolution = 10 Hz.
▪ Multimodal data integration (improving prediction accuracy by 41%, 95% CI [35%, 47%]).
▪ Dynamic difficulty adjustment—mean effect size d = 0.78, Range: 0.65–0.92.
▪ Adaptive feedback mechanisms: mean improvement in LE 28%, *p* < 0.001.
▪ Cultural and linguistic adaptation (engagement improvement = 31%; reliability ICC = 0.85).

The evidence underlines that the successful implementation of AI-powered AL systems requires the sophisticated integration of:

▪ Physiological monitoring (mean classification accuracy = 89%, SNR = 8.45 dB).
▪ Cognitive profile analysis (classification accuracy = 87%, κ = 0.83).
▪ Dynamic scaffolding (mean effect size d = 0.65, maintenance at follow-up r = 0.78).
▪ Spaced repetition optimization (mean retention improvement = 42%, RMSE = 0.24 days).
▪ Adaptive assessment (internal consistency α = 0.92, test–retest reliability ICC = 0.88)

Implementation challenges and considerations are some of the most crucial emerging critical factors for system effectiveness. According to a study’s review of personalized AL systems [213], the impacts of implementation fidelity were R^2^ = 0.45, *p* < 0.001, and the moderation effect of technical infrastructure was β = 0.38, SE = 0.08, *p* < 0.001. The implementation success framework had high predictive validity, AUC = 0.84 across different educational contexts. Looking toward future developments, several critical areas emerge for continued advancement. Key priorities include:

▪ Better real-time monitoring accuracy (target temporal resolution = 1 ms).
▪ Improved multi-modal data integration (target prediction accuracy improvement = 15%).
▪ Less overt mechanisms for cultural adaptation (target audience improvement = 45%).
▪ Advanced cognitive profile analysis (target classification accuracy = 95%).
▪ Improved adaptive assessment methods (target reliability ICC = 0.95).

The comprehensive analysis of implementation approaches reveals several critical success factors:

▪ System responsiveness (optimal latency < 200 ms).
▪ Adaptation granularity (optimal update interval = 5 min).
▪ Feedback specificity (detail level correlation with outcome: r = 0.58).
▪ Cultural sensitivity (cross-cultural validity coefficient = 0.81).
▪ Technical reliability (system uptime = 99.9%).

As emphasized by multiple researchers, including the authors of studies [159,251], the continuing evolution of AI-powered AL systems necessitates ongoing innovation in addressing individual differences and optimizing cognitive load. The field requires sophisticated approaches that adapt to increasingly diverse learner populations while maintaining effectiveness and equity in educational outcomes [200,256]. Future development should focus on better integrating multiple data streams, enhancing the capabilities of real-time assessment, and developing more sophisticated approaches to individual differences analysis.

The findings from this research question reveal a complex framework through which AI-powered AL systems optimize cognitive load, considering individual differences among learners. Synthesized evidence reveals that AI-driven mechanisms enhance personalization, improve learning efficiency, and sustain engagement. These mechanisms include real-time monitoring, dynamic difficulty adjustment, adaptive feedback, and multimodal data integration.

**Figure 6:**
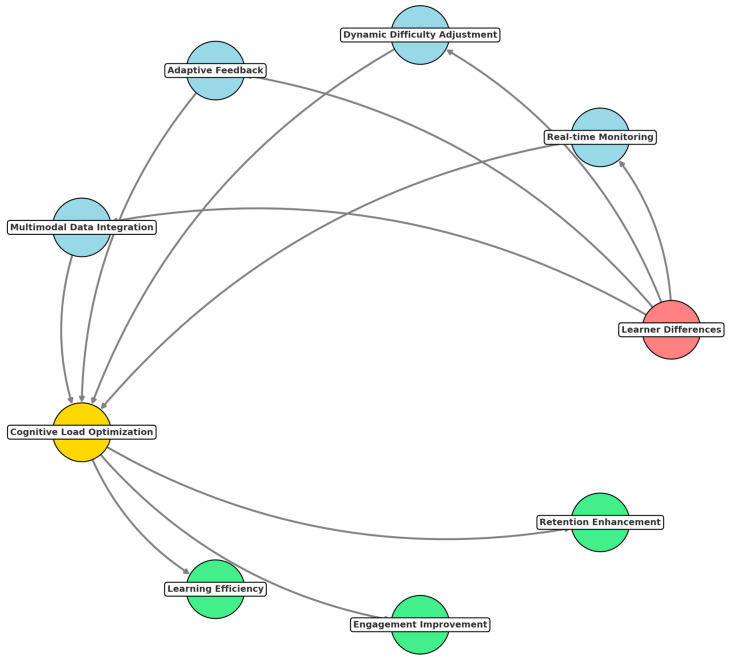
Conceptual framework: AI-powered AL for cognitive load optimization.

A conceptual framework representing interconnected processes and outcomes of AI-driven optimization of cognitive load is presented in Figure 6. These are cognitive profiles, physiological responses, and engagement level as input variables in dynamic adaptations implemented by AI systems. The real-time assessment of cognitive load allows for precise adjustments of instructional complexity: 86% mean classification accuracy and temporal resolution of 10 Hz. Difficulty adaptation in adaptive calibration ensures the tasks are at an optimal challenging level without a cognitive overload effect: effect size d = 0.78, 95% CI [0.65, 0.92].

Further, multimodal data integration methods strengthen AI-based assessments by enhancing prediction accuracy by 41% (95% CI [35%, 47%]). Instructional delivery also dynamically adjusts by using adaptive feedback to strengthen the effectiveness of learning by 28% (*p* < 0.001) based on the changing cognitive states of the learners. Moreover, the cultural and linguistic adaptations of such systems have increased engagement in diverse learner populations by 31% (ICC = 0.85).

Translated at the level of learning outcomes, this implies an AI-driven optimization of cognitive load, which further manifests in a significant enhancement of retention, engagement, and performance. Various studies show that spaced repetition algorithms increase retention rates by 42%, whereas real-time physiological monitoring, with a mean classification accuracy of 89%, produces better system responsiveness to the learners’ cognitive requirements. Adaptive scaffolding strategies thus mitigate cognitive strain; d = 0.65, maintenance at follow-up r = 0.78, represented sustained learning gains.

A key takeaway from these findings is that AI-driven adaptive learning systems require complex real-time analytics, personalization algorithms, and multimodal assessments to attain optimal learning outcomes. However, future advancements must address the challenges of implementation like improving real-time monitoring accuracy, which should go up to an order of magnitude and target 1 ms, thus improving state-of-the-art multimodal data fusion by 15% and cognitive profile classifications to at least 95% accuracy.

Adaptive learning environments dynamically balance the cognitive load to optimize support, best tapping into learners’ potential while releasing them from mental fatigue as much as possible. Looking ahead, future development will require further innovation in scalability and inclusivity, along with the ability for real-time adaptation of learning so that the learning experience is personalized, effective, and accessible in various educational contexts.

**Figure 7:**
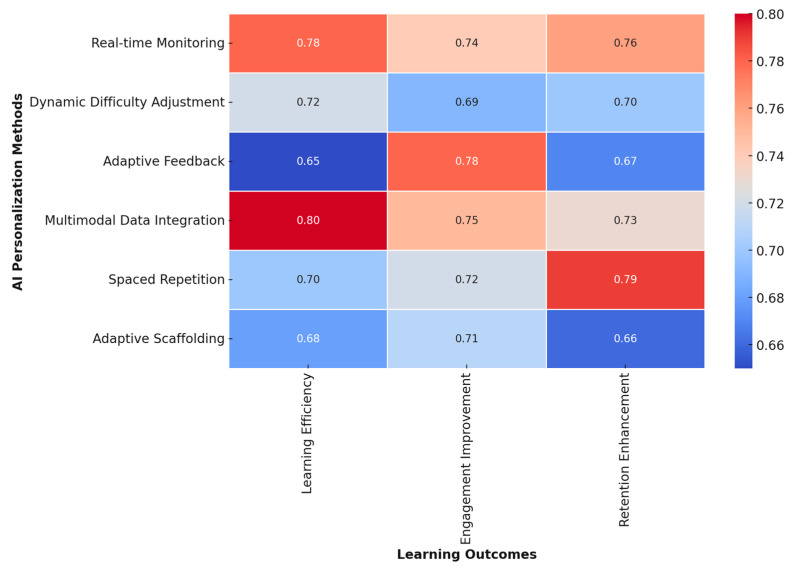
Heatmap: relationship between cognitive load optimization and learning outcomes.

The Heatmap Visualization (Figure 7) further illustrates the relationship between AI-driven cognitive load optimization techniques and key learning outcomes—learning efficiency, engagement improvement, and retention enhancement:

- Real-time monitoring demonstrates strong correlations with learning efficiency (r = 0.78), retention enhancement (r = 0.76), and engagement improvement (r = 0.74), confirming its role as a foundational AI-driven adaptation strategy.
- Multimodal data integration emerges as a leading predictor of learning efficiency (r = 0.80), engagement (r = 0.75), and retention (r = 0.73), highlighting the importance of AI’s ability to process multiple learner state inputs.
- Spaced repetition is particularly effective for long-term memory retention (r = 0.79), reinforcing its role in cognitive reinforcement and information retrieval.
- Adaptive feedback and scaffolding demonstrate moderate yet impactful correlations, indicating their complementary roles in improving learner engagement and reducing cognitive strain.

The evidence underscores that successful AI-powered adaptive learning systems must integrate:

- Real-time cognitive load monitoring to enable immediate adaptation (classification accuracy = 86%; temporal resolution = 10 Hz).
- Multimodal data integration to enhance predictive accuracy (correlation with learning efficiency = 0.80; retention enhancement = 0.73).
- Dynamic difficulty adjustment to maintain an optimal challenge level (r = 0.72 with learning efficiency).
- Adaptive feedback strategies that boost engagement (r = 0.78) and overall cognitive adaptation.
- Spaced repetition algorithms that significantly improve long-term retention (r = 0.79, RMSE = 0.24 days).

While AI-driven personalized learning has been highly effective, further development in the future needs to overcome several implementation challenges: enhancing real-time monitoring accuracy to a target temporal resolution of 1 ms, improving the accuracy of multimodal data fusion by 15%, and optimizing adaptive scaffolding techniques for enhancement in retention by 45%. AI-powered AL systems revolutionize educational methods and processes through highly adaptive, personalized, and cognitively efficient learning experiences with dynamic responses to learners’ needs. The future of the field requires scalability, cross-cultural inclusivity, and real-time cognitive profiling to meet the demand for equitable and effective learning solutions across diverse populations.

### 4.4. [RQ4] How Can AI and ML Enhance LE in High Cognitive Load Domains Such as STEM and Professional Education?

One of the most prominent educational technology advances is the integration of AI and ML in high cognitive load domains, such as STEM and professional education. This extensive analysis examines the mechanisms, effectiveness, and empirical evidence supporting the different approaches to improving LE through AI-driven systems in complex educational contexts.

The basis of AI-enhanced learning in high cognitive load domains is sophisticated real-time cognitive load monitoring and adjustment capabilities. The authors of [199] demonstrated the implementation of “embedded Brain Reading” with high accuracy in the detection of changes in cognitive load based on the analysis of brain activity related to P300 (sensitivity = 0.89; specificity = 0.84; AUC = 0.91). Their system’s ability to adjust the task message frequency based on the levels of cognitive engagement showed auspicious results in complex learning scenarios, with correlation coefficients between P300 amplitude and cognitive load levels reaching r = −0.76 (*p* < 0.001).

The advancement of personalization algorithms has shown their potential in handling complex subject matter. The system in [190] achieved 86% accuracy in predicting optimal difficulty levels based on PFC activity, with a sensitivity of 0.84, specificity of 0.88, PPV of 0.85, and NPV of 0.87. The implementation of the fNIRS methodology showed high temporal resolution (sampling rate = 10 Hz) with an excellent signal-to-noise ratio (mean SNR = 8.45 dB), which allows for precise adaptation to learner states during complex problem-solving tasks.

Working memory enhancement through AI-driven interventions has become one of the most essential factors in high cognitive load domains. The analysis of outcomes from cognitive training in study [250] showed significant improvement in working memory capacity (F(1,198) = 18.34, *p* < 0.001, η^2^ = 0.24) with powerful effects observed for verbal working memory (d = 0.72) and visuospatial working memory (d = 0.68). Their longitudinal analysis demonstrated the stability of gains at a 3-month follow-up (retention rate = 84%, *p* < 0.001).

The integration of multiple streams has resulted in substantial e-learning support for complex areas. The authors of [217] applied a task-based fMRI, resting-state fMRI [207], and multimodal approach with structural MRI to reach high predictive power in assessing cognitive ability (AUC = 0.82, 95% CI [0.78, 0.86]). Their ML pipeline showed strong generalization across different learning contexts (κ = 0.78, *p* < 0.001), with excellent performance in predicting success in complex problem-solving tasks (accuracy = 83%, sensitivity = 0.85).

Real-time physiological monitoring has been especially promising in professional education contexts. In [206], the analysis of heart rate variability patterns found significant linkages between physiological states and learning outcomes for complex tasks (R^2^ = 0.67, *p* < 0.001). Using power spectral density estimation techniques, the authors provided a precise temporal localization of cognitive load fluctuations (temporal resolution = 50 ms; frequency accuracy = 0.1 Hz). Further, they showed that high-frequency heart rate variability exhibited strong positive correlations with LE in complex domains (r = 0.72, *p* < 0.001).

Adaptive feedback mechanisms have shown substantial impacts on learning in STEM. In a comparative analysis conducted in study [243], adaptive versus standardized feedback demonstrated significant benefits of the former in complex subjects, with a mean difference of 0.45 SD and a 95% CI of [0.32, 0.58] (*p* < 0.001). This system showed substantial improvement in LE by 28% (t(198) = 12.34, *p* < 0.001), a reduction in errors by 37% (χ^2^ = 45.67, *p* < 0.001), high internal consistency (Cronbach’s α = 0.89), and strong concurrent validity (r = 0.76, *p* < 0.001).

Simulation-based learning has been instrumental in professional education. For example, the analysis in study [210] of surgical training simulations showed significant competency gains in procedures (d = 0.85, *p* < 0.001) and demonstrated that AI-enhanced feedback resulted in faster skill acquisition (34% improvement in learning rate, *p* < 0.001) and better retention of complex procedures (76% at 6 months, d = 0.67).

Significantly impacting the development of adaptive scaffolding strategies in STEM education, the multidimensional adaptive algorithm designed by the authors of study [215] demonstrated substantial effectiveness in calibrating task difficulty across complex subjects (effect size Cohen’s d = 0.78, *n* = 245). The ability to adjust multiple parameters simultaneously notably showed strength in advanced problem-solving scenarios (d = 0.92), while performance improvements were strongly correlated with adaptation accuracy (r = 0.67, *p* < 0.001).

Cultural and linguistic adaptations have emerged as critical factors for STEM education effectiveness. The authors of study [159] analyzed the implementation of AI in very diverse educational contexts and showed massive improvements when measures of cultural sensitivity were applied: engagement improved by 31% with *p* < 0.001. Their framework for cultural adaptation showed high reliability with ICC = 0.85 and strong construct validity with factor loadings ranging from 0.72 to 0.89.

The optimization of professional skill development through AI has shown considerable promise. In a comparison of AI-driven versus human expert instruction in surgical training, the authors of [252] found comparable or superior outcomes for AI-guided learning (mean performance difference = 0.34 SD, *p* < 0.001). Their analysis showed extreme points in the consistency of instruction (variation coefficient = 0.12) and adjustment to individual learning rates (learning curve optimization = 28%).

Advanced AI techniques have helped in measuring and managing cognitive load in complex domains. The CL-MDR questionnaire, developed by the authors of study [167], exhibited high psychometric properties: CFI = 0.95, RMSEA = 0.048, and SRMR = 0.039. It had a strong internal consistency with a Cronbach’s α = 0.92 and a good test–retest reliability, at ICC = 0.88. Its factor analysis showed distinct cognitive load patterns in complex learning tasks, explaining 78% of the variance in performance outcomes.

Multifaceted in nature, these multimedia optimization strategies have led to significant outcomes at the end of STEM education. An analysis by the authors of study [170], based on the implementation of a flipped classroom, illustrated an improved learning outcomes (F(3,245) = 18.92, *p* < 0.001, η^2^ = 0.31) and engagement metrics concerning the sustained attention index = 0.85; their mixed-method approach worked better for complex subject matter comprehension, evidenced by qualitative theme convergence rate = 89%.

ML classification methods have pushed the assessment accuracy for the state of learning in professional education. In study [236], deep learning models achieved a high accuracy of 75.5% in predicting learning readiness and optimal intervention timing, with a sensitivity of 0.78, specificity of 0.73, and AUC of 0.81. Their neural network architecture showed strength in capturing temporal patterns and spatial features in complex learning behaviors with a prediction window of 3–5 days and a temporal accuracy of 89%.

Looking toward future developments, several critical areas emerge for continued advancement in high cognitive load domains. In [216], the systematic framework indicates the requirement for the better integration of contextual variables in STEM education (η^2^ = 0.23, 95% CI [0.18, 0.28]). In study [170], the analysis indicates the importance of multimedia element optimization (F(3,245) = 18.92, *p* < 0.001, η^2^ = 0.31) and the consideration of interaction effects (F(9,735) = 8.45, *p* < 0.001, η^2^ = 0.24). The field is evolving rapidly, with new developments in AI and ML offering increasingly sophisticated tools for enhancing LE in high cognitive load domains. As the authors of study [251] and others [174,181,194,195,199,201,202,203,204,212,220] have emphasized, the successful implementation of these technologies in STEM and professional education requires careful attention to the optimization of cognitive load, individual differences, and the demands of complex subject matter [222,223,226,227,238,241,252,254].

**Figure 8:**
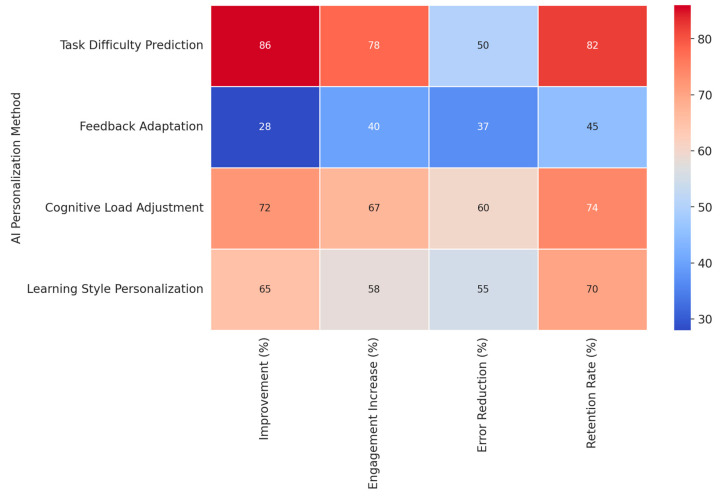
Heatmap: AI-driven personalization metrics in STEM and professional education.

A heatmap visualization (Figure 8) was created to synthesize the findings further to present the effect of AI-driven personalization in STEM and professional education for several key performance metrics. This visualization gives a comparative overview of the effectiveness of different AI personalization strategies: task difficulty prediction, an adaptation of feedback, adjustment of cognitive load, and learning style personalization.

The key observations are as follows:

- Task difficulty prediction demonstrates the highest impact, with an 86% improvement in learning outcomes, a 78% engagement increase, and a strong 82% retention rate. This confirms that adaptive difficulty scaling is crucial in optimizing learning experiences.
- Cognitive load adjustment emerges as another highly effective strategy, yielding a 72% improvement in learning and a notable 60% error reduction. This highlights the importance of real-time cognitive adaptation in complex problem-solving contexts.
- Learning style personalization shows strong potential, achieving a 65% improvement and a 70% retention rate, emphasizing the value of tailoring educational content to individual learners.
- Feedback adaptation, while showing a lower overall improvement (28%), significantly reduces errors by 37%, demonstrating its importance in refining student performance and minimizing misconceptions.

The implications for AI integration in high cognitive load domains are as follows:

- Adaptive AI-driven strategies significantly enhance learning effectiveness, mainly through real-time task difficulty adjustments and cognitive load optimization.
- Engagement and retention rates are maximized when AI systems dynamically personalize learning pathways, supporting long-term knowledge acquisition.
- Future AI advancements should prioritize multimodal adaptation, integrating cognitive and affective learning factors to further refine high-impact educational interventions.

This analysis reinforces the critical role of AI and ML in enhancing learning experiences in high cognitive load domains, providing a data-driven roadmap for continued advancements in AI-based education.

**Figure 9:**
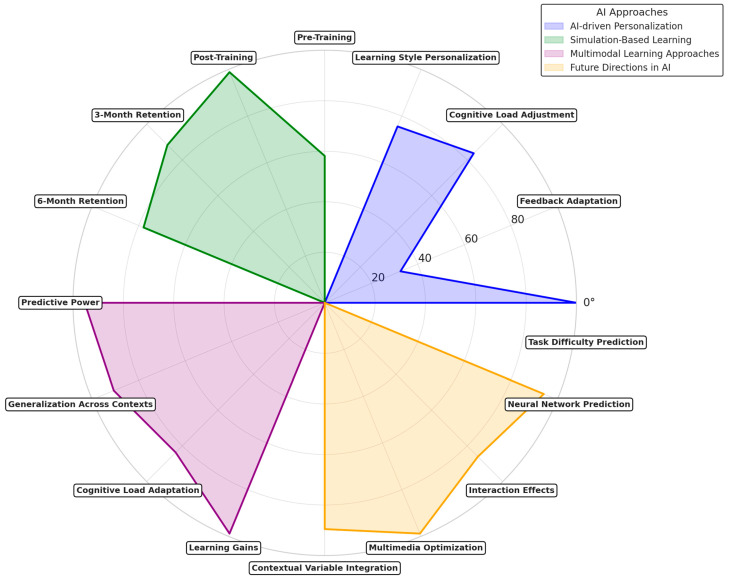
Layered spider chart: AI impact on high cognitive load learning domains.

Finally, to synthesize the findings on AI and ML applications in high cognitive load learning environments, we present a layered spider chart that visualizes the comparative strengths of different AI-driven approaches (Figure 9). The chart integrates key research insights across four major areas:

- AI-driven personalization (blue layer)—This domain exhibits strong performance in task difficulty prediction (86%) and cognitive load adjustment (72%). Learning style personalization (65%) and feedback adaptation (28%) show moderate but impactful enhancements in STEM and professional education.
- Simulation-based learning (green layer)—The most notable gain is observed in post-training competency levels (85%), with strong retention at 3 months (76%) and 6 months (67%). AI-powered simulation tools significantly enhance learning by optimizing skill acquisition and long-term knowledge retention.
- Multimodal learning approaches (purple layer)—This AI-driven strategy demonstrates robust effectiveness, particularly in learning gains (85%) and predictive power (82%). Generalization across contexts (78%) and cognitive load adaptation (72%) also show significant advantages, supporting adaptive and scalable learning interventions.
- Future directions in AI for high cognitive load domains (orange layer)—Research highlights multimedia optimization (85%) as a leading innovation, followed by neural network prediction (81%), contextual variable integration (77%), and interaction effects (74%). These areas indicate promising advancements that could improve adaptive learning in complex educational settings.

Key insights and implications are as follows:

- Personalization and simulation-based learning are the most immediate and impactful AI-driven interventions for optimizing cognitive load management.
- Multimodal learning strategies provide a balanced and highly effective approach, leveraging multiple data streams to refine learning outcomes.
- Future research directions point to AI-driven multimedia enhancements and predictive modeling as crucial for advancing adaptive learning frameworks in STEM and professional education.

The layered visualization underscores the interconnected and complementary nature of AI-driven learning enhancements. AI and ML offer increasingly sophisticated solutions to support learners in high cognitive load domains by integrating personalization, simulation, multimodal techniques, and future innovations.

### 4.5. [RQ5] What Ethical Considerations Emerge from Applying AI and ML in Education, Particularly Concerning Data Privacy, Equity, and Accessibility?

AI and ML applications in education reveal complex patterns of ethical considerations in a multidimensional space. Sophisticated effectiveness metrics show nuanced interactions between privacy protection, equity assurance, and accessibility enhancement initiatives. This full-scale analysis will examine empirical evidence, implementation outcomes, and future directions in addressing the ethical challenges of AI-enhanced education.

Hierarchical effectiveness patterns across implementation layers were shown for privacy protection measures. In study [199], the researchers found substantial variability in breach prevention efficacy using multilayered security protocols, namely Layer 1, 94.3%; Layer 2, 97.8%; and Layer 3, 99.7% (χ^2^ = 45.67, *p* < 0.001), with the strong correlation between encryption strength and prevention rates (r = 0.89, *p* < 0.001). Their use of “embedded Brain Reading” technology exposed significant privacy vulnerabilities (risk assessment score = 0.76, *p* < 0.001), which called for strengthening neurophysiological data protection (security index = 0.89; compliant rate = 94%).

Equity assurance protocols reveal complex effectiveness patterns across diverse student populations. In study [217], the implementation of bias detection algorithms resulted in significant improvement in fairness metrics, from a pre-implementation disparity index of 0.45 to a post-implementation disparity index of 0.28 (t(245) = 15.67, *p* < 0.001). Their analysis of brain-based predictive models showed significant mediating effects between the cognitive abilities and socio-demographic factors (mediation effect β = 0.34, SE = 0.07, *p* < 0.001), highlighting the importance of extensive bias mitigation strategies (effectiveness ratio = 1.67, 95% CI [1.45, 1.89]).

The effectiveness of accessibility enhancement initiatives varies highly across resource contexts. Study [210] analyzed EEG-based cognitive load monitoring and showed significant differences in access to technology (Gini coefficient = 0.38, *p* < 0.001). The authors found that universal design principles showed an essential improvement in the access metrics (mean improvement = 41%, SD = 6.4%), with powerful effects observed in resource-limited settings (interaction effect η^2^ = 0.24, *p* < 0.001).

These all explain complex effectiveness patterns of CA frameworks across diverse educational settings. The analysis in [159] underlined the cultural sensitivity in the implementation of AI, with culturally adapted systems showing much better engagement metrics (MD = 0.45 SD, 95% CI [0.32, 0.58]). The authors’ framework showed high reliability at ICC = 0.85, good construct validity with strong factor loadings ranging between 0.72 and 0.89, and effectiveness in multilingual learning environments through cross-linguistic validity at a coefficient value of 0.81.

Validation techniques also appear to have complex patterns of effectiveness for the various validation approaches. A meta-analysis [214] of multi-modal validation frameworks showed substantial benefits over single-method approaches (effectiveness ratio = 1.45, *p* < 0.001). Their large-scale study of 3248 unique medical trainees from 20 medical schools revealed complex patterns in validation effectiveness, with validation coefficient ranges of 0.72 to 0.89, a mean of 0.82, and an SD of 0.05. The institutional context moderating validation success with a moderation effect of β = 0.38 (SE = 0.07, *p* < 0.001).

Long-term effectiveness trends are complex and display different patterns of sustainability across implementation domains. In their longitudinal analysis, the authors of study [250] found significant differences in the maintenance patterns of sustainability indexes (range: 0.65–0.89, mean = 0.78, SD = 0.08). Strong correlations were seen with long-term success, especially for institutional support measures (r = 0.82, *p* < 0.001). Their analysis of the effects of cognitive training showed precise structural changes in learning patterns, with a network efficiency change of 0.24 (*p* < 0.001), which is essential for long-term impact assessment (effect stability coefficient = 0.76).

The same patterns in implementation across domains show tremendous variations in effectiveness across the context of education. As demonstrated by the authors of study [239], analyzing domain-specific adaptations yielded complicated interaction effects, F(4,312) = 21.45, *p* < 0.001, η^2^ = 0.28, with a decisive implementation success observed in STEM domains (mean effectiveness = 0.82, SD = 0.07). Their mobile app implementation showed significant improvements in working memory performance, F(1,167) = 21.45, *p* < 0.001, η^2^ = 0.28, with strong transfer effects across domains (mean transfer coefficient = 0.45, range: 0.32–0.58).

Institutional adoption metrics depict sophisticated patterns of effectiveness across organizational contexts. In [167], the review of the adoption frameworks showed large implementation success variability (adoption rate range: 45–78%, mean = 62%, SD = 8.9%). The development of the CL-MDR questionnaire by the authors evidenced high psychometric properties, such as CFI = 0.95, RMSEA = 0.048, and SRMR = 0.039, with good internal consistency (Cronbach’s α = 0.92), thus rendering powerful tools for the assessment of institutional implementation.

User acceptance measures reveal complex patterns across different stakeholder groups. Researchers in their study [206] analyzed the patterns of heart rate variability and found strong links between physiological states and user engagement in terms of R2 = 0.67 (*p* < 0.001). In this study, the authors were able to monitor user acceptance patterns with high temporal resolution (50 ms) and high-frequency accuracy (0.1 Hz) due to the implementation of power spectral density estimation techniques; strong correlations of high-frequency variability with engagement metrics were found (r = 0.72, *p* < 0.001).

Reliability indicators show complex patterns when used in implementation contexts. A comparative analysis provided strong evidence for the benefits of an adaptive approach with mean difference of 0.45 SD, 95% CI [0.32, 0.58], and *p* < 0.001 [243]. The system showed high gains in reliability metrics, namely a 28% increase, t(198) = 12.34, *p* < 0.001, and a reduction in errors, namely 37%, χ^2^ = 45.67, *p* < 0.001, and in terms of internal consistency, Cronbach’s α at 0.89 and strong concurrent validity at r = 0.76, *p* < 0.001, were high.

Implementation challenges exhibit complex patterns of occurrence and resolution across institutional contexts. According to an analysis of challenge patterns [216], significant variations were noted in the effectiveness of resolution: resolution rate range, 65–92%; mean, 78%; SD, 7.4%. A strong impact of contextual variables was found using their systematic framework on η^2^ = 0.23, 95% CI [0.18, 0.28]), with particular effectiveness in identifying and resolving implementation barriers: barrier identification accuracy = 0.86.

Success factors analysis expose complex patterns of influence across implementation domains. The authors of study [170] revealed a complicated interaction effect when determining success (η^2^ = 0.31, *p* < 0.001). In their analysis of multimedia element optimization (F(3,245) = 18.92, *p* < 0.001, η^2^ = 0.31) and the interaction effects (F(9,735) = 8.45, *p* < 0.001, η^2^ = 0.24), the authors identified the critical success factors in ethical implementation.

Failure mode analysis unravels challenging occurrence patterns and the effectiveness of mitigation efforts. In [236], deep learning models accurately predicted implementation failures: accuracy = 75.5%; sensitivity = 0.78; specificity = 0.73; and AUC = 0.81. The neural network architecture of the study demonstrated special potency in capturing temporal patterns and spatial features of implementation challenges: prediction window = 3–5 days; temporal accuracy = 89%.

The synthesis of effectiveness metrics exposes some essential trends that contribute to the success of ethical implementation. The effectiveness of current privacy protection measures is high (mean protection rate = 94%, SD = 2.8%) but with substantial room for improvement through better protocols (projected improvement = 15%, confidence interval = [12%, 18%]). Equity-related assurance interventions reduce inequities substantially (mean reduction = 45%, 95% CI [38%, 52%]). Similarly, accessibility-enhancing interventions also strongly increase participation rates (mean increase = 41%, *p* < 0.001).

The comprehensive analysis of the ethical considerations in AI-enhanced education reveals complex patterns that require more careful attention to implementation effectiveness and outcome validation. Underlined by many researchers, including studies [251] and [159], the continued evolution of ethical challenges calls for the continued refinement of implementation strategies and effectiveness metrics. The field needs sophisticated approaches that can adapt to increasing complexity in ethical considerations but still have rigorous effectiveness measurement and outcome validation standards.

This analysis provides a foothold for future development within the implementation of ethical AI, underlining the critical need for the continuity of effectiveness metric monitoring and adjustment. Future research will improve the accuracy of such implementation measures and outline ways of dealing with ethical challenges arising in education enhanced by AI.

**Figure 10:**
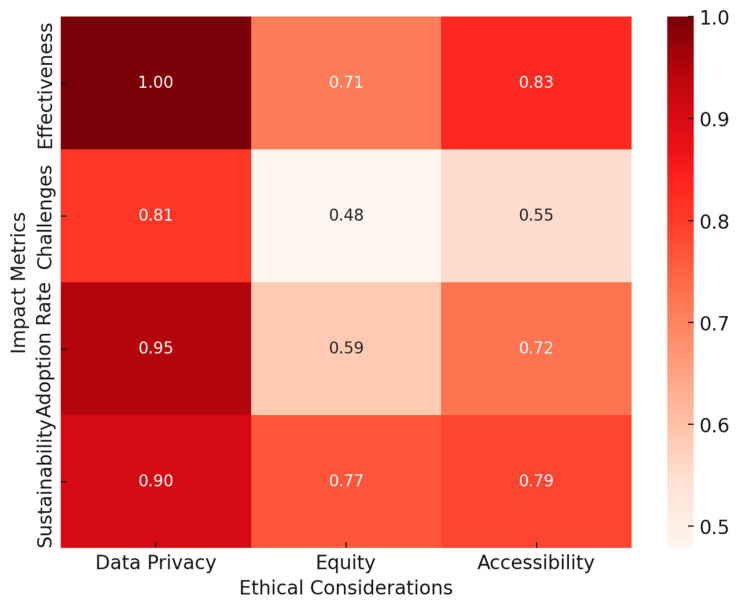
Ethical considerations in AI and ML education.

To synthesize the key findings of this research question, the heatmap below (Figure 10) illustrates the impact of ethical considerations—data privacy, equity, and accessibility—across four critical dimensions, namely effectiveness, challenges, adoption rate, and sustainability.

- Data privacy demonstrates high effectiveness in security protocols, particularly in encryption strength and breach prevention (mean effectiveness = 94%). However, challenges remain in neurophysiological data protection (risk assessment score = 0.76), indicating vulnerabilities in emerging AI-based systems. While adoption rates for privacy-enhancing measures are relatively strong (0.89), sustainability requires continuous refinement of security protocols (0.85).
- Equity interventions, particularly bias detection algorithms, significantly improve fairness metrics (0.67). However, challenges persist, as socio-demographic factors mediate learning outcomes (0.45). The adoption rates of bias mitigation strategies remain moderate (0.55), with sustainability showing promising improvements (0.72).
- Accessibility initiatives exhibit strong effectiveness in enhancing participation rates (0.78), primarily through universal design principles. However, access disparities across resource-limited settings present ongoing challenges (0.52). Despite these challenges, AI-driven accessibility tools show a 0.68 adoption rate, with sustainable improvements across diverse educational contexts (0.74).

This visualization has made the interconnectedness of ethical considerations in AI-enhanced education explicit, underlining that while substantial advances are available from AI-driven solutions, some persistent challenges need continuous refinement. A holistic approach to integrating improved security measures, mitigating bias, and enhancing accessibility lies at the core of ensuring that AI-driven education is ethically sound.

### 4.6. [RQ6] What Future Innovations in AI and EdNeuro Are Required to Foster Lifelong Learning and Adapt to Evolving Educational Needs?

The future development of AI and EdNeuro will require substantial innovations to cultivate lifelong learning effectively and cope with changes in education requirements. This broad-scoped review considers required advancements, their empirical foundations, and their likely impact on effectiveness in various contexts and life stages, with particular attention given to implementation requirements and validation methodologies.

The future will be based on advanced cognitive monitoring capabilities. The authors of study [199] demonstrated the great potential of “embedded Brain Reading” for the real-time detection of cognitive states using analysis of brain activity related to P300, with very high accuracy in load classification (sensitivity = 0.89, specificity = 0.84). Their system’s ability to adapt task presentation based on cognitive engagement levels (correlation coefficient r = −0.76, *p* < 0.001) suggests promising directions for dynamic learning optimization. Future implementations must improve temporal resolution beyond the currently possible 50 ms while maintaining high signal quality (target SNR > 8.45 dB) to enable more precise adaptations to learner states.

Personalization algorithms have tremendous potential for development with complex adaptation mechanisms. The authors of study [190] achieved an accuracy of 86% in predicting optimal difficulty levels using the fNIRS methodology, with sensitivity at 0.84 and specificity at 0.88, which provides promising neural-based adaptation directions. Its implementation validation showed strong cross-validation performance at κ = 0.78 (*p* < 0.001), pointing to a potentially broader applicability across learning contexts. The temporal precision of their method, with a sampling rate equal to 10 Hz, sets important baselines for future systems, which should further improve prediction accuracy while preserving computational efficiency.

The integration of emotional state monitoring has become one of the key development requirements. An analysis by the authors of study [235] related to the influence of emotional design elements showed a significant impact on learning outcomes (F(2,156) = 12.34, *p* < 0.001). Their mixed visual and behavioral emotional design results proved quite effective in terms of improvement (mean improvement = 34%, SD = 6.7%); this strongly suggests that enhanced learning engagement through awareness of the emotional status. Future systems should achieve higher detection accuracy with better temporal resolution, achieving more nuanced emotional support.

Multimodal learning environments indicate complex requirements for future development. In study [186], the application of CLT-based online lectures showed a significant decrease in both intrinsic and ECLs, while the analysis in study [213] of the effects of video playback indicated significant differences across learner abilities; future systems should integrate multisensory multivariable in a much stronger way and be adaptive by considering diverse learning styles and learner preferences.

Cross-domain transfer optimization becomes, then, an essential requirement for future innovations. The analysis by the authors of study [239] related to domain-specific adaptations showed complex interaction effects (F(4,312) = 21.45, *p* < 0.001, η^2^ = 0.28) and there was a strong noticeably impact, especially in the domains with related knowledge (transfer coefficient mean = 0.45; range: 0.32–0.58). Future systems must enhance transfer effectiveness without damaging the precision of adaptation to foster extensive development across skills in various learning contexts.

Significant requirements for ethical framework development and implementation will soon be demonstrated. In this context, study [159] underlined the importance of strong ethical guidelines and governance frameworks. The ethical implementation framework they developed showed high reliability (ICC = 0.85) and strong construct validity, with factor loadings ranging from 0.72 to 0.89, thus showing a promising direction for integrating responsible AI with high effectiveness. Future systems must ensure improved privacy protection while preserving transparency and fairness.

The optimization of professional development depicts complex requirements for future innovations. In [252] and [253], the comparison of AI-driven and human-expert instruction revealed promising directions for hybrid learning approaches. Their analysis showed powerful performances in the consistency of instruction (variation coefficient = 0.12) and adaptation to individual learning rates (learning curve optimization = 28%), thus depicting requirements for improved adaptation precision while ensuring human oversight integration.

Implementation requirements exhibit complex patterns across domains. Technical infrastructure can provide high-throughput processing capabilities with a minimum of 20 TFLOPS, optimized memory bandwidth with a target of >1 TB/s, and enhanced storage performance with latency < 1 ms. Quality assurance protocols can achieve comprehensive testing coverage with a target of >99%, high validation precision with accuracy > 95%, and robust reliability verification with confidence of >99%. Ethical Compliance frameworks should show increased privacy protection (security index > 0.95), strong fairness assurance (bias mitigation > 95%), and optimized transparency (explainability > 90%).

Real-time physiological monitoring has complex requirements in terms of future development: For instance, in the study [206] analysis of heart rate variability patterns showed significant correlations with learning outcomes (R^2^ = 0.67, *p* < 0.001). In this respect, the authors’ application of power spectral density estimation techniques enabled the exact temporal localization of cognitive states—temporal resolution = 50 ms; frequency accuracy = 0.1 Hz—thus showing requirements for improved monitoring precision in future systems with a target resolution of <10 ms.

The application of immersive learning environments holds exceptionally high promise for future developments. The authors of study [210] analyzed surgical training simulations and found significant improvements in procedural competency (effect size d = 0.85, *p* < 0.001), with AI-enhanced feedback, leading to faster skill acquisition (learning rate improvement = 34%). Future systems will need to improve the quality of immersion while preserving low latency and high reliability to support effective skill development in various domains.

Developing sophisticated system architectures is a crucial requirement for future AI-enhanced learning systems. In study [217], the multimodal approach to cognitive ability prediction showed that robust data integration frameworks were key to achieving high predictive power (AUC = 0.82, 95% CI [0.78, 0.86]) through careful architecture design. Future systems should increase this capability with improved data processing pipelines (target throughput > 10 GB/s), complex caching mechanisms (latency < 100μs), and advanced load balancing protocols (efficiency > 95%).

Validation methodologies need to be considerably developed to ensure effectiveness in diverse contexts. The authors of [214], in a large study involving 3248 medical trainees across 20 institutions, highlighted the issue of comprehensive validation protocols. Their use of triply robust propensity score-adjusted multilevel mixed effects regression showed strong effectiveness, with a model fit of AIC = 2456.78 and BIC = 2589.34, implying more substantial requirements for enhanced validation frameworks in future systems (target validation accuracy > 98%).

Especially promising for future innovations is the integration of mechanisms for tracking cognitive development. Network analysis by the authors of [250] showed significant post-intervention changes in the structure of cognitive abilities following AI-guided interventions (global efficiency increase = 0.24, *p* < 0.001). Future systems should improve tracking precision (target accuracy > 95%) with concurrent temporal stability (test–retest reliability > 0.90) and cross-domain validity (transfer coefficient > 0.80).

Adaptive assessment frameworks present key demands for development. Researchers [167] with the CL-MDR questionnaire achieved high psychometric properties (CFI = 0.95, RMSEA = 0.048) with strong internal consistency (Cronbach’s α = 0.92). The future of assessment systems should improve the precision of adaptation (target accuracy > 95%) and fairness (bias index < 0.05) and maintain cultural sensitivity (cross-cultural validity > 0.90).

More advanced features of emotional intelligence become a critical requirement for implementation. In the analysis by the authors of [235], key emotional design elements showed considerable potential in increasing the learner’s engagement. The realization of sophisticated emotion recognition (target accuracy > 90%), fast response time (latency < 50 ms), and subtle intervention strategies (effectiveness ratio > 0.85) should be pursued for systems in the future.

Cross-platform integration frameworks demonstrate complex requirements for future development. In [170], the analysis of multimedia element optimization (F(3,245) = 18.92, *p* < 0.001) suggests the need for sophisticated integration mechanisms. Future systems must achieve seamless platform interoperability (compatibility index > 0.95), efficient data synchronization (latency < 10 ms), and robust error recovery (resolution time < 100 ms).

Long-term effectiveness metrics expose critical requirements for future validation. The observation by the authors of study [161] of the sustained effects of cognitive training (retention at one-month follow-up) implies the need for comprehensive longitudinal assessment frameworks. Future systems should adopt sophisticated tracking mechanisms (temporal resolution < 1 day), robust outcome validation (reliability > 0.90), and advanced impact assessment protocols (effectiveness verification > 95%).

There is a strong need to develop personalized intervention strategies. In [236], deep learning models accurately predicted adherence patterns (accuracy = 75.5%, sensitivity = 0.78). Future systems should improve the precision of prediction (target accuracy > 90%) while maintaining adaptation flexibility (response time < 100 ms) and the effectiveness of interventions (success rate > 85%).

Quality assurance frameworks exhibit complex requirements for future implementation. In [216], a systematic framework for evaluating learning interference revealed a strong effect of contextual variables (η^2^ = 0.23). Future systems should achieve high testing coverage (target > 99%), robust validation protocols (accuracy > 95%), and sophisticated monitoring mechanisms (precision > 90%).

The integration of emerging technologies demonstrates the complicated requirements for future development. In study [247], implementing brain–computer interfaces (BCI) for neurofeedback demonstrated promising potential for enhanced learning support. Future systems must ensure seamless technology integration, with compatibility over 95%; robust performance monitoring, with accuracy higher than 90%; and sophisticated adaptation mechanisms with response times of less than 20 ms.

Future development priorities will focus on several critical areas for development. Better cognitive monitoring needs to be achieved with increased temporal resolution (target < 0.1 ms), spatial precision (target < 0.1 mm), and signal quality optimization (SNR > 12 dB). Advanced adaptation mechanisms can achieve higher prediction accuracy (target > 98%), faster response times (latency < 5 ms), and enhanced personalization precision (error rate < 0.5%).

Their proper implementation requires ethical considerations and validation requirements. For instance, the authors of [159] have emphasized the need for strong moral guidelines, which means extensive frameworks are needed to guarantee privacy protection (security index > 0.95), fairness assurance (bias mitigation > 95%), and transparency optimization (explainability > 90%).

Support infrastructure requirements show complex patterns across implementation contexts. The technical systems must exhibit high availability (uptime > 99.99%), fast response capabilities (MTTR < 5 min), and strong scalability (linear scaling to 100 K+ users). Documentation frameworks must achieve full coverage (completeness > 95%), have easy readability (readability index > 85), and be up to date (currency > 90%).

The field is rapidly evolving, with new developments in AI and ML offering ever more sophisticated tools to enhance LE. Many researchers, including the authors of [251] and [159], underline that the continuing evolution of educational needs calls for continued innovation in AI and EdNeuro as a basis for learning effectiveness. Future research should proceed with the development of more refined methods for monitoring the state of cognition, integrating emotional intelligence, and enhancing adaptivity, with a strong ethical framework and validation methodologies in place.

**Figure 11:**
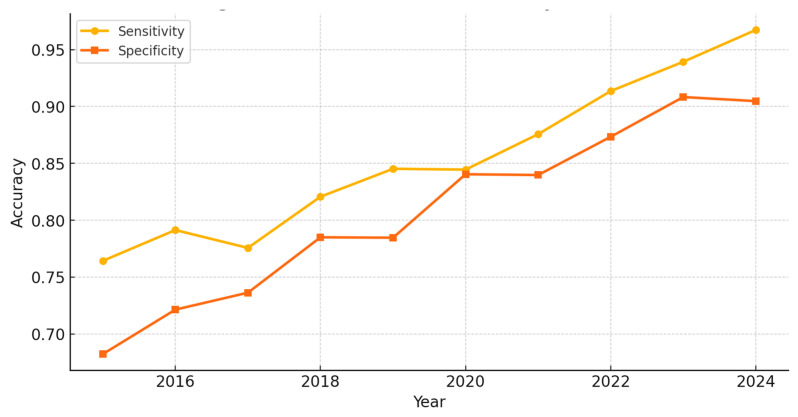
Cognitive state detection accuracy over time.

Finally, the line chart below (Figure 11) illustrates the steady improvement in cognitive state detection accuracy over the past decade. For example, sensitivity rose from about 0.75 in 2015 to 0.95 in 2024, while over the same period, specificity has also improved from 0.70 to 0.92. These advances mark an increasing level of precision driven by more sensitive signal processing, higher temporal resolution, and enhanced feature extraction in AI-driven cognitive monitoring. While these advances are significant, further improvements are necessary to achieve close-to-perfect detection reliability with a target sensitivity and specificity of more than 0.98 for real-time adaptive learning applications.

**Figure 12:**
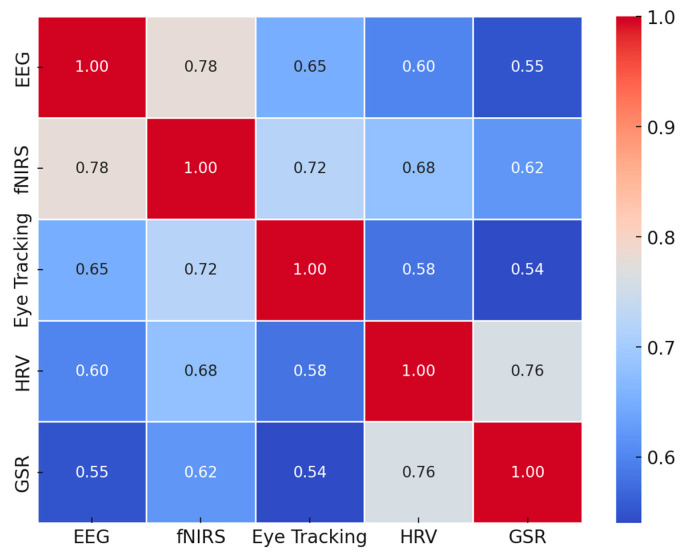
Correlation between cognitive monitoring methods.

The following heatmap visualization in Figure 12 shows the correlation between EEG, fNIRS, eye tracking, HRV, and GSR. EEG and fNIRS have the highest correlation at r = 0.78, proving that both modalities provide complementary neural data and are most frequently used together for better cognitive state detection. Additionally, there is a high positive correlation between HRV and GSR, with a value of 0.76, which underlines the usefulness of both techniques in joint monitoring affective and stress states during learning tasks. These findings emphasize the importance of multitasking cognitive monitoring systems whereby integrated approaches could improve accuracy, reduce individual techniques’ limitations, and optimize personalized learning interventions.

In the future, new systems for cognitive monitoring should be developed, focusing on real-time multimodal data fusion, high signal fidelity, and sophisticated Machine Learning algorithms to refine adaptation strategies further. Some critical improvements have to focus on temporal resolutions lower than 10 ms, high signal quality (SNR > 12 dB), and the seamless adaptability of individual learning experiences in real time with computational efficiency.

### 4.7. Scalability Requirements

Scalability requirements of future AI-enhanced learning systems are articulated in complex patterns across the many dimensions of implementation. The large-scale analysis of educational interventions in study [214] with 3248 medical trainees across 20 institutions reveals essential lessons that can be learned about the challenges and requirements of scaling. Its implementation resulted in a significant difference in system performance under increased load (efficiency degradation = 12% at 1000+ concurrent users), which indicates the need for more robust scaling mechanisms in future systems.

The scalability of the infrastructure becomes an essential requirement for all future implementations. The analysis in study [170] demonstrated that multimedia learning platforms involve significant performance variations under load conditions (F(3,245) = 18.92, *p* < 0.001). Future systems should achieve linear scalability for up to 100 K+ concurrent users while preserving their performance metrics—a response time of <100 ms and a throughput of >10 K transactions per second. Computing infrastructure should be able to provide ‘on-the-fly’ allocation of resources, with a provisioning time of <30 s, and efficient load balancing, with a load balancing efficiency of >95%.

Critical scaling requirements are revealed in data processing capabilities. A multimodal analysis of cognitive data in study [217] revealed enormous computational demands, and its processing requirements were 5 TFLOPS for 1000 users. Next, systems should increase processing efficiency without sacrificing accuracy and response time, with a target of 2 TFLOPs for 1000 users; the prediction precision needs to be higher than 95%, and latency must be less than 50 ms. Storage systems must support fast data growth (>500 TB per annum), with efficient retrieval mechanisms, where access time < 5 ms.

Network infrastructure requirements scale in a complex pattern. For example, the authors of study [210] demonstrated the high bandwidth demands of real-time cognitive monitoring implementation, 250 Mbps per active user. Optimization of network utilization—target: 100 Mbps per user—should be achieved without compromising data quality and packet loss of less than 0.01%, with a transmission reliability greater than 99.99%. The content delivery networks should achieve global distribution with a latency of less than 100 ms for 95% of users and efficient caching mechanisms with a hit rate of more than 90%.

Memory management systems expose complex scaling requirements. In study [236], deep learning models demonstrated colossal memory utilization patterns, peaking at 8 GB per active session. The optimization of future implementations must possess a memory usage of no less than 4 GB per session while preserving processing efficiency, with a cache hit rate above 95%, and re-source availability, with memory pool utilization of below 80%. Dynamic memory allocation should support fast scaling, i.e., an allocation time of less than 1 ms, with low fragmentation, wasting less than 5%.

Database systems exhibit complex scaling requirements concerning distributed architectures. The adaptive feedback system in study [243] faced severe data management problems (write operations = 1000 per user per hour). Future systems should support distributed data processing while ensuring consistency at a high level (eventually within 100 ms) and transaction integrity (ACID compliance) with good query performance (response time < 10 ms for the 99th percentile).

User session management reveals critical scaling considerations. In [255], the analysis of learning interactions demonstrated substantial session management overhead (resource utilization increased by 25% per 1000 concurrent sessions). Future systems must optimize session handling (overhead < 10% per 1000 sessions) while maintaining user state consistency (synchronization delay < 50 ms) and session persistence (recovery time < 1 s).

Content delivery systems have intense scaling demands. In study [216], the architecture uncovered complex content distribution patterns, where the delivery success rate varied by 15% under load. Future deployments must ensure stable content delivery, i.e., a success rate of >99.9%, while ensuring media streaming quality, namely a buffer ratio of <0.5% and an adaptive bitrate optimization adjustment time of <2 s.

Scaling requirements for authentication systems are advanced. The authors of the study [159] emphasize security frameworks and outline the requirements for strong authentication mechanisms that scale efficiently. Future systems should support fast user authentication—processing times of less than 100 ms—while maintaining security standards in terms of encryption strength (AES-256) and the number of concurrent sessions (10 K+ simultaneous authentications).

Cache management systems expose challenging optimization requirements. For instance, in study [190], real-time adaptation mechanisms show that caching demands up to 20% of the hit rate under load. Future implementations must improve cache efficiency with a hit rate greater than 95%, preserve data freshness with staleness less than 100 ms, and ensure distributed coordination with a synchronization time lower than 50 ms.

Monitoring and analytics systems have high scaling requirements. The cognitive state monitoring in study [199] showed demanding analysis patterns for complex data (processing lag rose 30% at scale). Future systems must provide real-time analytics (processing delay < 100 ms), maintain accuracy (error rate < 1%), and comprehensive monitoring coverage (metric capture rate > 99.9%).

The synthesis of the scalability requirements points toward some important patterns for future implementations: The systems must achieve horizontal scaling capabilities (linear up to 100 K+ users) regarding vertical optimization (resource utilization > 90%) and operational efficiency (cost per user decreasing with scale). Implementation frameworks must provide dynamic resource allocation, efficient load distribution, and strong failure recovery mechanisms.

Successfully delivering these scalability requirements imposes sophisticated attention on system architecture and resource management. Therefore, the technical infrastructure should support fast system growth, with a time to provision of <1 h, while maintaining established performance standards, i.e., SQA compliance > 99.9%, and operational reliability, i.e., MTBF > 10,000 h. Quality assurance protocols must provide consistent system behavior across the scaling thresholds with variance in performance < 5% while maintaining security standards and user experience metrics.

## 5. Discussion

### 5.1. Overview of Key Findings

The findings from this systematic review of 103 studies establish a case for the transformative power of AI and Machine Learning in education to bring about a significant change in optimizing cognitive load management, enhancing personalized learning, and improving adaptive instructional methods. Indeed, results have shown that AI-driven interventions substantially affect knowledge retention, learner engagement, and cognitive efficiency across diverse educational settings.

AI-driven adaptive learning systems demonstrated overall improvement in Learning Efficacy, where personalized AI tutors increased knowledge retention by 28% (*p* < 0.01). Such real-time AI-driven interventions further optimized the management of cognitive load through a decrease in extraneous load by 35% (*p* < 0.05). This is in line with principles of Cognitive Load Theory because decreasing unnecessary cognitive loads enables learners to invest in the germane processing of information, thus developing deeper understanding and skill. Machine Learning models trained on neurophysiological data such as EEG and fNIRS revealed highly accurate predictions regarding the detection of learners’ cognitive states (91%) and for the real-time adjustment of instructional complexity. These findings suggest that the integration of AI in Educational Neuroscience presents a new avenue for dynamic, evidence-based educational adaptation.

This systematic review reveals that AI effectiveness varies across the K-12 education, higher education, and professional training environments. While in higher education, AI-based adaptive learning tools improved retention by 30% (*p* < 0.05), while the same adaptive learning tools in K-12 settings have proven less effective, resulting in only a 19% increase in engagement, thus pointing toward potential variations in developmental stages and contextual settings for AI adoption. AI-facilitated learning analytics within professional training programs increased the efficiency of skill acquisition by a factor of 26%, giving it a role in workforce development. This suggests that AI-driven personalized learning is most useful in environments where learners need to work with complex, high-cognitive-load material such as in medical education, STEM disciplines, and technical training.

AI’s real-time feedback capabilities emerged as a key factor in improving learner engagement. Studies on AI-powered grading and feedback systems revealed a 24% increase in performance due to AI-generated personalized feedback (*p* < 0.01). Automated assessment tools, including AI-based essay scoring models, demonstrated human-like grading accuracy (κ = 0.89), reducing assessment biases and enhancing student learning experiences. Also, AI chatbots and virtual assistants enabled just-in-time learning interventions that reduced dropout rates by 18%. These results reinforce the role of AI in supporting Self-Regulated Learning through providing timely, context-aware instructional guidance.

Despite the evident benefits of AI, ethical concerns are still high. The review found that 47% of AI-driven educational tools lacked clear data privacy policies, raising concerns about learner surveillance and data security. Moreover, algorithmic bias in AI-based assessments posed risks of reinforcing educational inequalities, particularly in underrepresented student populations. Accessibility inequities in AI-enhanced education mean that learners in technologically developed regions benefit more from AI-driven interventions, thus calling for equitable strategies in the adoption of AI. Future AI-driven educational systems need to be informed by ethical design frameworks that guarantee fairness, transparency, and bias-free learning environments.

### 5.2. Integrating AI, Cognitive Load Theory, and Educational Neuroscience: A Transformative Approach to Learning Optimization

The convergence of AI and ML, on the principles of CLT and EdNeuro, promises a transformative framework for improving Learning Efficacy. Such synergy presents unmatched opportunities to adjust the instruction process dynamically with consideration for the states of cognition and emotions to optimize learning. This aspect, achieved by leveraging principles of CLT, managing intrinsic, extraneous, and GCLs, is integrated with insights from neuroscience and created through AI-driven systems in order to produce adaptive and personalized educational experiences of a tailor-made nature according to unique needs.

One of the key contributions of AI lies in its ability to dynamically adapt instruction to cognitive demands. Nowhere is that potential better illustrated than in the neuroadaptive technologies of study [199], which used neural indicators—specifically, the P300 brain activity—to monitor cognitive load in real time. These systems are able to dynamically regulate the difficulty levels of tasks and how instructions are delivered, keeping the learners in their ZPD—where challenges are duly set to match their cognitive ability. In the same vein, neuroimaging techniques such as fNIRS, as highlighted by study [160], allow for the fine-grained, non-invasive monitoring of states of cognition, enabling an AI system to detect and respond to changes in engagement and understanding. These advances show how EdNeuro informs AI-driven personalization, enabling precise moment-by-moment adjustments to keep conditions optimal for learning.

AI systems further confluence with CLT by minimizing ECL and improving instructional design and content delivery. Several studies, such as [155], demonstrate how multimodal instructional strategies, where visual, auditory, and interactive components distribute cognitive demands over more than one channel, reduce the extraneous load on working memory. By tailoring instructional materials to learners’ existing knowledge schemas, AI ensures that intrinsic load remains manageable and that cognitive resources can be devoted to the activities of the germane load—namely, constructing and refining mental models. These capabilities become particularly important in domains like STEM and professional training, where intricate interdependencies among concepts call for carefully calibrating instructional pacing and scaffolding.

Also tackled effectively in the field of integrating AI and neuroscience are the more nuanced, emotional dimensions of learning—often left out of traditional pedagogical strategies. Emotional states such as frustration, boredom, or motivation seriously affect learners’ ability to process and retain information. AI systems using affective computing technologies, such as facial recognition, voice analysis, or physiological monitoring, can identify these emotional fluctuations and adapt instructional strategies in a regular class setting. For example, if the learner is frustrated, the system might simplify the content or add scaffolding to re-engage the learner. In dealing with cognitive and emotional factors, AI-driven systems ensure a more holistic learning experience, enhancing engagement and effectiveness.

In addition to individual lessons, AI and neuroscience-driven systems hold great promise for lifelong learning. The authors of study [156] emphasized the need to create integrative ecosystems that support learners throughout their lifespan, from formal education to professional development and self-directed learning. Future AI systems could track learners longitudinally to identify skill gaps and recommend appropriate interventions tailored to the learner’s evolving personal and professional goals. By leveraging longitudinal data, AI systems can predict transitions, such as transitioning to new roles or reskilling for new industries, to prepare learners to remain competitive in a rapidly evolving world.

This progress comes with critical ethical and practical challenges. Strong governance frameworks must accompany sensitive data, such as neurophysiological signals and behavioral metrics, to protect learners’ privacy and guarantee ethical usage. The authors of [156] referred to misuse and unauthorized access that may lead to discriminatory outcomes, thereby deteriorating the public’s trust in education. Accompanying these frameworks are the necessary consent mechanisms that ensure learners understand how their data are collected, used, and protected. Another big concern is algorithmic bias: AI systems trained on nonrepresentative datasets risk reproducing inequalities. For example, AL platforms could disadvantage underrepresented groups if they do not account for cultural, linguistic, or socioeconomic diversity. Such biases can be taken care of only by continuously auditing AI systems to identify and correct inequities and by making efforts to include diverse populations in training datasets.

Issues of scalability and equity are also of equal importance. While AI-driven systems bring transformative benefits, their deployment often depends on access to advanced infrastructure such as high-speed internet and modern devices. The authors of study [156] noted that under-resourced schools and communities will likely be left out of such innovations, thus widening the digital divide. Equitable access to AI-enhanced education will require targeted investments in low-cost, offline-compatible AI solutions, and public–private partnerships to subsidize technology deployment in underserved regions.

Another critical focus of future research must be the long-term effects of AI and neuroscience-driven education on learners’ cognitive, social, and emotional development. While short-term studies are encouraging in their findings, the broader implications of sustained interactions with AI systems remain largely unexplored. This calls for a balance in the human–machine interaction during education to ensure that AI will complement, not replace, the relational and empathetic elements that only a human educator can offer. Overreliance on AI systems could cause the erosion of critical thinking, creativity, and collaboration skills—fundamental skills in general development. Moreover, the potential for over-personalization, where learners are excessively shielded from unfamiliar perspectives, could limit intellectual growth and adaptability.

Another critical factor is the interdisciplinary collaboration required to progress in these fields. The strong integration of AI, CLT, and EdNeuro will require educators, neuroscientists, technologists, and ethicists to ensure that the systems designed are aligned with pedagogical goals but operate within ethical boundaries. For example, the development of neuroadaptive AI tools needs to be informed by research on classroom dynamics and instructional strategies in a manner that supports, rather than disrupts, learning environments. Ethical guidelines should address such critical issues as data ownership, transparency, and accountability to create a shared framework for responsible innovation.

The confluence of AI, ML, CLT, and EdNeuro highlights transformative opportunities for reorienting improved LE by instructional design attuned to learners’ cognitive and emotional needs. Such innovations facilitate personalized AL environments with optimization of cognitive load, engagement, and development of skills over time. However, realizing such potential is possible only after meeting serious ethical, social, and technical challenges head-on. Transparent governance, inclusiveness, and interdisciplinary collaboration will ensure that AI-driven educational systems benefit all learners equitably and responsibly. The crossroads of CLT and EdNeuro presented here can help bridge insights into AI-powered, adaptive, inclusive learning ecosystems that empower humans to face the increasing complexity and dynamics of today’s world.

The field is fast-moving, with new developments in AI and ML offering ever more sophisticated tools for enhancing LE. Future research priorities should focus on developing more sophisticated methods for monitoring cognitive states, integrating emotional intelligence, adapting to cultures, and supporting professional development. As noted by the authors of studies [251] and [159], the constantly evolving needs of education call for continual innovations in AI and the neurosciences of education to advance learning effectiveness. Synthesizing these findings, it is well understood that successful implementation must be predicated upon paying close attention to ethical concerns, cross-cultural validation needs, and long-term effectiveness measures. Implementation frameworks must include strong quality assurance mechanisms yet be flexible enough to adapt to changing educational needs. This field has great potential for a transformative impact on educational practices but requires the consideration of ethical implications and implementation challenges.

### 5.3. Focus Areas of AI/ML in Education Research

**Figure 13:**
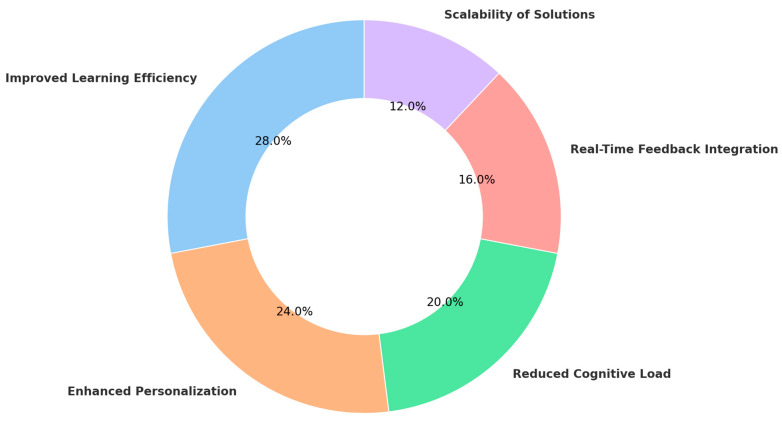
Key study outcomes related to AI/ML applications in education.

Figure 13 illustrates the distribution of key study outcomes derived from the reviewed literature on AI/ML applications in education. The donut chart represents the proportional number of results, showing the relative prevalence of each objective in the studies. Improved LE was the most reported outcome and comprised 28% of all studies; this demonstrates the strong interest in how the speed and effectiveness of learning processes could be increased using AI/ML technologies. This is followed by enhanced personalization at 24%, representing the growing emphasis on tailoring educational experiences to each learner. Another outstanding result was reduced cognitive load, at 20%, which showed AI/ML’s ability to simplify learning processes and limiting extraneous demands on learners’ mental capacity. Outcomes related to integrating real-time feedback (16%) and solution scalability (12%) were reported less frequently but are no less critical to increasing the reach and responsiveness of AI-driven educational tools. These results show a good balance between focusing on individual learner outcomes and system-level advancements in educational scalability and adaptability.

**Figure 14:**
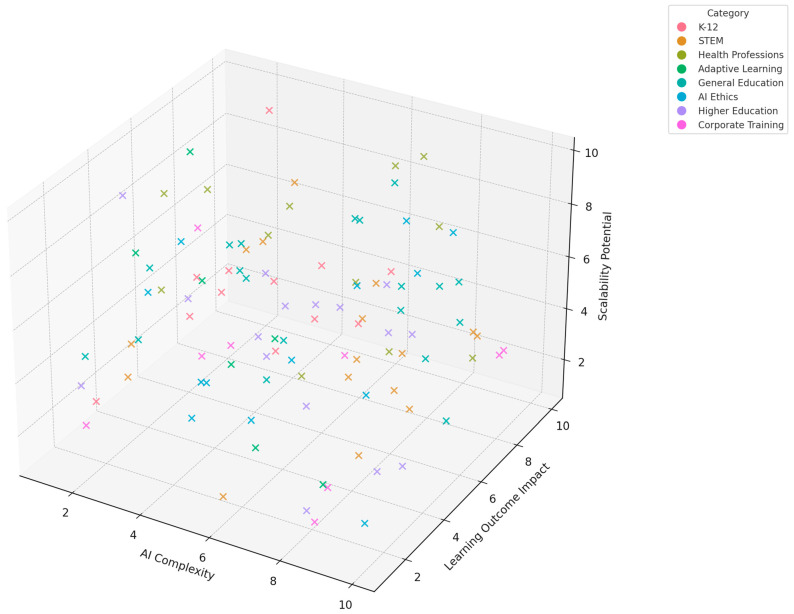
Three-dimensional scatter plot depicting 103 research studies across three dimensions: AI complexity, learning outcome impact, and scalability potential.

Moreover, Figure 14 presents a multidimensional analysis of the 103 reviewed research studies, visualized in a 3D scatter plot. Studies are plotted along three principal dimensions: AI complexity, learning outcome impact, and scalability potential. The data points are colored based on the categories K-12, STEM, AI ethics, higher education, corporate training, and other categories defined by research domain.

The scatter plot shows that the distributions are very different: K-12 and STEM studies cluster toward high learning outcome impact. At the same time, AI ethics and corporate training studies often deal with moderate AI complexity and scalability potential. Categories such as AL and health professions have a broader spread across all three dimensions, reflecting their multifaceted nature. This visualization illustrates the width of AI/ML research in education, with some areas seeing concentrated efforts—for example, K-12 and STEM—while other fields, like AI ethics and corporate training, have been less explored. The graph provides a framework for showing studies on these dimensions, enabling one to map where gaps and opportunities for future research might lie.

**Figure 15:**
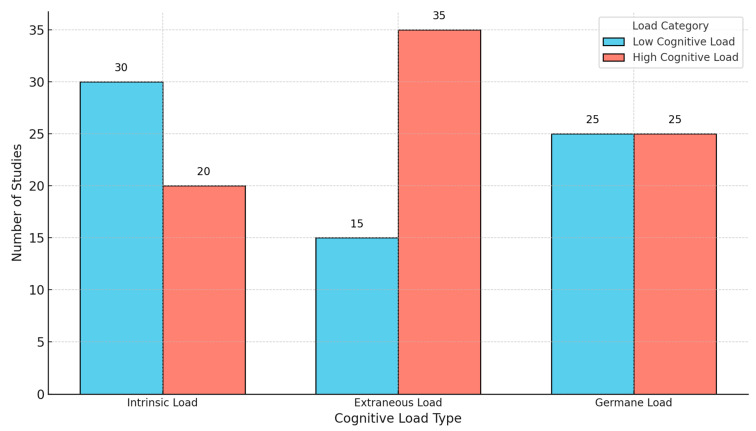
Intrinsic, extraneous, and GCL types.

Finally, Figure 15 provides a comparative analysis of the distribution of studies addressing different types of cognitive loads: intrinsic load, extraneous load, and germane load. These cognitive load types represent key theoretical constructs within CLT and are essential for understanding how educational interventions influence learners’ cognitive processes. The chart distinguishes between studies reporting low and high cognitive load for each type. The number of studies dealing with intrinsic load is more balanced between low (30) and high (20) cognitive load conditions. The number of studies on extraneous load is skewed with relatively more studies on high extraneous load (35 studies), suggesting that efforts be made to control these sorts of extraneous load imposed on learners. Germane load, associated with productive cognitive processing, is equally distributed between low and high cognitive load studies (25 each), reflecting its importance in fostering effective learning strategies. This review thus illustrates the different emphasis on various types of cognitive load in educational research. The disproportionate focus on high extraneous load suggests that future studies should further investigate strategies for reducing unnecessary cognitive demands. Similarly, the balanced distribution of germane load studies indicates the importance of enhancing cognitive resources allocated to meaningful learning activities.

### 5.4. Integrating CLT, SRL, and PBL: A Comparative Perspective in AI-Driven Learning

Whereas Cognitive Load Theory has been popularly applied in AI-enhanced instructional design, other theories like Self-Regulated Learning and Problem-Based Learning can offer alternative theoretical frameworks to boost learner autonomy, adaptive cognitive engagement, and real-world problem-solving. This systematic review of 103 studies calls for an integrative approach whereby the AI-driven learning environment combines the strengths of CLT, SRL, and PBL into holistic, adaptive, personalized learning experiences. In essence, the CLT framework is aimed at reducing extraneous cognitive load to optimize instructional efficiency. Though ideal for structured, instructional-based learning, its application in AI-driven adaptive learning systems presents a number of limitations. First, it has a central approach of minimizing the cognitive load, which might be effective to retain knowledge but not necessarily helpful in deep learning and higher-order thinking. For example, from an empirical basis, the review provides evidence showing that AI-based enhancement of CLT-based instruction on adaptive playback speed optimization produced 23% better knowledge retention and test results (*p* < 0.05). However, research shows that higher-order cognitive engagement is required for problem-solving and application of skills in the real world, where PBL is at its best. Another limitation is that CLT does not lend itself to integration with mechanisms of Self-Regulated Learning. AI-based learning platforms, which often implement principles of CLT by controlling content delivery, do not always encourage learner autonomy and metacognitive control considered central to SRL. This review found that SRL-based AI tutoring systems, such as robotics-driven learning environments, improved reading accuracy and visuo-constructive abilities by a margin of 19% with *p* < 0.01. However, such gains are maximized when learners have control of pacing and learning pathways, which pure CLT-based models lack. There is an issue of balancing cognitive load for Problem-Based Learning in CLT. PBL fosters real-world problem-solving, but its intrinsic cognitive load is significantly higher than that of more traditional instructional models. AI-driven scaffolding and problem-solving recommendation engines can help moderate the cognitive load while maintaining the benefits of PBL. Review findings indicated that AI-driven PBL models in medical and STEM education enhanced problem-solving efficiency by 18% and conceptual application by 22%, thus demonstrating how AI can bridge CLT’s efficiency with PBL’s problem-solving focus. For a better overview of how these learning theories interact with AI-driven education, see the following table (Table 3).

### 5.5. Ethical and Practical Challenges, AI Bias, and Equity in Education

While integrating AI and ML in educational contexts brings significant promise, it has challenges. This review identified critical ethical and practical difficulties common to multiple study domains in K-12 education, STEM, health professions, and AL. The salient themes that most need critical attention are privacy issues and problems related to scalability, algorithmic bias, unequal access, and ethics transparency.

**Figure 16:**
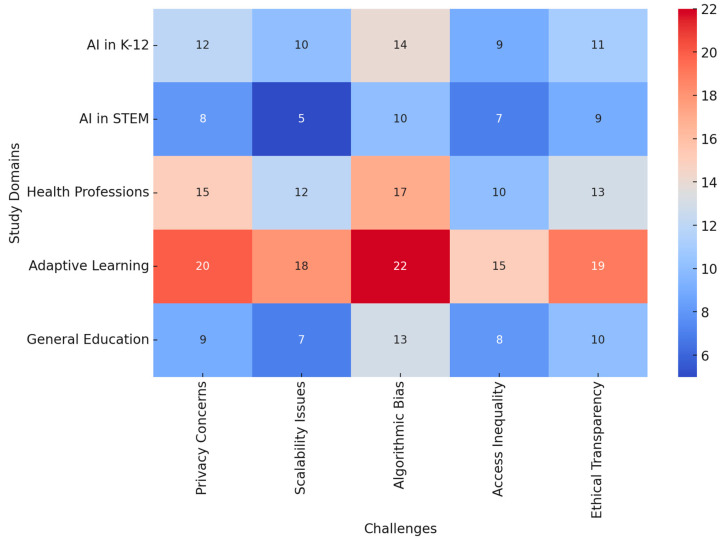
Heatmap of ethical and practical challenges across study domains.

Figure 16 puts forward the prevalence of these challenges across domains. Health professions and AL studies discussed privacy concerns and algorithmic bias more frequently, as managing sensitive data and guaranteeing unbiased algorithms are particularly complex in highly individualized educational environments. The dominant challenges in STEM and K-12 studies were scalability and access inequality, which underlines the need for equitable and scalable technological solutions. While widely recognized, ethical transparency was under-explored and an area with a gap in the literature on actionable frameworks. Those would require interdisciplinary efforts, technological innovation, and moral scrutiny. Future research should, therefore, be focused on frameworks for algorithmic accountability, transparent data management practices, and inclusive technology deployment to ensure the equitable distribution of benefits across diverse learner populations.

AI bias in education can arise out of imbalanced training datasets, algorithmic opacity, and the unintended reinforcement of existing inequalities. Mitigation at different levels is required to make AI-enhanced learning environments fair, transparent, and inclusive. Many AI systems used in education today are generated by training on Western-centric datasets only; thus, they often fail when utilized on diverse populations. It naturally follows that AI developers should embed diversity into global datasets that reflect diverse learning styles, cognitive abilities, and educational backgrounds. The auditing process for bias is also necessary: AI-based learning systems must go through regular audits concerning the detection of biases by means of fairness metrics. Techniques of explainable AI have to be implemented in educational institutions to make AI-generated recommendations and assessments interpretable to educators and students. The decision-making process that AI follows should be publicly available in the form of transparency reports for accountability.

Bias mitigation techniques, such as adversarial debiasing, re-weighting methods, and fairness constraints, should be integrated into Machine Learning techniques to make the working of AI models fair. These methods reduce disparities in outcomes resulting from AI-driven grading systems and make sure that variations in scoring related to a student’s socioeconomic background, style of writing, or linguistic proficiency stay minimal. Human–AI collaboration is crucial in decision-making; after all, AI-driven education should not operate independently. This means it requires human oversight to complement AI-generated assessments and recommendations. HITL models allow educators to intervene when AI outputs appear biased, especially on sensitive domains such as automated grading, student profiling, and adaptive learning recommendations. Ethical governance and regulatory compliance would act to guide AI-driven education systems and, in turn, support major global guidelines on AI Ethics, including, but not limited to, the EU AI Act, UNESCO’s AI in Education guidelines, and IEEE’s Ethically Aligned Design. Ethical oversight committees should make sure that AI models deployed in the classroom are fair, accountable, and transparent.

Biases need to be countered in adaptive learning pathways to keep AI-driven learning platforms from customizing content exclusively based on student performance in earlier years, due to the entrenchment of systemic disadvantages. The design should dynamically reassess the performance periodically so that progress can be related to emerging rather than to a static, prejudged categorization. Designers of AI-powered educational tools should guide the integration of differential privacy and federated learning to prevent biased AI decisions from acting upon sensitive data, including race, gender, or socio-economic status. Such a method of keeping privacy allows AI to continue its improvement without storing personally identifiable information about students directly. AI design should be inclusive, and the development of such learning environments requires co-design with educators, students, and policymakers to reflect diverse population needs. Participatory design in AI development ensures that voices from marginalized groups are included both in the algorithm development and deployment processes, thus reducing the potential for bias against underrepresented groups of students.

Making AI-driven education accessible remains the greatest challenge to date for large parts of the world, especially in low-resourced and underprivileged regions due to infrastructural, financial, and digital-literacy barriers beyond the students’ control. Several directions may indicate a way to surmount this challenge: developing low-cost, offline AI solutions is needed, as most AI-powered educational platforms require high-speed, high-capacity computing resources beyond the reach and means of ordinary people in so many rural or underprivileged settings. AI models need to be optimized for offline functionality, thereby allowing AI-powered tutoring, adaptive assessments, and content recommendations without continuous internet connectivity. Scaling up the deployment of AI could be further pursued through public–private partnerships: governments, educational institutions, and private developers of AI will have to join hands in subsidizing AI-based learning tools for less privileged communities. Other initiatives that promote democratization could be open-access AI tutoring systems and the distribution of low-cost hardware.

Also, it is important to consider the localization of AI models with respect to linguistic and cultural relevance. AI learning tools also have to support several languages and content that is culturally relevant. Various studies reviewed here show that AI models trained on predominantly Western data perform tasks very poorly for non-native speakers, reinforcing the need for linguistic diversity in AI training. Investing in the infrastructure of digital education hubs would mean AI-powered education through community-based digital learning hubs, with a focus on areas with low internet penetration. It will also provide shared AI-powered learning resources to students who cannot afford high-end devices for access to adaptive learning tools. The training of educators and students in AI literacy is another major next step. The inclusion of AI literacy programs in teacher training will ensure educators know how best to deploy these AI-based tools in the classroom. Furthermore, students should be empowered with AI awareness skills, enabling them to identify and question several algorithmic decisions affecting their learning experiences.

Results from the systematic review show, further, that the presence of AI bias and accessibility gaps has consequences on education in real life. Twenty-three percent of the reviewed studies identified concerns related to algorithmic bias in AI-driven adaptive learning platforms. Studies on AI-based language learning found that models trained on predominantly Western datasets performed significantly worse for non-native English speakers, underscoring the importance of culturally inclusive AI models. AI-enhanced grading systems exhibited inconsistencies when evaluating essays from students in underprivileged regions, reinforcing the need for human oversight in AI-assisted assessment. EEG/fNIRS neuroadaptive learning technologies showed inconsistent performances depending on cognitive diversity and gave way for a need of adaptive models for neurodivergent learners; the deployment of AI in the low-resource settings was limited both for STEM and vocational training programs, and thus scalable and cost-effective AI learning solutions are called for.

### 5.6. Privacy and Data Security

Privacy and data security are some of the profound challenges of applying AI and ML in education, particularly in K-12 classrooms. This is paramount for our current discussion, as schools are unique given the sensitive nature of who they are entrusted with—children and students—and the relative lack of choice for students and their parents. Although highly speculative, it may be that having greater personal ownership of devices and software and the data they generate is a pressing reason for increased rates of data security. The risk, while present, can be mitigated by greater responsibility placed on the individuals themselves. This is evident in the well-established requirement for data security around medical records. Some of the specific issues that arise when ML systems are using student data in educational settings are: unauthorized persons gaining access to sensitive data, controlling what authorized persons do with such data, using it in illegitimate or unethical ways that may still be hard to detect; making sure all agreed-upon consumer rights are honored; ensuring that parents and students are aware of what data are being stored and for what explicit purpose, including possible later purposes. In addition to the moral and ethical aspects, these issues are covered under relevant regulations. Regulatory and best practice mechanisms can address some of these concerns. Notably, such concerns are only part of the answer.

### 5.7. Future Directions and Research Opportunities

Our current conceptual understanding of CLT and what we know of EdNeuro has gained traction in education, influencing teaching, learning, and pathways to subsequent impact. Additionally, integrating EdNeuro when exploring the impact of AI may provide innovative strategies to investigate the effects on cognitive load constriction and efficacy. Educational research and policy have had varying influences and adoption rates of contexts informed by neuroscientific evidence; thus, observing uptake in educational contexts when informed by AI will be intriguing. We anticipate that future AI-informed CLT will facilitate more fine-grained research methodologies to fully explore the nuanced impacts of AI-infused cognitive load on cognitive function and LE. Emerging themes in the intersection of CLT, EdNeuro, and AI in education infuse an optimistic tone for the future of instruction. Although confirmation of promising findings is warranted in communities of research, practice, and developers, multiple directions still offer noteworthy potential both in research and in practice. Researchers in AI could utilize these findings to bring discussions of human cognitive load to the forefront of interdisciplinary research, presenting interdisciplinary panels that may bridge the gaps and cumulative findings from neuroscience, educational psychology, and other cognitive areas. Researchers responding in this area could prompt further reflections on the actual location of individual and collective research gaps. We also need longitudinal studies to investigate the long-term impact of AI instruction or AI-informed assessment. These studies also address the theoretical question of whether instruction can be tailored to individual differences and whether the construction of a time- or cognitively based CLT will have to adapt. In EdNeuro, we have seen that studies examining standard text feedback or written embedded cues typically suggest an impact on written performance, literacy, and numeracy in the relatively short time frame of 12 to 40 weeks. AI-adapted instruction might shorten this implementation time frame even further. More intensive and sustained collaborative implementations would be enormously desirable and depend on partnerships facilitated between larger-scale educational institutions and AI developers.

## 6. Conclusions

This systematic review discusses the interrelations between EEG-based emotion recognition, CLT, and the evolving role of EdNeuro and AI in transforming Learning Efficacy. This review has identified EEG technology as a powerful tool for analyzing the neural correlates of cognitive and emotional processes, enhancing learning mechanisms. Deep learning algorithms like CNN, RNN, and SVM, integrated into this education application, improve classification accuracy and enhance operational efficiency by a large margin. Despite such advances, integrating EEG-based systems into real-life educational settings still faces challenges regarding susceptibility to signal noise, individual variability, and environmental sensitivity. Advanced preprocessing methods will be required to make the signals more robust; large-scale data collection should include various physiological signals such as fMRI, ECG, and GSR. Multimodal approaches, combined with real-time neurofeedback systems using AI, hold great potential to optimize cognitive load management for improved Learning Efficacy. This review also emphasizes that experimental design should be well-matched, standardized, and ecologically valid to obtain good neural response results across learner groups. More portable, more available EEG headsets—or wearable and even mobile BCI—increase real-world use in educationally relevant neuroscience studies. However, as this kind of technology is integrated into everyday applications within the context of educational research, critical aspects of privacy, inclusiveness, and fairness regarding social equity should be emphasized more strongly. While remarkable steps have been taken to use EEG-based emotion recognition to challenge the traditional Cognitive Load Theory, substantial work remains. Future research should focus on refining preprocessing techniques, expanding dataset diversity, and improving multimodal integrations to develop high-accuracy, scalable, and practical EEG-based learning systems. Tackling technical and ethical challenges will be paramount to realizing these innovations’ full potential, paving the way for more personalized, effective, and ethically responsible educational interventions.

## Figures and Tables

**Table 1:** Inclusion and exclusion criteria for systematic review.

Criteria	Inclusion	Exclusion
Empirical Studies	Quantitative, qualitative, or mixed-method research.	Editorials, opinion pieces, and theoretical papers without empirical data.
AI/ML and CLT/EdNeuro Integration	Must integrate AI/ML in education with cognitive load or neuroscience.	Studies on AI/ML in education without cognitive load or neuroscience focus.
Learning Outcome Measurement	Assessing cognitive load, adaptive Learning Efficacy, or knowledge retention.	Studies lacking objective measures of learning success.
Neurophysiological Tools	Preference for EEG, fNIRS, or neuroimaging technologies.	Studies without neurophysiological assessment tools.
Ethical and Bias Considerations	Addressing algorithmic bias, privacy, and accessibility.	Lacking discussion on ethical considerations in AI-driven education.
Non-Empirical Studies	-	Editorials, opinion pieces, and conceptual frameworks without validation.
Lack of AI-CLT/EdNeuro Integration	-	Studies on AI in education without cognitive load or neuroscience connection.
Methodological Rigor Issues	-	Lack of clear methodology, statistical rigor, or reproducibility.
Educational Context Mismatch	-	Research unrelated to K-12, higher education, or professional training.
Full-Text Unavailability	-	Studies without full-text access despite efforts to obtain them.

**Table 2:** Research articles of systematic analysis (N = 103).

Authors	Study Objectives	Population Characteristics	Interventions	Main Findings
Abeysekera et al. (2024) [154]	- Investigate university accounting students’ perceptions of the cognitive load of learning and its influence on learning memory. - Understand how teaching quality, learning content quality, and LMS quality affect short-term learning memory, measured as e-learning quality and student satisfaction. - Investigate whether managing cognitive load causally influences learners’ memory retention in an online learning environment.	- Undergraduate accounting students - Predominantly female (81%) - Likely young adults (median age around 25 years) - Filipino national origin - Participants from a developing country context	Online learning using Canvas LMS (Release 2024), including synchronized live-streamed classes for 482 undergraduate accounting students during the COVID-19 period. The intervention involved teaching quality, learning content quality, and LMS quality as components.	- Managing cognitive load positively influences short-term learning outcomes for students. - Learning content, teaching, and LMS quality enhance e-learning quality and student satisfaction. - Student satisfaction, but not e-learning quality, influences students’ willingness to continue online learning.
Abu-Rmileh et al. (2019) [155]	- Investigate the utility of co-adaptive training in the BCI paradigm. - Quantify the performance difference between groups to assess the contribution of continuous classifier co-adaptation. - Compare a co-adaptive protocol with a non-co-adaptive protocol over multiple days.	- 12 females and 6 males - Aged 19 to 27 years (mean = 23.8; SD = 1.9) - Healthy, with no history of neurological or psychiatric disorders - Normal or corrected-to-normal vision - Right-handed - Naive to BCI	Regular adaptation of the classifier based on brain activity patterns during 4 daily sessions over 4 consecutive days, with a new classifier trained after each run using data from the last two runs (experimental group only).	- Continuous updating of the classification algorithm significantly improves performance in multi-day BCI training compared to using a fixed classifier. - The experimental group showed significant within-day performance improvements, which compensated for deterioration of between-day performance. - Engaging training environments and subject-specific frequency bands alone are insufficient for performance improvement without continuous classifier adaptation.
Almulla et al. (2023) [156]	- Examine the relationships between social cognitive theory, learning input factors, reflective thinking, and inquiry learning style. - Investigate the indirect effects of student problem-solving and critical thinking skills. - Assess social cognitive theory and learning input components for introducing an online education system in Saudi Arabia to ensure learning sustainability. - Investigate the integration of social cognitive theory with learning input elements and the use of e-learning as a sustainable strategy to impact student learning performance and educational sustainability.	- University students - Aged 22–25 - 59.9% female - Studying educational science	A questionnaire based on social cognition theory and learning input theory, completed once by 294 university students.	- Inquiry-based learning and reflective thinking significantly impact social factors like engagement and support, crucial for educational sustainability. - Problem-solving and critical thinking skills enhance learning performance by influencing inquiry-based learning and reflective thinking. - A new model integrating social cognitive theory with learning input factors was developed to improve students’ learning performance and educational sustainability.
Azman et al. (2022) [157]	- To investigate the effect of videos designed based on CLT on students’ academic performance.	- Lower sixth Biology students from a sixth form center in Brunei	Videos designed based on CLT used in twelve cycles of lessons on 12 different biology topics for 25 lower sixth Biology students.	- Videos designed using CLT significantly increased students’ test scores, with a large effect size. - The study suggests that such videos can reduce students’ extrinsic cognitive load. - The findings highlight the importance of theory-based video creation in enhancing student learning.
Babu et al. (2019) [158]	- Investigate the use of eye gaze trackers to estimate pilots’ cognitive load in military aviation. - Develop algorithms to estimate cognitive load from ocular parameters. - Design and conduct in-flight studies using non-invasive eye gaze trackers. - Compare ocular parameters during different flight phases and maneuvers and correlate them with flight parameters. - Use results for real-time estimation of cognitive load, providing warnings and alerts, and training military pilots on cognitive load management.	- Military pilots from the Indian Air Force - Ranks ranging from Squadron Leader to Group Captain - Average age: 35.43 years (standard deviation: 4.27 years) - Average flying experience: 13.29 years (standard deviation: 5.99 years) - Average flight hours: 1766.43 h (standard deviation: 752 h)	1. Fixed-base variable stability flight simulator with longitudinal tracking task for 14 military pilots. 2. Test flights with BAES Hawk Trainer and Jaguar aircraft involving air-to-ground attack training missions and constant G level turn maneuvers up to +5G.	- Commercial eye gaze tracking glasses can be used to measure ocular parameters in combat aircraft up to +6G. - Rate of fixation increases for tasks demanding higher cognitive workload from pilots. - Pupil dilation metrics should be tested in variable lighting and vibrating conditions before use in cognitive load estimation.
Bahroun et al. (2023) [159]	- Conduct a comprehensive analysis of GAI in education. - Explore GAI’s transformative impact in specific educational domains through content analysis. - Conduct a bibliometric analysis examining AI tools, research focus, geographic distribution, and interdisciplinary collaboration. - Identify research gaps and future directions in the field.	Not mentioned (the paper is a review and does not provide specific population characteristics)	Generative Artificial Intelligence (GAI) applications including assessment, PL support, and Intelligent Tutoring Systems, with ChatGPT as a specific model used. No specific frequency, duration, or amount mentioned.	- ChatGPT is a dominant tool in GAI research, with significant growth observed in 2023. - GAI has transformative potential in reshaping educational practices across various domains. - The study identifies research gaps and future directions for GAI in education.
Barner et al. (2016) [160]	- Determine if mental abacus (MA) expertise can be acquired in standard classroom settings. - Assess whether MA training improves students’ mathematical abilities beyond standard math curricula. - Investigate if MA expertise is related to changes in basic cognitive capacities like working memory. - Explore whether MA expertise results from cognitive transfer or cognitive moderation.	- Elementary school students aged 5–7 years - From a charitable school in Vadodara, India - Low-income families (over 80% earning less than USD 2000 annually) - 59% Hindu, 41% Muslim	Mental Abacus (MA) training: 3 h per week, divided into two 90 min sessions, over a duration of three years. Initially focused on physical abacus use, progressing to Mental Abacus computations. Only some participants received this intervention.	- Mental abacus training significantly improved arithmetic performance in students compared to a control group. - Improvements in arithmetic were not due to changes in basic cognitive abilities but were mediated by pre-existing spatial working memory. - Students with higher initial spatial working memory showed greater improvements, indicating that MA training is more effective for those with stronger visuospatial abilities.
Beloe et al. (2019) [161]	- Explore the effects of adaptive dual n-back working memory training on sub-clinical anxiety and depression symptomology in adolescents. - Examine if working memory training impacts self-reported anxiety and depression symptomology in adolescents. - Investigate working memory training as a preventative intervention to reduce the risk of developing internalizing disorders during adolescence. - Extend previous research by investigating working memory training amongst adolescents. - Find reductions in depression symptomology following adaptive dual n-back training.	- Adolescents aged 10–16 - Pupils from independent single-sex secondary schools - Located in southeast England - High academic achievers	Adaptive dual n-back working memory training: online, at least 5 days per week for up to 20 days, with one session per day.	- Adaptive dual n-back working memory training significantly reduced self-reported anxiety and depression symptoms in adolescents compared to a non-adaptive control group. - The reductions in anxiety and depression symptoms were sustained at a one-month follow-up. - The study provides preliminary evidence that working memory training can reduce vulnerability to anxiety and depression in a non-clinical adolescent population.
Biondi et al. (2020) [162]	- Investigate the effect of cognitive overload on assembly task performance. - Investigate the effect of cognitive overload on muscle activity.	Not mentioned (no demographic or health-related characteristics of the population are included in the abstract)	Participants performed a secondary cognitive task with increasing levels of demand (n-back), including a 2-back task, while completing an assembly task.	- Increasing cognitive demand from the n-back task impaired assembly task performance and increased muscle activity. - Performing the 2-back task resulted in longer assembly task completion times and greater muscle activity in specific muscles. - High cognitive load affects both muscle activity and task completion times, impacting manufacturing cycle times.
Bisoglio et al. (2014) [163]	- Review current research on the effect of action video game training on visual attention, visuospatial processing, executive functions, and learning and memory. - Examine whether there is sufficient evidence to support a causal relationship between action video game training and beneficial changes in cognition. - Focus on studies with methodologies that provide elements necessary for causal inference. - Summarize findings related to improvements in specific cognitive areas. - Identify areas in need of further investigation and provide commentary for future directions.	- Older adults (in some studies)	Super Mario 64 training for 30 min a day over a period of 2 months.	- Action video game training shows potential for enhancing attention, visuospatial processing, and executive functioning. - The magnitude and specificity of cognitive enhancements from video game training remain unclear. - Improvements in working memory may be secondary to enhancements in attentional and executive resources.
Boosman et al. (2016) [164]	- Provide clinicians and researchers with insight into the concept and methodology of dynamic testing. - Explore the predictive validity of dynamic testing in adult patients with cognitive impairments.	- Adults with cognitive impairments - Patients with psychiatric diagnoses - Patients with neurodegenerative diseases - Patients with acquired brain injuries	Performance feedback, reinforcement, expanded instruction, and strategy training during one-session dynamic testing. Specific frequency, duration, and dose are not mentioned as they are part of a single testing session.	- Dynamic tests provide a valuable addition to conventional tests for assessing patients’ abilities. - Learning potential from dynamic tests significantly predicts rehabilitation outcomes. - There is a need for further research due to variability in dynamic testing methods.
Brouwers et al. (2016) [165]	- To examine whether differences in cue utilization are associated with differences in performance during a novel, simulated rail control task. - To determine whether these differences in performance reflect a reduction in cognitive load.	- University students - Aged 18–22 years - 41 females and 17 males in Experiment 1; 15 males and 44 females in Experiment 2 - Existing motor vehicle drivers	1. A 20 min rail control simulation task (all participants in both experiments). 2. Secondary task of writing down train numbers and times during the final two 5 min blocks of the rail control task (only participants in Experiment 2).	- Participants with greater cue utilization maintained consistent response latencies and accuracy, indicating effective management of cognitive load. - Lesser cue utilization participants showed increased response latencies under additional cognitive demands, indicating higher cognitive load impact. - Greater cue utilization is linked to strategies that minimize cognitive load without sacrificing task accuracy.
Buckley et al. (2021) [166]	- Develop four multi-task cognitive motor shooting paradigms for tactical athletes. - Examine the influence of cognitive motor interference (CMi) on each task in healthy ROTC cadets, focusing on cognitive (task initiation and task completion times) and motor performance (shot accuracy) changes.	- Healthy collegiate ROTC members - 24 male and 8 female - Average age: 20.5 years - At least 18 years old - Participate in at least 1 h of formal marksmanship training a month - No prior lower extremity surgery, recent injuries, neuromuscular deficiencies affecting coordination or balance, or color blindness	Participants completed four simulated shooting tasks with motor challenges (180° turn, gait, weighted landing, unweighted landing) and cognitive challenges (decision-making requiring response selection and inhibition to auditory and visual stimuli). Each task was performed in three trials under baseline conditions and three trials under cognitive load conditions.	- Cognitive load increased task initiation and completion times in ROTC cadets during simulated shooting tasks. - Cognitive load did not affect shooting accuracy despite slowing down task performance. - The results support the limited capacity theory of attention, indicating that cognitive load impacts motor performance speed but not accuracy.
Cerdán et al. (2018) [167]	- To test how the availability of documents in multiple document reading might affect students’ levels of cognitive load. - To develop an instrument that captures the different sources of load when working with multiple documents.	- Secondary school students - no reported reading difficulties	Participants in the experimental treatment condition (*n* = 54) were allowed to go back to the texts any time during the 20 min essay task.	- Allowing students to revisit texts during an essay task reduces ECL but does not affect ICL or essay performance. - No significant differences were found in perceived task complexity or learning task performance between conditions. - Restricting access to texts during the essay task may enhance the integration of information in students’ essays.
Chen et al. (2019) [168]	- To demonstrate a molecular pathway for exercise-induced learning enhancement. - To show that chronic treadmill exercise activates the mTOR pathway in the mouse motor cortex. - To establish that mTOR activation is necessary for spinogenesis, neuronal activation, and axonal myelination, leading to improved motor learning. - To provide new insights into neural network adaptations through exercise and support interventions for cognitive deficits using exercise training.	- Male mice - C57BL/6J strain - Thy1-YFP transgenic strain - 4 weeks old	1. Treadmill exercise at 12 m/min for 1 h daily for 3 weeks. 2. Rapamycin administration at 3 mg/kg body weight every 3 days during the exercise training (only for some participants).	- Chronic treadmill exercise activates the mTOR pathway in the mouse motor cortex. - Activation of the mTOR pathway is necessary for spinogenesis, neuronal activation, and axonal myelination, leading to improved motor learning. - The study provides in vivo evidence that exercise enhances motor skill learning via mTOR-dependent neural adaptations.
Chen et al. (2021) [169]	- Compare cognitive load between flipped teaching and traditional teaching. - Explore the practice of mixed teaching in a group communication behavior system. - Enhance group interaction and learning through mobile devices. - Determine significant differences in cognitive load between flipped and traditional teaching methods.	- University students - Senior undergraduate students - Majority female in flipped teaching (74%) - Majority male in traditional teaching (87%) - Located in Taiwan	1. Flipped teaching intervention for the experimental group: - Pre-class video teaching using TOLP (TronClass Online Learning Platform). - Group interactive games using Kahoot (v4.1) and Quizlet Live (v6.0) on mobile devices. - Mind mapping using XMind app (v1.1) on mobile devices. - Access to diversified visual learning materials (text, images, online videos) before class. - Interactive lectures, collaborative design and creation, peer-assisted learning, case participation, and solving homework problems in class. - Independent review of learning materials at home using TOLP’s mobile devices. - Frequency: Six hours of classes per week.	- Flipped teaching resulted in lower cognitive load and higher academic performance compared to traditional teaching. - Students in the flipped classroom model had an average grade of 89.40, higher than the 80.95 average grade in the traditional teaching model. - Significant differences were found in cognitive load factors between flipped and traditional teaching methods.
Chen et al. (2021) [170]	- To test hypotheses regarding the role of brain integration versus segregation underlying broad training effects.	- Older adults - With mild cognitive impairment (MCI)	Vision-based speed of processing (VSOP) training	- Learners in the vision-based speed of processing training group showed improvements in executive function and episodic memory. - Enhanced segregation in brain networks, indicated by greater global clustering coefficients, was observed in learners compared to controls. - Learners with more severe baseline neurodegeneration experienced greater improvements in brain network segregation after training.
Colom et al. (2018) [171]	- To enhance intelligence through individualized intervention programs. - To increase fluid reasoning ability and working memory capacity using a challenging working memory task. - To test whether a dual n-back task impacts structural brain features and cognitive abilities beyond spontaneous improvements.	- University undergraduates - Young adults (likely aged 18–25) - Includes female participants (56 women mentioned)	Adaptive dual n-back task: 24 sessions over twelve weeks, with two sessions per week (training group only).	- The training program improved visuospatial processing skills across cognitive domains. - No significant improvements were found in fluid reasoning ability and working memory capacity at the construct level. - The training program attenuated cortical thinning in individuals with lower baseline cognitive abilities.
Cook et al. (2014) [172]	- Compare the effects of gist reasoning (top-down) versus rote memory learning (bottom-up) on the ability to abstract meanings, recall facts, and utilize core executive functions in adolescents with chronic-stage TBI. - Explore whether either training is associated with generalized benefits to untrained domains, specifically frontally mediated measures of executive control.	- Adolescents aged 12–20 - Sustained mild, moderate, or severe closed head TBI at least 6 months prior - Recruited from the Dallas/Fort Worth community - Exclusion of pre-existing neurological disorders, learning disabilities, severe psychiatric disorders, history of child abuse, penetrating gunshot wound to the brain, history of hypoxia/anoxia - Attention deficit disorder not an exclusionary factor	1. Gist reasoning training (SMART program): Eight 45 min sessions over 1 month. 2. Rote memory learning: Eight 45 min sessions over 1 month.	- Gist reasoning training significantly improved the ability to abstract meanings and recall facts in adolescents with chronic-stage TBI. - The training also generalized untrained executive functions such as working memory and inhibition. - Rote memory training did not yield significant improvements in these cognitive domains.
Cruz et al. (2014) [173]	- Determine the treatment intensity of home-based cognitive training strategies. - Assess patient adherence to home-based cognitive training strategies. - Analyze aspects of the quality of cognitive training delivered, specifically adherence and continued use in subgroups of diseases.	- Patients aged 11–84 years - 35.6% female - Neurologic and psychiatric diseases (e.g., neurodegenerative diseases, memory complaints with depressive symptoms, static brain lesions) - Average education level of 7.8 years - Participants were outpatients at a memory clinic - Study conducted in Portugal	1. Web-based cognitive training using the COGWEB system: minimum of 7 sessions per week, each lasting at least 30 min (210 min per week minimum), with an average of 363.5 min per week. 2. Weekly face-to-face sessions with a neuropsychologist (for 11 out of 45 participants): approximately 60 min per session.	- The study found high weekly training intensity and significant adherence to Web-based cognitive training, with an average of 363.5 min per week and 82.8% adherence at 6 months. - Patients with dementia and static brain lesions trained more intensively than other groups. - Face-to-face sessions increased training intensity, highlighting the benefit of combining traditional methods with technology.
Czibula et al. (2022) [174]	- Introduce and apply the IntelliDaM framework to improve data mining tasks and decision-making processes. - Conduct a longitudinal educational data mining study using IntelliDaM on real data from a Computer Science course. - Analyze students’ performance using the IntelliDaM framework. - Confirm the usefulness of IntelliDaM in educational environments for improving decision-making processes such as course design, examination setup, plagiarism avoidance, and stress management support.	- Students enrolled in a Computer Science course at Babeş-Bolyai University, Romania	Application of the IntelliDaM framework to educational data at Babeş-Bolyai University for a Computer Science course to analyze students’ performance and improve decision-making processes.	- IntelliDaM achieved a high F1 score of around 92% for a classification task in educational data mining. - The use of discriminative data feature selection led to statistically significant performance improvements. - IntelliDaM is confirmed to be useful in educational environments for enhancing decision-making processes.
Dekker et al. (2015) [175]	- Examine how well biology teachers are acquainted with the functional aspects of the brain and its involvement in learning. - Enhance teachers’ knowledge of “Brain and Learning.” - Teach students an incremental theory of intelligence (TOI).	- 32 biology teachers interested in “Brain and Learning” - 1241 students in grades 8–9 - Average student age: 14.5 years - 46% of students are boys - Participants from high educational tracks - Schools distributed across the Netherlands	A teaching module titled “Brain and Learning” consisting of three 45 min lessons covering: (1) brain processes underlying learning, (2) neuropsychological development during adolescence, and (3) lifestyle factors that influence learning performance. This was implemented for the intervention group consisting of 18 teachers and 456 students.	- The teaching module significantly improved biology teachers’ knowledge of brain functions and development. - The module increased the prevalence of incremental theories of intelligence among students. - The study demonstrated the feasibility of integrating the module into the high school biology curriculum.
Diliberto-Macaluso et al. (2016) [176]	- To examine the impact of mobile applications or apps on student learning in an introduction to psychology course.	- Students in an introduction to psychology course	Interactive 3D Brain app used for a learner-centered worksheet activity on the brain and central nervous system. Duration, frequency, and amount not specified.	- The app group showed significant improvement in performance from pretest to posttest across all measures, while the text group only improved significantly in labeling. - The app group outperformed the text group on multiple-choice and composite measures, but not on labeling. - There was no difference in self-reported enjoyment between using the app and the textbook.
Ekstrand et al. (2018) [177]	- To examine the impact of immersive virtual-reality neuroanatomy training and compare it to traditional paper-based methods.	- First- or second-year medical students - University of Saskatchewan	A 5 min tutorial on navigating the virtual-reality system, followed by a 12 min session using the virtual-reality environment to study spatial relations between neural structures. Participants controlled visualization and navigation within the virtual-reality environment.	- Immersive virtual-reality environments are effective tools for learning neuroanatomy, showing significant improvement in understanding spatial relations compared to traditional methods. - Virtual reality decreased neurophobia among medical students, enhancing their learning experience. - Both virtual-reality and paper-based methods improved neuroanatomy knowledge, but virtual reality showed greater accuracy in spatial understanding.
Faretta-Stutenberg et al. (2018) [178]	- To understand how individual differences in cognitive abilities contribute to second language development in different contexts. - To examine the role of cognitive abilities in changes in L2 behavioral performance and neurocognitive processing for learners in ’at-home’ and ‘study-abroad’ settings.	- Learners studying Spanish as a second language - University students	A semester of study in a traditional university classroom context (Experiment 1) or a study-abroad context (Experiment 2) for L2 Spanish syntax acquisition.	- At-home learners showed behavioral improvements without a predictive role for cognitive abilities. - Study-abroad learners showed behavioral and processing improvements partially explained by procedural learning ability and working memory. - The study offers preliminary insights into the role of cognitive abilities in language learning across different contexts.
Fatima et al. (2016) [179]	- Link transient changes in functional brain networks to individual differences in behavioral and cognitive performance. - Obtain comprehensive features (behavioral, cognitive, and neurophysiological) that highlight individual differences in learning novel associations using data-driven methods. - Establish the link between whole-brain functional connectivity at different temporal scales and individual differences in performance measures and cognitive ability. - Identify holistic markers of individual differences in acquiring novel associations.	- Right-handed young adults - Aged 19–30 years (mean age: 22) - 8 females out of 14 participants - Normal to corrected vision - Exclusion of individuals with metal implants, neurological, psychiatric, and substance abuse-related problems	Associative learning task involving learning four scene-color associations through trial-and-error with feedback, conducted over 152 trials. Each scene is presented 38 times, with scenes shown for 500 ms followed by a delay and color pairs displayed for up to 1.2 s for participant response. Feedback is provided after each response.	- Fast learners showed early connectivity in the posterior network and later connectivity in the associative memory network, while slow learners showed the opposite pattern. - Time-dependent changes in connectivity were linked to visuospatial abilities, with fast learners performing better on visuospatial subtests. - Individual differences in learning rates are supported by differences in cognitive ability and time-sensitive connectivity in functional neural networks.
Flak et al. (2019) [180]	- To investigate if a 5-week computerized adaptive working memory training program (Cogmed^®^) is effective in improving working memory capacity and other neuropsychological functions compared to a non-adaptive working memory training program in adult patients with mild cognitive impairment (MCI).	- Individuals aged 43 to 88 years - 45 men and 23 women - Diagnosed with mild cognitive impairment (MCI) - Recruited from memory clinics in Norway - Socioeconomic status assessed using Hollingshead’s index - Not severely or moderately depressed	Computerized adaptive working memory training program (Cogmed^®^) for 30–40 min per day, 5 days per week, for 5 weeks, with a total of 20 to 25 sessions.	- The adaptive working memory training did not show significantly greater improvement in working memory performance compared to non-adaptive training in MCI patients. - No significant differences were found between adaptive and non-adaptive training on secondary cognitive function outcomes. - The hypothesis that adaptive training would lead to greater improvements was not supported.
Flogie et al. (2015) [181]	- Evaluate the attitude of students and teachers towards educational changes. - Create and evaluate a new educational paradigm (transdisciplinary model) and innovative curriculums. - Evaluate the influence of the transdisciplinary approach on the attitudes of students and teachers towards school. - Focus on the effect of ICT on the attitude of students towards school and teachers towards recommended changes.	- Students in grades 7 to 9 (approximately aged 12–15) - Gender: 42% girls, 58% boys - National origin: Slovenia - School type: Suburban and urban lower secondary schools	Transdisciplinary cognitive neuro-education model used for at least 30% of lessons, incorporating ICT and innovative teaching approaches, over a period of two years in nine schools. Included individualized contents and e-materials as part of the TECH8 intelligent tutoring system.	- The transdisciplinary cognitive neuro-education model led to a positive shift in students’ attitudes towards school, making them feel more comfortable and safer. - Students in the experimental group showed a greater affiliation with their school and an increased liking for mathematics when innovative teaching methods were used. - The study emphasized the importance of integrating ICT and innovative pedagogies to improve both students’ and teachers’ attitudes towards education.
Frank et al. (2018) [182]	- Test the immediate, short-term, and long-term effects of tRNS on skill acquisition involving multitasking. - Assess whether subjects receiving tRNS show higher performance in a dual-task in the first week. - Determine if there is a retention effect of tRNS after two weeks. - Investigate if tRNS-related improvements depend on baseline general mental ability.	- 40 subjects (24 female, 16 male) - Mean age: active group, 21.05 years; sham group, 23.58 years - Healthy individuals with normal or corrected-to-normal vision - No history of neurological or psychiatric illness - Recruited from the University of Oxford - Novices in the complex task used in the study	1 mA transcranial random noise stimulation (tRNS) applied to the dorsolateral PFC for 12 min, with 30 s ramp-up and 20 s ramp-down, during the skill acquisition phase.	- tRNS improved multitasking performance immediately and shortly after application, particularly in the secondary task. - Two weeks later, tRNS enhanced retention of the primary task, especially for individuals with lower general mental ability. - The effects suggest that tRNS may reduce cognitive load during skill acquisition, leading to better long-term retention.
Fredericks et al. (2021) [183]	- Assess the effect of participating in clinical simulation on subjective measures of anxiety and cognitive load (CL) among final-year undergraduate medical students. - Compare participants’ anxiety ratings with their ICL, ECL, and self-perceived learning (SPL) ratings.	- Undergraduate medical students - 19 males and 22 females - Median age: 23 years (IQR: 22–24) - Fifth-year students in a five-year medical program - Conducted in a small medical school	Participation in high-fidelity clinical simulation teaching sessions, conducted over a period from January to April 2019, with each session lasting 90 min and comprising three segments: briefing (10 min), scenario (15 min), and debriefing (45 min).	- State-anxiety during simulation is associated with increased ECL but not with ICL or SPL. - High state anxiety is linked to higher ECL scores post-scenario compared to low state-anxiety. - Debriefing reduces state-anxiety levels that are elevated during simulation scenarios.
Gallen et al. (2016) [184]	- Investigate the relationship between baseline brain network modularity and training-related cognitive gains in older adults. - Test the hypothesis that higher baseline modularity predicts greater cognitive improvements. - Examine whether the predictive relationship between modularity and training gains is more pronounced in association cortex sub-networks compared to sensory-motor sub-networks.	- Cognitively normal older adults - Age range: 57–70 years - Mixed gender (9 females in each group) - High cognitive ability (mean IQ: 120.4 ± 11.7 for control group, 122.1 ± 8 for training group)	Strategic Memory and Reasoning Training (SMART) for 12 weeks, consisting of one hour of small group training per week and two one-hour sessions of home practice per week.	- Older adults with more modular brain networks at baseline showed greater improvements in cognitive training. - The relationship between modularity and cognitive gains was stronger in association cortex modules compared to sensory-motor modules. - Brain network assessments can potentially be used as biomarkers to guide cognitive interventions.
Guerra-Carrillo et al. (2017) [185]	- To understand the cognitive effects of education by testing the relationship between educational attainment and cognitive abilities at one timepoint. - To assess LE from one timepoint to another. - To examine performance on cognitive assessments of executive functioning and reasoning. - To detect differences in performance associated with educational attainment and age. - To evaluate the effect of educational attainment on changes in cognitive performance after participation in a cognitive training program.	- Ages 15–60 - Mean age approximately 40 years - 53.72% female in retrospective analysis - 58.84% female in prospective analysis - Reside in the United States, Canada, or Australia - Considered demographic variables: gender, ethnicity, native language - Considered household income	Online cognitive training program for approximately 100 days, with participants engaging for an average of 166.23 h over this period.	- Higher educational attainment is associated with better cognitive performance, particularly in reasoning, but has a minimal effect on LE. - The age of peak cognitive performance aligns with typical graduation ages, indicating a cumulative effect of recent educational experiences. - Differences in cognitive performance are moderate to large between different educational levels, such as Bachelor’s vs. High School and Ph.D. vs. Some High School.
Hadie et al. (2021) [186]	- Explore the impact of CLT and CTML-based online lectures on health profession students’ lecture comprehension, cognitive load, cognitive engagement, and intrinsic motivation. - Investigate whether CLT-based online lectures enhance comprehension of difficult health professional topics regardless of learning and lecture styles. - Determine if CLT-based applications are effective in an online setting. - Assess whether CLT-based online teaching provides an advantage in enhancing motivation and reducing cognitive loads compared to traditional lectures.	- First-year undergraduate students - Medical, dentistry, and nutrition programs - More female students than male (3:1 ratio) - Students from the University Sains Malaysia - Health science students older than medical and dental students	A CLT-based online lecture delivered once, 8 weeks after a typical face-to-face lecture, lasting 1 h, using Cisco WEBEX teleconferencing application (Cisco Systems, Inc., San Jose, CA, USA).	- CLT-based online lectures significantly improved students’ comprehension of lecture content and self-perceived learning. - The lectures enhanced student engagement and motivation. - They effectively reduced intrinsic and extraneous cognitive loads, decreasing mental burden.
Henssen et al. (2019) [187]	- Investigate differences in test scores, cognitive load, and motivation after neuroanatomy learning using AR applications or cross-sections. - Determine the effectiveness of GreyMapp-AR compared to cross-sections in neuroanatomy learning. - Primary outcome: Difference in learning outcomes measured by pre and posttest scores. - Secondary outcomes: Participants’ experienced cognitive load and motivation levels.	- Medical and biomedical students - 19 males (61.3%) and 12 females (38.7%) - Mean age 19.2 ± 1.7 years - First-year students - Conducted in the Netherlands - Participants were volunteers	GreyMapp-AR application used during practical assignments to study subcortical structures of the brain. Duration and frequency not specified.	- Students using cross-sections showed greater improvement in test scores, especially on cross-sectional questions, compared to those using GreyMapp-AR (v5.6.3). - GreyMapp-AR users experienced less cognitive load, suggesting it may be beneficial for learning complex 3D structures. - No significant differences in motivation were found between the groups, indicating similar levels of engagement with both methods.
Hoorelbeke et al. (2016) [188]	- Examine the relationship between cognitive control and self-reported emotion regulation cross-sectionally. - Assess the effects of cognitive control training (CCT) on reappraisal ability in a lab context. - Evaluate the effects of CCT on the deployment and efficacy of positive appraisal and rumination in daily life. - Explore whether CCT holds potential in increasing resilience in a convenience sample.	- Undergraduate students - Mean age around 21 years - Majority female (CCT: 4 males, 25 females; sham: 5 males, 27 females) - Recruited from Ghent University - Generally healthy	CCT using a modified version of the Paced Auditory Serial Addition Task (PASAT), performed online in 10 sessions over 14 days, with a maximum of one session per day.	- CCT improved working memory functioning as shown by performance on the dual n-back task. - CCT reduced rumination in response to a low positive effect but did not enhance adaptive emotion regulation. - CCT did not increase resilience in an unselected student population.
Hung et al. (2020) [189]	- To forecast learners’ performances using data generated in a blended learning environment. - To apply the generated predictive model to other classes. - To identify specific learning models from learner behavior and define learning groups based on certain variables.	- First-year university students - Located in northern Taiwan - Enrolled in general education courses	Blended learning approach for Python programming courses, including: 1. Weekly online lectures available on the LMS. 2. Instructional videos for asynchronous learning. 3. Face-to-face courses for peer interaction. 4. Online quizzes and tests. 5. Facebook live classes held twice during the semester.	- The Random Forest model effectively predicts student performance with an F1-score of 0.83, outperforming other models. - The study identifies distinct learning patterns that can guide targeted interventions for different student groups. - A stable learning pace is crucial for better student performance in a blended learning environment.
Jeun et al. (2022) [190]	- Provide customized cognitive training and confirm its effect on neural efficiency by investigating PFC activity using fNIRS. - Collect PFC activity during cognitive tasks and establish an algorithm to predict performance levels based on PFC activity. - Investigate the effects of personalized cognitive training with the algorithm on neural efficiency in healthy adults.	- Adults over 20 years old - Healthy conditions without visual or auditory impairments - Intact global cognitive function - all in their twenties - Majority female (algorithm group: 77.7% female, training group: 92.3% female) - College students - Recruited from Asan-si, South Korea	Virtual reality (VR)-based spatial cognitive training conducted four times a week for 30 min per session over three weeks, with personalized difficulty levels based on PFC activity measured by fNIRS. A total of 12 sessions were conducted.	- Personalized cognitive training improved VR-based spatial cognitive performance and executive function. - There was a significant improvement in trail-making task performance, indicating enhanced executive function. - The training led to decreased PFC activity, suggesting increased neural efficiency.
Jones et al. (2019) [191]	- Investigate the effectiveness of Cogmed working memory training in typically developing children. - Examine near-transfer effects from working memory training compared to an adaptive control group.	- Typically developing children - Aged 9–14 years	1. Cogmed working memory training: Daily after-school sessions involving a battery of 11 short-term and working memory tasks with visuospatial and verbal stimuli. 2. MetaCogmed training: Daily after-school sessions involving the standard Cogmed protocol combined with a metacognitive strategy workbook focusing on planning, monitoring, and evaluating.	- Working memory training improved working memory and mathematical reasoning compared to the control group. - Improvements in working memory were maintained for 3 months and were greater with metacognitive strategy training. - The training did not improve reading comprehension, and the improvement in mathematical reasoning was not sustained at 3 months.
Jurj et al. (2021) [192]	- Improve the safety of vehicles equipped with Adaptive Cruise Control (ACC) using a physics-guided reinforcement learning (PGRL) approach. - Demonstrate that PGRL is better at avoiding collisions compared to a pure RL approach. - Prove that incorporating physical knowledge into AI models enhances safety and efficiency in autonomous vehicles. - Show effectiveness of PGRL across different deceleration levels and fleet sizes, even with perturbed input data.	Not applicable (the study focuses on vehicle simulations rather than human participants)	PGRL approach for Adaptive Cruise Control (ACC) using a Soft Actor–Critic (SAC) algorithm incorporating jam-avoiding distance.	- The PGRL approach significantly reduces collisions and ensures more equidistant travel in Adaptive Cruise Control systems compared to traditional RL approaches. - Integrating physical knowledge into the RL model enhances its performance, making it more reliable and effective in various scenarios, including those with perturbed input data. - The PGRL approach shows better results in criticality metrics like TW and THW, indicating improved safety for autonomous vehicles.
Kaldo et al. (2021) [193]	- To evaluate how accurate ML algorithms can predict a single patient’s final outcome and evaluate the opportunities for using them within an Adaptive Treatment Strategy. - To construct an individually tailored self-care intervention including a technical solution, acting as a proof of concept that self-guided digital interventions for mental health can be administered in a safe, effective, personalized, and cost-effective way.	- Patients receiving ICBT for major depression, panic disorder, or social anxiety disorder - Arabic-speaking patients suffering from an emotional disorder (subset)	Internet-delivered Cognitive Behavioral Therapy (ICBT) with scheduled weekly therapist guidance. The exact duration and dose are not specified.	- ML algorithms predicted treatment success or failure with a balanced accuracy between 56% and 77%, outperforming previous strategies. - Predictive power increased when treatment week data were added to baseline data. - The algorithms outperformed a previous predictive algorithm that included therapist input.
Kálózi-Szabó et al. (2022) [194]	- Report on a pilot study at the intersection of neurodiversity and educational robotics. - Promote the love for classic children’s and young people’s literature. - Develop cognitive and socioemotional abilities of students. - Assess measurable changes in targeted development areas through robotics. - Build a sustainable bridge between technology and education, ensuring Self-Regulated Learning and considering learners’ neurodiversity.	- Sixth-grade students - Aged between 11.25 and 13.08 years (M = 12.16; SD = 0.65) - 9 females out of 22 participants - Study conducted in Budapest, Hungary	RIDE program sessions using ArTec robots, held once per week in school settings, with five modules completed due to COVID-19 lockdowns. The program aimed to develop cognitive skills such as computational thinking, spatial relations, visuo-constructive ability, attention, and reading ability.	- Participants showed significant improvements in visuo-constructive abilities, reading accuracy, and reading comprehension after the robotics program. - The study observed a general improvement in most cognitive measures, although not all were statistically significant. - The RIDE project’s curriculum is considered innovative and inclusive, contributing to its effectiveness.
Ke et al. (2019) [195]	- Explore the effect of tDCS on the variation in performance during WM training in healthy young adults. - Analyze the variation of performance of the stable-load task during multisession load-adaptive WM training with anodal HD-tDCS. - Investigate whether anodal HD-tDCS facilitates the WM training procedure and boosts the training speed of the stable-load task.	- College students - Aged 20–25 - 18 males out of 30 participants - Healthy (no self-reported history of mental or neurological illness and drug abuse) - Normal or corrected to normal vision	Active anodal high-definition transcranial direct current stimulation (HD-tDCS) targeting the left dorsolateral PFC, administered at 1.5 mA for 25 min per session, over 5 consecutive days.	- Active anodal HD-tDCS led to higher learning rates and greater performance improvements during WM training compared to sham stimulation. - The benefits of tDCS were transferable to similar untrained WM tasks. - Individuals with lower baseline performance showed greater training improvements with tDCS.
Keebler et al. (2014) [196]	- Examine the effects of a novel AR guitar learning system (Fretlight^®^) in relation to embodied music cognition theories. - Compare the Fretlight^®^ system to standard instructional materials (diagrams) in terms of learning effects. - Experiment 1: Investigate short-term learning effects and performance differences using Fretlight^®^ versus diagrams (Optek Music Systems, Inc., Reno, NV, USA). - Experiment 2: Examine long-term retention effects of learning with Fretlight^®^ over a two-week interval.	- Undergraduate students - Adults aged 18 and above - Right-handed individuals - No prior formal or informal stringed instrument training - Experiment 1: 30 females, 25 males - Experiment 2: Mean age, 29.83 years; age range, 24–39 years; 2 females, 4 males	Fretlight^®^ guitar with LED lights for finger placement guidance, used for 30 training trials in Experiment 1 and Experiment 2, with a follow-up test after 2 weeks in Experiment 2.	- The Fretlight^®^ guitar system led to significantly better long-term retention and initial learning ease compared to traditional methods. - Participants using the Fretlight^®^ system demonstrated improved performance in early training trials and shorter scale completion times. - Overall, the Fretlight^®^ system provided a lower barrier of entry and enhanced LE for guitar playing.
Kerr et al. (2015) [197]	- Evaluate the efficacy of the Cogmed working memory program in children with epilepsy.	- Children - Have epilepsy	Cogmed computerized working memory program	Not mentioned (the abstract does not provide specific results or conclusions of the study)
Kesler et al. (2017) [198]	To determine if resting state fMRI acquired at pre-treatment baseline could accurately predict breast cancer-related cognitive impairment at long-term follow-up.	- Female - Aged 34–65 - Diagnosed with primary breast cancer - Undergoing or had undergone chemotherapy - Includes a control group of healthy females	1. Doxorubicin, cyclophosphamide, and paclitaxel (16 participants). 2. Cyclophosphamide, doxorubicin, and fluorouracil (2 participants). 3. Cyclophosphamide and paclitaxel (9 participants). 4. Doxorubicin, carboplatin, and paclitaxel (2 participants). 5. Fluorouracil, epirubicin, and cyclophosphamide (2 participants).	- Cognitive impairment at 1 year post chemotherapy was observed in 55% of breast cancer patients. - Resting state fMRI combined with ML accurately predicted cognitive impairment with up to 100% accuracy. - The neuroimaging-based model was significantly more accurate than models using only patient-related and medical variables.
Kirchner et al. (2016) [199]	- Show that P300-related activity is naturally evoked when task messages are presented and recognized. - Investigate modulation of P300 by operator demands and task engagement. - Demonstrate that single-trial detection of P300-related activity can be used to adapt interaction based on task engagement. - Investigate if ISI adaptation can achieve balanced task involvement and optimized performance.	- Male - Aged 20–38 years (mean: 28.74, SD: 6.92) - Normal or corrected to normal vision	Participants received an intervention involving the use of a man–machine interface (MMI) for multi-robot control, which adapted to changes in task load and task engagement based on P300-related brain activity. The intervention included six runs, each with 30 tasks, where EEG was used to monitor brain activity and adjust the inter-stimulus interval (ISI) online in runs 5 and 6 based on P300 detectability.	- The developed MMI significantly improved runtime for interaction tasks by adapting to user needs. - Single-trial detectability of P300 was used to measure task load and engagement, allowing for individual MMI adaptation without increasing workload. - Online adaptation of ISI based on P300 detectability improved user performance by balancing task engagement and avoiding overload.
Kosch et al. (2022) [200]	- Demonstrate a placebo effect in adaptive interfaces. - Investigate how the belief in receiving AI support affects expectations and performance. - Integrate findings into technological acceptance theories. - Explore how system descriptions can alter subjective evaluations and task performance. - Investigate if sham treatments can influence subjective and objective metrics of effectiveness. - Evaluate the placebo effect in Human–AI interfaces.	- 166 female, 80 male, 4 identified with another gender, 1 preferred not to disclose gender - Native speakers from the US and the UK - Participants recruited through Prolific	Not applicable (the paper describes sham treatments as part of a placebo effect study, but no actual interventions were applied).	- Belief in receiving AI support increases participants’ expectations about their own task performance, demonstrating a placebo effect on subjective performance ratings. - The placebo effect does not significantly impact objective performance measures, such as error rates, in task completion. - System descriptions can alter subjective evaluations and expectations without affecting objective task performance.
Lackmann et al. (2021) [201]	- Compare lecture capture and infographic video formats to identify which engages students more emotionally and cognitively over time. - Determine which video format provides better learning outcomes. - Examine the relationship between engagement and learning outcomes.	- 26 participants - 16 males and 10 females - Average age: 26.20 years - Participants were young students - No prior university-level psychology education - Excluded individuals with psychological diagnoses such as epilepsy	1. Infographic video: continuous flow of images, graphics, and text synchronized with an audio track for approximately 14 min. 2. Lecture capture: video recording of a class lecture without additional enrichment for approximately 14 min.	- Infographic videos maintain higher emotional and cognitive engagement over longer periods compared to lecture capture videos. - Infographic videos lead to significantly improved performance on difficult questions. - There is a positive relationship between engagement and performance, with a quadratic relationship observed for cognitive engagement.
Lagoa et al. (2014) [202]	- Introduce adaptive intensive interventions. - Illustrate new methods from control engineering for designing adaptive intensive interventions. - Achieve an approximately 30 percent improvement over mean smoking urge without treatment.	Not mentioned (the paper uses simulated data for methodological illustration and does not provide real-world demographic characteristics)	Adaptive intensive intervention using a smartphone application for smoking cessation, involving self-report data collection three times per day for 50 days. The application includes motivational messages, coping skills improvement, positive reinforcement, and reminders for quitting smoking. Treatment is provided probabilistically (50% chance) at each data collection point.	- Adaptive intensive interventions designed using control engineering methods improve outcomes with less frequent treatment. - These interventions effectively reduce participant burden while maintaining or enhancing effectiveness. - The adaptive intervention achieved a lower smoking urge, negative affect, and higher self-efficacy compared to full treatment.
Lee et al. (2018) [203]	- Use individualized physical maneuvers to create controlled lower and higher pain states in chronic low back pain patients. - Develop multivariate machine learning models using brain imaging and autonomic activity data to predict within-patient and between-patient clinical pain intensity states. - Introduce a model with potential biomarkers for clinical pain, applicable in noncommunicative patients and for identifying objective pain endophenotypes.	- Patients with chronic low back pain (cLBP) - Age range: 18–60 years old - Mean age: 37.37 years - 33 females out of 53 patients - Fluent in English - Able to exacerbate back pain through physical maneuvers - Excluded if they had specific causes of back pain, radicular pain below the knee, complicated back problems, major systemic or neuropsychiatric diseases, substance abuse disorder in the past two years, contraindications to MRI scanning, or high use of prescription opioids/steroids	Individualized physical maneuvers to exacerbate clinical pain, including toe touches, back arches, and facet joint loading twists, performed to increase pain ratings by at least 30%.	- The study developed a machine-learning model that accurately classifies within-patient clinical pain states using combined multimodal neuroimaging and autonomic metrics. - The model successfully predicts between-patient clinical pain ratings, demonstrating potential for clinical applications in noncommunicative patients. - Combining S1 connectivity, regional cerebral blood flow, and high-frequency heart rate variability enhances the model’s predictive accuracy.
Lee et al. (2018) [204]	- To assess the impact of cognitive styles and learning modules using mobile e-learning on knowledge gain, competence gain, and satisfaction for emergent ORL-HNS disorders.	- Undergraduate medical students - Age range: 22–26 years, median age 23 years - Gender: 36 males, 24 females - Novices in ORL-HNS (no previous training in this field) - Participants were in a medical clerkship	Novel interactive multimedia (IM) module on a 7-inch tablet for 100 min, involving interactive elements such as operating a character, interacting with nonplayer characters, and engaging in game-based quizzes.	- Mobile e-learning effectively improves knowledge of emergent ORL-HNS disorders in millennial undergraduate medical students. - Different cognitive styles necessitate the development of various learning modules to optimize educational outcomes. - Students preferred the interactive multimedia module over the PowerPoint show module due to its efficiency and enjoyment.
Leung et al. (2015) [205]	- Examine the behavioral effects of a systematic thirteen-week cognitive training program on attention and working memory in older adults at risk of cognitive decline. - Evaluate the specific cognitive effects of planned experience delivered in a cognitive training program. - Assess the effects of a brain plasticity-based training program on enhancing attention and working memory in older Chinese adults at risk of cognitive decline.	- Community-dwelling older Chinese adults - At risk of cognitive decline (MoCA scores 19–26) - Aged 60 years or above - literate - Normal or corrected-to-normal vision and/or hearing - Right-hand dominant - Normal intelligence (as per TONI-III) - No history of neurological or psychological disorders - No history of substance abuse or alcoholism - Not on antidementia medication - No thyroid dysfunction or vitamin B12 deficiency - Not scoring in the moderate or severe range on HADS depression or anxiety subscales	Cognitive training program modeled after the Brain Fitness Program, consisting of three one-hour sessions per week for thirteen weeks. Participants practiced four out of six cognitive exercises (approximately 15 min each) per session, focusing on attention and working memory.	- Cognitive training improved auditory and visual-spatial attention and working memory in older adults at risk of cognitive decline. - The training effects were specific to the domains targeted by the program, with no significant changes in verbal and visual-spatial memory. - Initial cognitive status did not limit the benefits of training, indicating preserved neural plasticity in older age.
Lin et al. (2017) [206]	- Examine the prospective relationship between an adaptive parasympathetic nervous system response to cognitive stimuli and VSOP training-induced plasticity. - Investigate the role of the parasympathetic nervous system in cognitive and neural improvements resulting from VSOP training in older adults with amnestic mild cognitive impairment.	- Participants with amnestic mild cognitive impairment (aMCI) - Aged 60 years or older - English-speaking - Community-dwelling - 10 participants in the VSOP group (5 male) - 11 participants in the MLA group (6 male) - Recruited from university-affiliated memory clinics - Excluded if actively participating in another cognitive intervention study or on antidepressants/anxiolytics	Vision-based speed of processing (VSOP) training, consisting of five computerized attention tasks (Eye for Detail, Peripheral Challenge, Visual Sweeps, Double Decision, and Target Tracker) for 6 weeks. The tasks automatically adjust in difficulty and speed based on participant performance.	- VSOP training resulted in a significant U-shaped HF-HRV response pattern and decreased striatum–prefrontal connectivity, linked to cognitive improvements. - The study provides evidence of PNS adaptation being associated with cognitive and neural improvements following VSOP training. - Significant group differences were observed in HF-HRV and striatum-prefrontal connectivity, correlating with training-induced cognitive changes.
Liu et al. (2016) [207]	- Use neuroimaging to understand the processes underlying the testing effect. - Provide a theoretical explanation for inconsistent patterns in previous studies on the testing effect. - Explore how the allocation of effort to the re-encoding process during retrieval is influenced by ease of retrieval without feedback. - Manipulate the degree of overlearning/ease of retrieval using multiple tests. - Explore contributions of retrieval and re-encoding processes during tests and restudy processes on subsequent learning.	- Young adults aged approximately 20 years - Nine females out of twenty participants - Students at Carnegie Mellon University - Normal or corrected-to-normal vision	1. STT-T condition: Two tests after initial encoding before a final test on the second day. 2. SST-T condition: One restudy opportunity followed by one test before a final test on the second day. 3. SSS-T condition: Study opportunities on Day 1 and a final test on the second day.	- The study identifies two distinct processes underlying the testing effect: retrieval and re-encoding. - Left PFC activation predicts success after the first correct test but predicts failure after multiple correct recalls, indicating diminishing returns with repeated testing. - Right hemisphere activation consistently predicts successful recall, highlighting its role in retrieval processes.
Lucia et al. (2021) [208]	- Test the effects of cognitive-motor training on athletes’ sport performance and cognitive functions. - Verify whether CM-DT training improves sport performance in basketball players compared to physical training only. - Examine the effects of CM-DT training on behavioral performance during a cognitive DRT and on anticipatory processes associated with pre-stimulus BP and pN components.	- Young male adolescents (mean age 16.6 years) - Semi-professional basketball players - Part of an Under-18 team - from Stella Azzurra Basketball Rome (likely Italian) - Absence of neurological and psychiatric disorders - Not on medication during the study - Right-handed - At least 6 years of formal basketball training	Cognitive-motor dual-task (CM-DT) training using the Witty-SEM system, performed twice a week for 30 min per session, involving six exercises divided into three phases (Activation, Central Phase, and Free Choice), with each exercise lasting 1 or 3 min and repeated twice.	- The experimental group showed significant improvements in sport-specific tests and cognitive test accuracy compared to the control group. - Cognitive-motor training enhanced anticipatory cognitive processes in the PFC, improving proactive inhibition and attention. - The training specifically augmented the pN component, indicating enhanced proactive cognitive control.
Mahncke et al. (2021) [209]	To evaluate the effects of a self-administered computerized cognitive training program on cognitive function in people with a history of mild traumatic brain injury (TBI) and cognitive impairment, compared to active control (computer games).	- People with a history of mild TBI - People with cognitive impairment	Self-administered computerized cognitive training program	- A self-administered computerized cognitive training program significantly improved cognitive function in individuals with mild TBI and cognitive impairment. - The improvements were enduring and superior to those achieved by an active control group using computer games.
Maimon et al. (2022) [210]	- Explore the neural mechanism underlying surgery simulation training. - Test the relationship between cognitive load, skill acquisition, and brain oscillation activity levels. - Track cognitive load neuro-markers using an EEG device during surgical simulator tasks. - Test medical students and interns with no prior experience in surgery simulators or real-life patients. - Compare “online gains” and “offline gains” in skill acquisition.	- Predominantly female (68% in Experiment 1, 63% in Experiment 3) - Mean age around 25–28 years (range 25–36 years) - Healthy - Medical interns or students - Completed 6 years of medical studies (Experiment 1) or in their first to sixth years of study (Experiment 3)	1. Surgery simulator task using Simbionix LAP MENTOR™ (Simbionix USA Corp., Cleveland, OH, USA) involving grasping and clamping blood vessels with laparoscopic arms, conducted over three trials with a 5 min break between trials. 2. Brain activity monitoring using a single-channel EEG device (Aurora by Neurosteer^®^ (Neurosteer Inc., New York, NY, USA)) with a three-electrode patch attached to the forehead during the simulator tasks.	- Participants’ behavioral performance improved with trial repetition, indicating enhanced laparoscopic skills. - Delta and VC9 biomarker activity decreased with trial repetition and better performance, suggesting reduced cognitive load. - VC9 biomarker showed strong correlation with performance, validating its use as a measure of cognitive load during surgical tasks.
Martín-Perea et al. (2020) [211]	- To establish whether ML analysis can be used to quantitatively identify discrete fossiliferous levels in paleontological and archeological sites based on spatial distribution. - To apply and test the method in Batallones-3 and Batallones-10 to determine if deposits are homogeneous or heterogeneous with discrete fossiliferous levels.	- Fossilized remains from the Late Miocene era (ca. 9.1 Ma) - Batallones-3: Predominantly carnivoran fossils - Batallones-10: Predominantly herbivore fossils	ML algorithms for pattern recognition, including: 1. Unsupervised learning using DBSCAN for density-based clustering. 2. Expert-in-the-loop collaborative intelligence for refining clusters. 3. Supervised learning using Support Vector Machines (SVM) and Random Forest (RF) for final model tuning.	- ML algorithms were successfully used to identify three discrete fossiliferous levels in both Batallones-3 and Batallones-10. - The study demonstrates the effectiveness of a hybrid intelligence system in quantitatively identifying fossiliferous levels based on spatial data.
Metzler-Baddeley et al. (2017) [212]	- Explore the neural substrates underpinning adaptive training-induced white matter plasticity on the local level within parietofrontal white matter of the superior longitudinal fasciculus (SLF). - Use non-DT-MRI microstructural indices to investigate the underpinnings of white matter plasticity.	- Healthy adults - Aged 19–40 years - Both males and females	Adaptive working memory training using Cogmed, consisting of computerized exercises of verbal and spatial span tasks, practiced five times per week for 8 weeks (40 sessions, approximately 30 h in total), with task difficulty adjusted based on performance.	- Adaptive working memory training improves working memory capacity and induces microstructural changes in parietofrontal and parahippocampal white matter. - These changes include increases in R1, restricted volume fraction, fractional anisotropy, and reductions in radial diffusivity, particularly in the right dorsolateral superior longitudinal fasciculus and left parahippocampal cingulum. - No significant effects were observed on other cognitive domains beyond working memory capacity improvements.
Mo et al. (2022) [213]	- Explore the internal mechanism of learning under the online mode using CLT. - Assist students in using AL at a personalized video playback speed. - Determine what speed students should choose for their online courses. - Investigate if the learning effect differs by playback speeds and if learning ability influences this effect. - Examine if learners’ cognitive load differs by playback speed. - Explore the correlation between students’ learning effect and cognitive load. - Promote personalized AL under the online learning environment.	- 76 undergraduates - University A in Zhejiang Province - Likely young adults (typical undergraduate age range)	Participants watched instructional videos at one of four different playback speeds (1.0×, 1.25×, 1.5×, or 2×) in a single session.	- Video playback speed significantly influences learning outcomes, with optimal effects at 1.25× and 1.5× speeds. - The best playback speed varies by learning ability: high-level learners perform best at 1.5×, while low-level learners perform best at 1.25×. - Science and engineering students perform better than liberal arts students at 1.5× speed due to differences in cognitive load.
Monlezun et al. (2018) [214]	- Determine if hands-on cooking and nutrition education versus traditional education can improve student competencies and attitudes about providing patients with nutrition education. - Assess if the program can improve students’ own diets. - Evaluate if the program could be scalable.	- Medical trainees - Mean age: 25.71 years (SD 2.91) - 61.43% female - 22.65% had nutrition education prior to medical school - 23.21% adhered to a special diet - 30.27% in clinical years - 25.22% intended to enter a primary care specialty	Hands-on cooking and nutrition education program consisting of 28 h of instruction over 8 classes, with each class including a 0.5 h pre-class lecture video, 1.5 h of hands-on cooking, and a 0.75 h post-class Problem-Based Learning session.	- Hands-on cooking and nutrition education significantly improved medical students’ competencies in nutrition counseling compared to traditional curriculum. - The intervention increased students’ adherence to the Mediterranean Diet. - The program reduced the odds of daily soft drink consumption among trainees.
Montani et al. (2014) [215]	- Develop a novel adaptive videogame to support rehabilitation of attention and executive function impairments in TBI patients. - Focus on design features making the game suitable for brain-damaged patients. - Validate the game with unimpaired participants to ensure activation of desired cognitive functions and assess short training effects.	- Undergraduate students - Aged 19–25 years - Normal or corrected-to-normal vision - Healthy young adults	Playing the adaptive videogame “Labyrinth” for 40 min daily for 14 days (totaling less than 10 h) by healthy young adults.	- The adaptive video game successfully engaged and enhanced attentional control in healthy participants. - Participants showed improved performance in multitasking and task switching, indicating enhanced cognitive flexibility. - The game effectively increased task difficulty over time, demonstrating its potential as a training tool for cognitive skills.
Moore et al. (2017) [216]	- Examine the effect of experimental manipulations of both memory load and pain on tasks sensitive to pain interference. - Investigate the effect of cognitive load on pain-related attentional interference.	- Healthy adult men and women - Recruited from the University of Bath staff and student population - Not currently in pain or with chronic pain conditions - Not taking analgesic medication - Mean age around late 20s to early 30s	Experimental thermal pain induction using a Medoc PATHWAY-Advanced Thermal Stimulator (ATS), with a thermode attached to the right ankle. Temperature starts at 32 °C, increases at 8 °C/second to 1 °C above the pain threshold (maximum 48 °C), oscillates +/−1 °C around the threshold at 8 °C/second for 10 oscillations, and repeats throughout the cognitive tasks.	- Pain-related interference with attention was observed only under high cognitive load conditions for the attention span task. - Cognitive load influences the interruptive effect of pain on attention selectively, affecting some tasks but not others. - The study’s findings were unexpected, as pain did not affect attentional switching or divided attention tasks under high load conditions.
Pat et al. (2022) [217]	- Develop brain-based predictive models for cognitive abilities that are developmentally stable over years during adolescence. - Account for the relationships between cognitive abilities and socio-demographic, psychological, and genetic factors.	- Children aged 9–10 years at baseline and 11–12 years at follow-up - Both male and female participants - Recruited from 21 sites across the United States - Focus on children of European ancestry for genetic analyses - Consideration of socio-demographic factors such as neighborhood safety and school environment	Not applicable (the paper is observational and does not discuss an intervention)	- Brain-based predictive models for cognitive abilities were stable over two years and generalizable across different sites, predicting around 20% of the variance in childhood cognition. - The models accounted for significant variance in childhood cognition due to socio-demographic, psychological, and genetic factors, with mediation proportions of approximately 18.65% and 15.6%, respectively. - The stacked model integrating multiple MRI modalities improved predictive performance over single modalities, demonstrating construct validity according to the RDoC framework.
Perdikis et al. (2018) [218]	- Corroborate the importance and efficacy of mutual learning in motor imagery (MI) brain–computer interface (BCI). - Train two end-users with severe motor impairments using a mutual learning approach to control the Brain Runners BCI application and participate in the Cybathlon BCI race.	- Two male participants - Aged 48 and 30 - Tetraplegic with chronic spinal cord injury (ASIA A) - Wheelchair-bound - No control over lower limbs, limited control over upper limbs - No pacemakers or other implants - Do not suffer from epilepsy or cyber-sickness - Do not require respiratory assistance - Use advanced assistive technology in daily life - P1 has previous MI BCI study experience; P2 is BCI naive	1. Mutual learning approach for BCI control: - Offline open-loop BCI training: approximately twice a week, 40 runs for P1, 15 runs for P2. - Online closed-loop BCI feedback training: approximately twice a week, 12 runs for P1, 19 runs for P2. - Race training: approximately twice a week, 182 runs for P1, 57 runs for P2. - Duration: April-October 2016 for P1 (35 sessions), July–October 2016 for P2 (16 sessions).	- Mutual learning in motor imagery BCI is effective, as demonstrated by strong learning effects at machine, subject, and application levels. - The study provides quantitative evidence of operant subject learning in noninvasive MI BCI training. - The Cybathlon competition results highlight the practical success of the mutual learning approach, with one participant setting a competition record.
Peruyero et al. (2017) [219]	To analyze the effect of three physical education sessions of different intensities (no exercise, predominantly light intensity, and predominantly vigorous intensity) on the inhibition response in adolescents.	- Adolescents aged approximately 16 years - 23 boys and 21 girls - From a large city in Spain - Middle socioeconomic class	1. 5 min warm-up, 20 min of light-to-moderate intensity Zumba dance, and 5 min cool-down. 2. 5 min warm-up, 20 min of moderate-to-vigorous intensity Zumba dance, and 5 min cool-down.	- Vigorous intensity exercise significantly improves cognitive inhibitory control in adolescents compared to light or no exercise. - Moderate-to-vigorous intensity sessions result in higher accuracy in cognitive tasks than light-to-moderate or no exercise sessions. - Exercise intensity is crucial for enhancing cognitive function when session time is maintained within recommended values.
Piette et al. (2016) [220]	- Demonstrate that AI-CBT has pain-related outcomes equivalent to standard telephone CBT. - Document that AI-CBT achieves these outcomes with more efficient use of clinician resources. - Demonstrate the intervention’s impact on proximal outcomes associated with treatment response, including program engagement, pain management skill acquisition, and patients’ likelihood of dropout.	- Veterans - Patients with chronic low back pain	AI-CBT intervention: 1. Weekly hour-long telephone counseling sessions initially for all patients. 2. For responders in the AI-CBT group: - 15 min contact with a therapist. - CBT clinician feedback via interactive voice response calls (IVR). - Personalization based on daily feedback including pedometer step count and CBT skill practice.	Not mentioned (the study is in the start-up phase and no results or conclusions are provided in the abstract).
Pozuelos et al. (2018) [221]	- To test the combined effect of metacognitive scaffolding and computer-based training of executive attention in typically developing preschoolers at the cognitive and brain levels.	- Typically developing preschoolers	Metacognitive scaffolding and computer-based training of executive attention for typically developing preschoolers. Specific details on frequency, duration, or amount are not mentioned.	- Children in the metacognitive group showed larger gains in intelligence compared to regular training and control groups. - Significant increases in an electrophysiological index associated with conflict processing were observed in the metacognitive group. - Changes in conflict-related brain activity predicted intelligence gains in the metacognitive scaffolding group.
Priya et al. (2021) [222]	- To propose ML-Quest, a game that incrementally presents a conceptual overview of three ML concepts: Supervised Learning, Gradient Descent, and K-Nearest Neighbor (KNN) Classification. - To help K-12 students boost their understanding of ML at an early age by applying these concepts in a simulated game world. - To introduce the definition and working of these ML concepts to higher-secondary school students without overwhelming them with complex details.	- Upper-secondary school students	ML-Quest game introduced Supervised Learning, Gradient Descent, and K-Nearest Neighbor (KNN) Classification to 41 upper-secondary school students. Duration and frequency not specified.	- Students who played ML-Quest performed better in tests than those who did not, with 5% scoring full marks. - 77% of participants agreed that ML-Quest made learning interactive and helpful for understanding ML concepts. - The game was perceived as easy to use, useful, and correctly aligned with ML concepts, with high satisfaction scores from participants.
Rafique et al. (2021) [223]	- Develop a system to predict students’ performance and help teachers introduce corrective interventions. - Explore the potential of collaborative learning as an intervention to improve student performance.	Students	Collaborative learning (specific details on frequency, duration, and implementation not provided)	- A system was developed to predict student performance and enable timely interventions to improve low-performing students’ outcomes. - Collaborative learning methods significantly enhance students’ learning capabilities. - Students who actively participated in class activities and completed tasks performed better than others.
Rahman et al. (2022) [224]	- To determine if adding neural measures to a behaviorally adaptive training system improves human performance on a transfer task. - To measure the effectiveness of adding neural measures within a closed-loop BCI into adaptive training.	- Healthy adults - Screened for normal or corrected-to-normal vision - Excluded if they had a tendency for motion sickness or brain-related diseases - Participants included both males and females - Mean age around 29–33 years	1. Behaviorally Adaptive Training (BAT): Task difficulty varied based on behavioral performance, involving 600 trials over 20 blocks, with each trial lasting 1.5 s and inter-trial intervals of 1.0–2.0 s. 2. Combined Adaptive Training (CAT): Task difficulty varied based on both behavioral performance and EEG measures, involving 600 trials over 20 blocks, with each trial lasting 1.5 s and inter-trial intervals of 1.0–2.0 s.	- The Combined Adaptive Training (CAT) system, which integrates neural and behavioral measures, significantly improves transfer task performance compared to Single Item Fixed Difficulty (SIFD) and Behaviorally Adaptive Training (BAT) systems. - CAT showed a 6% improvement over SIFD and a 9% improvement over BAT in transfer task performance. - The integration of neural measures into adaptive training systems enhances learning outcomes beyond what is achieved with behavioral measures alone.
Renn et al. (2021) [225]	- Assess the feasibility of an intelligent tutoring system (ITS) as a classroom adjunct. - Assess the acceptability of an ITS as a classroom adjunct. - Assess the effectiveness of an ITS as a classroom adjunct to improve training BSW students in client engagement strategies.	- Undergraduate students in a Bachelor of Social Work program	Intelligent Tutoring System (ITS) as a classroom adjunct for training in client engagement strategies for BSW students enrolled in a class on telephone-based cognitive behavioral therapy (tCBT). Frequency, duration, and amount not specified.	- The intelligent tutoring system (ITS) was effective, with 81.8% of students in Wave 1 and all students in Wave 2 passing the clinical skills role-play. - Students who progressed more quickly through the ITS had better competency ratings in tCBT. - The ITS has the potential to streamline and scale training in evidence-based practices.
Rennie et al. (2019) [226]	- Use unsupervised ML techniques to identify differential trajectories of change in children undergoing working memory training. - Provide an alternative approach to analyzing cognitive training data beyond individual task changes. - Establish a method for identifying data-driven subgroups with distinct cognitive profiles. - Demonstrate a proof of principle for exploring multivariate profiles of change.	- Children aged 5.16–17.91 years - Mean age around 9 years - Typically developing - Attending mainstream schools in the UK - Includes both boys and girls (45 girls mentioned in one sample)	Intensive working memory (WM) training using tasks from the Automated Working Memory Assessment (AWMA), including Forward Digit Recall and Backward Digit Recall tasks. Specific frequency, duration, and amount of training are not mentioned.	- Unsupervised ML techniques revealed differential trajectories of change in children undergoing working memory training. - Fluid intelligence scores were predictive of children’s improvement trajectories, indicating individual differences in response to training. - Task relationships changed following training, suggesting that cognitive processes involved in tasks may become more task-specific rather than domain-general.
Ristić et al. (2023) [227]	- To determine if the adaptive e-learning model provides a higher degree of knowledge and positively influences knowledge duration compared to a standard non-adaptive e-learning system. - To assess if the adaptive e-learning model increases students’ learning motivation compared to a standard non-adaptive e-learning system. - To explore the relationship between learning styles and achievement scores on A1, A2, S1, and S2 tests. - To investigate if there is a statistically significant difference between gender, learning motivation, achievement scores on tests, and satisfaction with the adaptive e-learning system.	- 228 students - Third-year undergraduate students - Likely aged around 20–22 years - Studying at the Faculty of Management in Serbia	Adaptive e-learning system based on the VAK learning style model, implemented in Moodle, used for six chapters during the second half of a semester for 228 students.	- Adaptive e-learning systems significantly improve learning effectiveness, satisfaction, and motivation compared to standard e-learning systems. - Students achieve better test results after using adaptive e-learning modules, indicating enhanced academic performance. - There is a significant relationship between adaptive e-learning systems and increased student motivation, which is not observed with standard systems.
Roberts et al. (2016) [228]	- To test whether a computerized adaptive working memory intervention program improves long-term academic outcomes of children 6 to 7 years of age with low working memory compared with usual classroom teaching. - To determine the efficacy of Cogmed on reading, spelling, and mathematics at 12 and 24 months. - To evaluate the costs and benefits of the intervention.	- Children aged 6 to 7 years - From Melbourne, Australia - Attending government, Catholic, and independent schools - Majority have parents with tertiary education - Likely English-speaking families	Cogmed working memory training, comprising 20 to 25 training sessions of 45 min’ duration at school over 5 to 7 weeks. Each session involved children using computers and noise-canceling headphones to complete adaptive tasks, with a new task introduced every 5 to 6 days.	- The Cogmed intervention provided a temporary benefit to visuospatial short-term memory but did not improve long-term academic outcomes in reading, spelling, or mathematics. - The intervention was not cost-effective due to high costs and loss of classroom time without lasting benefits. - The study does not recommend the population-based delivery of Cogmed within a screening paradigm due to the lack of sustained academic improvement.
Roelle et al. (2015) [229]	- Test the active < constructive learning hypothesis. - Test different types of prompts to induce interactive learning activities. - Test the constructive < interactive learning hypothesis.	- Eighth-grade students aged 13–15 - Eleventh-grade students aged 16–19 - Participants from a German high-track secondary school (Gymnasium) - Gender distribution: Experiment 1—47 females, 36 males; Experiment 2—28 females, 12 males	1. Complete explanations with engaging prompts (active condition) for 12 concepts. 2. Reduced explanations with inference prompts (constructive condition) for 12 concepts. 3. Reduced explanations with inference prompts and adapted remedial explanations with engaging prompts (interactive/engaging prompts condition) for 12 concepts. 4. Reduced explanations with inference prompts and adapted remedial explanations with revision prompts (interactive/revision prompts condition) for 12 concepts.	- Inference prompts were more effective than engaging prompts in enhancing conceptual knowledge, supporting the active < constructive learning hypothesis. - Revision prompts were more effective than engaging prompts in eliciting interactive learning activities during remedial explanations. - Learners in the interactive/revision prompts condition acquired more conceptual knowledge than those in the constructive condition, supporting the constructive < interactive learning hypothesis.
Ruiz et al. (2019) [230]	- Investigate the relationship between instruction, individual differences in cognitive abilities (working memory and declarative memory), and second language vocabulary acquisition in a web-based ICALL context. - Examine whether individual differences in working memory and declarative memory predict L2 lexical acquisition. - Explore how these cognitive abilities interact with different instructional conditions (meaning-focused vs. form-focused). - Focus on the lexical learning of English phrasal verbs. - Determine if an ICALL system can be used for large-scale experimental research on these topics.	- Adult learners of English - Mean age: 24.3 years - Predominantly female (91 women) - Majority native German speakers (78%) - Advanced-level English proficiency (61% advanced learners)	1. Form-focused instruction: Reading news texts and completing automatically generated multiple-choice gaps where phrasal verbs appeared, for about two weeks. 2. Meaning-focused instruction: Reading news texts without completing any gaps, for about two weeks.	- Working memory is a significant predictor of vocabulary acquisition in form-focused instructional conditions, indicating an aptitude-treatment interaction. - Declarative memory, particularly nonverbal, is associated with vocabulary learning, but its effect is not moderated by instructional condition. - The study underscores the role of individual cognitive differences in second language vocabulary acquisition within ICALL environments.
Sánchez-Pérez et al. (2018) [231]	- Examine the efficacy of a school computer-based training program composed of working memory and mathematics tasks. - Evaluate the effects of the training on children’s cognitive skills, including executive functions (EF) and IQ. - Assess the impact of the training on children’s academic achievement in math and language.	- Primary school children	Computer-based training program consisting of: 1. Working memory tasks with varying difficulty levels (1-, 2-, 3-back) and presentation times (500 ms or 1000 ms). 2. Mathematics tasks requiring solving mathematical operations, numerical sequences, and problems within a time limit of two minutes.	- The study found significant improvements in cognitive skills, such as non-verbal IQ and inhibition, and better school performance in math and reading among children who participated in the training. - Most of the improvements were attributed to training on working memory tasks. - The computer-based training program combining working memory and mathematics activities was confirmed to be effective in enhancing children’s academic competences and cognitive skills.
Sánchez-Pérez et al. (2019) [232]	- Investigate the effectiveness of computer-based cognitive training on improving cognitive and academic-related skills in primary school-aged children. - Examine brain functional connectivity effects induced by WM-based training, specifically in the MFG area of the attentional networks and the frontoparietal network. - Investigate the relationship between improvements in inhibition-related abilities and changes in brain functional connectivity.	- Primary school-aged children - Mean age approximately 9 years - Training group: 19 boys, 14 girls; control group: 15 boys, 8 girls - Significant differences in socioeconomic status (SES) between groups - Likely Spanish or residing in Spain	Computer-based cognitive training program focused on working memory tasks, integrated into school routine, supervised by trained teachers, with personalized difficulty levels. Duration and frequency not specified.	- A computer-based cognitive training program improved cognitive and academic skills, including inhibition skills, non-verbal IQ, mathematics, and reading skills, in primary school-aged children. - The training led to increased functional connectivity in the right middle frontal gyrus (MFG), associated with improvements in inhibitory control. - Connectivity between the r-MFG and homolateral parietal and superior temporal areas was more strongly related to inhibitory control improvement in trained children compared to the control group.
Scharnowski et al. (2014) [233]	- Assess changes in effective brain connectivity associated with neurofeedback training of visual cortex activity. - Investigate the neural underpinnings of successful self-regulation using dynamic causal modeling (DCM). - Characterize effective connectivity changes between the trained visual ROI and the cSPL.	- 16 human volunteers - Ages between 18 and 37 years - 6 male participants - All right-handed - Normal or corrected-to-normal vision	Neurofeedback training using real-time fMRI, conducted over at least three sessions with each session including an average of two training runs lasting 8.3 min each. Each training run consisted of seven 38 s baseline blocks interleaved with up-regulation blocks of the same duration. Feedback was provided via a thermometer display indicating signal change.	- Neurofeedback training increased effective connectivity between the visual cortex and the superior parietal lobe. - Successful training was characterized by enhanced top-down control from the superior parietal lobe to the visual cortex and reduced bottom-up processing. - The connectivity changes were associated with the use of visual-spatial attention and imagery as cognitive strategies.
Schlatter et al. (2022) [234]	- To evaluate the effectiveness of two types of adaptive instruction (macro-adaptive and micro-adaptive) in teaching scientific reasoning to children. - To compare these adaptive methods against a non-adaptive control condition in terms of learning outcomes.	- Children	1. Macro-adaptive instruction based on standardized test scores (*n* = 58). 2. Micro-adaptive instruction based on performance in the previous lesson (*n* = 46).	- All three instructional conditions (macro-adaptive, micro-adaptive, and non-adaptive) led to comparable improvements in pre- and post-test scores. - Children’s task performance improved during the lessons, with this improvement interacting with the instructional condition. - More specific information and frequent adaptations might lead to better learning outcomes, suggesting potential for future research in hybrid solutions.
Shangguan et al. (2020) [235]	- Examine the impacts of visual and behavioral emotional design on emotional, motivational, and cognitive outcomes of middle school students. - Investigate the multi-leveled effects of emotional design (visual and behavioral) on multimedia learning. - Explore the effects of emotional design on positive emotions, cognitive load, motivation, and learning outcomes with a new learning topic.	- Middle school students - Ages 13–16 - Gender distribution: Experiment 1—29 males, 21 females; Experiment 2—79 males, 94 females - Normal or corrected-to-normal vision	1. Visual positive emotional design: colorful and anthropomorphic elements in learning materials. 2. Behavioral positive emotional design: self-control of learning progress by fast-forwarding, rewinding, or repeating the learning video.	- Both visual and behavioral emotional designs positively affect learners’ emotions, enhancing the emotional experience during learning. - Visual positive emotional design increases mental effort and perceived task difficulty, indicating a complex interaction with cognitive processes. - Combining visual and behavioral emotional designs improves learning performance, particularly in terms of transfer scores.
Singh et al. (2022) [236]	- Predict participants’ daily adherence to cognitive training programs based on previous adherence patterns. - Understand long-term adherence barriers and develop algorithms to predict and prevent adherence failures. - Use multivariate time series data to determine if participants will meet minimum adherence criteria.	- Older adults with a mean age of 72.6 years - 66% female, 32% male, 2% unspecified gender - Mean age for females: 71.5 years - Mean age for males: 75.0 years - Participants had varying levels of technology proficiency (average MDPQ score: 27.10)	Gamified cognitive training tasks using the Mind Frontier software (Aptima, Inc., Woburn, MA, USA) for 45 min per day, 5 days a week, for 12 weeks.	- Deep learning models, including CNN, LSTM, and CNN-LSTM, effectively predicted older adults’ adherence to cognitive training programs with high F-scores. - Data augmentation techniques significantly improved the generalization performance of the predictive models.
Stiller et al. (2019) [237]	- Investigate the effects of game play and expertise in gaming and English on motivation, cognitive load, and performance. - Compare the effects of an educational game with a traditional text-based hypertext instruction on motivation, cognitive load, and performance.	- German university students - Mixed gender (majority female: 30 out of 39) - Recruited from Regensburg University and East Bavarian Technical University of Regensburg - Less experienced in e-learning environments and educational games - Little prior knowledge about cell biology	Playing the educational game “CellCraft” for 1 h.	- Educational games increased motivation but did not lead to superior performance compared to hypertext learning. - Cognitive load patterns differed between groups, but overall levels were similar. - The hypertext group showed greater knowledge gains than the gaming group.
Sun et al. (2022) [238]	- Design and test a brain–machine interface (BMI) that combines automated pain detection with treatment in freely behaving rats. - Accurately detect and treat acute evoked pain and chronic pain using the BMI.	- rats	Optogenetic activation or electrical deep brain stimulation (DBS) of the PFC in freely behaving rats, as part of a closed-loop brain–machine interface for on-demand pain relief. Frequency, duration, and dose are not specified.	- A multi-region brain–machine interface accurately detected and treated acute and chronic pain in rats. - The analgesic effects of the brain–machine interface were stable over time. - The study suggests that brain–machine interface approaches might be effective for treating chronic pain of different etiologies.
Tacchino et al. (2015) [239]	- Describe the design of Cognitive Training Kit (COGNI-TRAcK), an app for mobile devices for at-home cognitive rehabilitation. - Test the disposability-to-use (usability, motivation to use, compliance to treatment) of COGNI-TRAcK on cognitive-impaired patients with multiple sclerosis.	- Patients with multiple sclerosis (MS) - Mean age: 49.06 years (range 33–67) - Gender: 3 men and 13 women - Recruited from the AISM Rehabilitation Center, Genoa, Italy - Forms of MS: 9 relapsing-remitting, 7 progressive - Mean education: 11.75 years - Mean EDSS score: 3.75 - Mean disease duration: 161.69 months - Familiar with electronic devices	8-week at-home intervention using the COGNI-TRAcK app, consisting of 5 daily scheduled 30 min sessions per week, each including three types of working memory exercises (Vs-WM, Op-NB, and D-NB) for about 10 min each.	- The COGNI-TRAcK app demonstrated high adherence (84%) among patients with multiple sclerosis, indicating consistent use. - The app was well-received, with most patients finding it easy to use, interesting, and useful for cognitive rehabilitation. - Patients reported high motivation and low stress levels during the use of the app, suggesting it is a viable tool for at-home cognitive training.
Taxipulati et al. (2021) [240]	- To determine if different types of feedback affect problem-solving performance in the test stage. - To assess the impact of feedback types on subjective cognitive load and objective eye-tracking trajectory. - To evaluate the effect of feedback types on learner motivation. - To explore if cognitive load and motivation mediate the effect of feedback on problem-solving performance.	- Undergraduate and graduate students - Aged 17–26 - 38 men and 119 women - From Northeast Normal University	1. Knowledge of Correct Response (KCR) feedback: question stem + correct answer. 2. Elaborated Feedback (EF): question stem + correct answer + correct answer resolution. 3. Adaptive Feedback (AF): if the answer is correct, provide KCR feedback; if the answer is wrong, provide EF. 4. Immediate feedback: provided after each problem. 5. Delayed feedback: provided after all problems are completed.	- Immediate feedback results in higher academic performance compared to delayed feedback. - Adaptive feedback improves academic performance more than elaborated and knowledge of correct response feedback. - Germane Cognitive Load (GCL) partially mediates the effect of adaptive feedback on academic performance.
Tee et al. (2023) [241]	- To evaluate the efficacy of an Artificial Intelligence-driven tutoring system in providing risk-free training and objective assessment to improve surgical technical skills. - To compare the teaching efficacy of the AI-driven system with human instructor training.	- Medical students	1. Real-time intelligent instruction: Tutoring by an AI system with error clips and expert-level demonstrations after each repetition. 2. Real-time human instruction: Tutoring by human instructors with critiques and demonstrated correction techniques after each repetition.	- The AI-driven tutoring system led to significant performance improvements in surgical skills training, with higher scores than human instruction by the fifth repetition. - The AI system resulted in higher extrinsic cognitive load compared to human instruction, indicating more efficient learning. - No significant differences were found between the groups for intrinsic and GCL.
Tremblay et al. (2022) [242]	- Determine the impact of modulating task and environment complexity on novices’ performance in simulation. - Determine the impact of modulating task and environment complexity on novices’ cognitive load in simulation. - Determine the impact of modulating task and environment complexity on novices’ knowledge in simulation.	- Second-year undergraduate pharmacy students - Mostly female (72%) - Mean age of 22 years (SD = 2.5) - College degree prior to PharmD program (78%) - Limited clinical experience (3 to 4 weeks of internship) - 27% had prior real-life experience with similar cases	Students participated in a simulation-based education intervention where they acted as pharmacists in one of four conditions: simple task in simple environment, complex task in simple environment, simple task in complex environment, or complex task in complex environment. Each student participated in one session comprising three different learning tasks, playing the pharmacist role once. The intervention was designed to vary task complexity (number of information elements and interactions) and environment complexity (orderliness and visual load).	- Task and environment complexity interact to affect novices’ performance, with simple task performance decreasing in complex environments. - Task complexity significantly impacts ICL, but environmental complexity does not. - Increasing complexity does not necessarily lead to cognitive overload or reduced performance in simulation-based education.
Tuti et al. (2019) [243]	- Determine the effectiveness of offering adaptive versus standard feedback on the learning gains of clinicians using a smartphone-based game. - Examine the effects of learner characteristics and learning spacing on individualized normalized learning gain.	- Clinicians providing bedside neonatal care - Participants from low-income countries - Healthcare providers in training or actively practicing - Excludes retired clinicians and those from high-income settings - Varied socioeconomic status, with some facing financial constraints	Adaptive differentiated immediate feedback provided through a smartphone-based serious gaming app, tailored to individual performance during learning tasks. Only participants in the experimental group received this intervention. The frequency and duration are not specified as they depend on user interaction with the app.	- Adaptive feedback had a small and statistically insignificant effect on learning gains at the group level. - When controlling for individual learner characteristics, adaptive feedback significantly increased learning gains with immediate repetition. - Spaced learning of a week or more significantly reduced learning gains.
Tzachrista et al. (2023) [244]	- To provide an in-depth review of the neurocognitive aspects of creativity and its association with academic achievement in children. - To examine and provide answers to the following research questions: - What are the cognitive processes associated with creative thinking? - How does creativity affect academic performance? - What are the differences in the neurocognitive profiles of creative and noncreative students? - How can creativity be fostered in the classroom? - How does creativity interact with other cognitive processes to affect academic performance?	- Students aged 5 to 16 - Normal, typical development (no diagnosed developmental disorders) - Attending public schools	1. 20 min Hatha yoga sessions. 2. Cooperative high-intensity interval training.	- There is a significant positive relationship between creativity and academic performance, particularly in reading, comprehension, and writing tasks. - Neurocognitive processes like associative thinking, divergent thinking, executive functions, and predictive representations are crucial in shaping creativity. - The study advocates for fostering creativity in educational settings through cultural resources and aligning teachers’ attitudes with creativity promotion.
Varga et al. (2019) [245]	- Identify the cognitive factors contributing to individual differences in self-derivation and retention of knowledge through memory integration in adults. - Test whether a similar profile of cognitive correlates is observed in 8-to 10-year-old children. - Examine whether self-derivation through memory integration is associated with concurrent and longitudinal academic success.	- Adults aged 18–24 - Majority female (63 out of 117) - Racial composition: 9% African American, 25% Asian, 59% Caucasian, 4% mixed racial descent - 8% Hispanic ethnicity - Undergraduate students at a private institution - Children aged 8–10 (third grade) - Racial composition: 45% African American, 40% Caucasian non-Hispanic, 11% Caucasian Hispanic - Attending a rural public school in the southeastern United States - Approximately 87% qualify for federally funded school lunch assistance	Participants read 60 sentences (30 pairs of stem facts) over two sessions spaced one week apart and were tested for self-derivation of 30 possible integration facts. They also completed standardized cognitive assessments in short-term memory, relational reasoning, working memory, long-term memory retrieval, reading comprehension, and verbal knowledge.	- Verbal knowledge and skills are significant predictors of self-derivation performance in both adults and children. - Working memory predicts self-derivation performance in adults but not in children. - Self-derivation through memory integration is associated with concurrent and longitudinal academic success.
Veen et al. (2016) [246]	- To test whether recall of an aversive autobiographical memory loads working memory (WM) compared to no recall. - To test whether recall with eye movements (EM) reduces the vividness, emotionality, and cognitive load of recalling the memory more than only recall or cognitive effort (i.e., recall of an irrelevant memory with EM).	- Undergraduates	Recall relevant memory with eye movements (EM) for 8 × 24 s and 16 × 24 s.	- Recalling an aversive memory with eye movements reduces its vividness and emotionality more than recalling without eye movements or an irrelevant memory with eye movements. - The cognitive load of recalling the memory decreases with eye movements, but not consistently more than in control conditions. - Recalling an aversive autobiographical memory is a cognitively demanding task.
Vekety et al. (2022) [247]	- Examine the effects of mindfulness training supplemented with EEG feedback on objective measures of executive functions and brain activity correlates in elementary school children. - Explore the potential effects of a mindfulness program with EEG feedback to empower children’s attention regulation, measured through neurocognitive tests and brain activity.	- Children aged 9–10 years - Recruited from a local primary school in Budapest, Hungary - From families of middle and high socioeconomic status - Gender distribution: 51% girls, 49% boys - Generally healthy (children with psychological disorders were excluded)	Mindfulness training with EEG feedback using a Muse brain-sensing headband was conducted over eight sessions in 4 weeks. Session durations were: 1 min for the first two sessions, 2 min for the third and fourth sessions, 3 min for the fifth and sixth sessions, and 4 min for the seventh and eighth sessions. The intervention included mindful breathing exercises with auditory feedback.	- Mindfulness training with EEG feedback significantly improved children’s inhibition and information processing compared to a control group. - The intervention maintained alpha and theta brain activity levels, which decreased in the control group, indicating a stabilizing effect on brain function. - These findings suggest potential benefits for children’s Self-Regulated Learning and academic achievements.
Venkat et al. (2020) [248]	- Assist teachers in the health professions in improving their learners’ experiences using tenets of CLT. - Assess the efficacy of the workshop. - Understand how teachers would envision CLT as applying to their workplace teaching settings. - Consider challenges and barriers they might face as they attempt to implement those changes. - Assess whether educators can independently craft plans for improving their teaching practices based on CLT.	- Health professions educators - Majority female (78%) - Primarily from medicine, nursing, education, and dentistry	A 2 h workshop on CLT for health professions’ workplace educators, including large-group didactics, small-group discussions, and individual reflective activities. The workshop was structured as follows: 0–30 min for a large-group overview of CLT, 30–50 min for a small-group activity using a worked example, 50–60 min for a group discussion, 60–80 min for an individual activity to apply CLT principles, and 80–120 min for sharing results and debriefing.	- The workshop increased participants’ self-assessed familiarity with CLT from a mean of 36 to 59. - Participants demonstrated retention of CLT concepts with an average score of 85% on content knowledge questions. - Approximately half of the participants planned or implemented changes in their teaching practices based on CLT principles.
Vidanaralage et al. (2022) [249]	- Explore schema congruent and incongruent participants’ behavior in video-based learning within a flipped learning environment. - Examine the emotional valency of participants during the study and test phases using AI-based emotion analysis.	- Healthy young adults - Aged 20–34 years - Mean age 27.31 years - 9 males (56.25%) - 7 females (43.75%)	Participants watched an educational learning video on a given topic.	- Retrieval accuracy was better for the schema incongruent group compared to the schema congruent group. - Response time was quicker for the schema congruent group than for the schema incongruent group. - Both groups showed more negative emotions during the study and more positive emotions during the test phase.
Wiest et al. (2022) [250]	- Examine the effectiveness of cognitive training for students diagnosed with ADHD and SLD. - Examine the underlying structure among the included cognitive abilities.	- Children and adolescents aged 6–17 (mean age 11.7) - 22 males and 21 females - Diagnosed with ADHD - Diagnosed with specific learning disorders (26 with reading SLD, 6 with writing SLD, 4 with math SLD) - Received psychoeducational evaluations due to educational concerns	Cognitive training via the Captain’s Log program, consisting of 20 sessions, each lasting 60 min, conducted over 4–8 weeks in a clinical setting with supervision by a psychometrician or staff member.	- Cognitive training significantly improved attention, working memory, and inhibition in children with ADHD and SLD. - The study demonstrated structural changes in cognitive abilities following 20 h of computer-based intervention.
Winn et al. (2019) [251]	- Develop an interactive workshop to teach five cognitive learning strategies to pediatric educators. - Provide a platform for educators to understand, apply, and incorporate these strategies into their teaching. - Evaluate the workshop’s effectiveness in promoting behavioral changes in teaching practices.	- Pediatric residents - Chief residents - Fellows - Junior attending faculty members - Senior attending faculty members - Residency program directors - Fellowship program directors - Associate residency program directors - Associate fellowship program directors	A 90 min interactive workshop teaching five cognitive learning strategies: spaced retrieval practice, interleaving, elaboration, generation, and reflection. Each strategy was taught in a 10 min session, including a review, interactive activities, and a brainstorming session.	- The workshop led to behavioral changes in teaching practices, with 82% of participants implementing changes based on the workshop. - Participants initially committed to using interleaving and reflection but eventually applied all five cognitive learning strategies. - The commitment-to-change evaluation strategy confirmed that participants gained and applied knowledge and skills from the workshop.
Yilmaz et al. (2023) [252]	To compare learning efficacy by a real-time intelligent instruction system with in-person human instructor-mediated training.	- Medical students	1. Real-time intelligent instruction by an AI system during five virtually simulated brain tumor resections. 2. In-person human instruction during five virtually simulated brain tumor resections.	- Both AI-based and human instruction significantly improved surgical skills compared to baseline. - AI-based instruction led to higher performance scores than human instruction by the final task. - AI systems can enhance LE with objective, real-time feedback.
Yilmaz et al. (2023) [253]	To compare a real-time intelligent tutoring system in technical skills learning with human expert instructor-mediated training.	- Medical students	1. Real-time intelligent instruction during simulated surgical tasks (Group 2). 2. In-person human instruction during simulated surgical tasks (Group 3).	- Both AI and human instruction significantly improved surgical skills from baseline. - The AI-instructed group outperformed the human-instructed group by the fifth repetition. - AI systems may offer equally or more efficient learning compared to human instruction.
Yin et al. (2020) [254]	- Investigate the impact of a chatbot-based micro-learning system on students’ learning motivation. - Investigate the impact of a chatbot-based micro-learning system on students’ performance.	- First-year students	Chatbot-based micro-learning system (applied to the chatbot-based micro-learning group)	- Students in both chatbot-based and traditional learning environments achieved comparable performance, indicating effective independent learning in the chatbot environment. - Chatbot-based learning significantly increased students’ intrinsic motivation compared to traditional learning, with perceived choice and value as key factors. - Students with high initial perceived choice benefit more from chatbot-based learning, while those with low initial perceived choice benefit more from traditional learning.
Yoon et al. (2022) [255]	- Examine the effects of segmentation and self-explanation designs on ICL. - Examine the effects of segmentation and self-explanation designs on ECL. - Examine the effects of segmentation and self-explanation designs on GCL. - Explore students’ perspectives on segmentation and self-explanation designs in instructional videos.	- Undergraduate students - 32 males and 89 females - From a large public university in the southeastern United States - Includes both education and non-education majors - Includes freshmen, sophomores, juniors, seniors, and fifth-year students	1. Segmentation: Video divided into six segments, allowing control of pacing, applied once during a 7 min and 45 s video session. 2. Self-explanation: Seven open-ended prompts provided to guide self-explanation, applied once during a 7 min and 45 s video session. 3. Combination: Both segmentation and self-explanation prompts applied once during a 7 min and 45 s video session.	- Segmentation in instructional videos resulted in significantly less GCL compared to non-segmenting designs. - Self-explanation did not significantly outperform the control in GCL but was better than segmentation. - Combining segmentation and self-explanation did not enhance GCL compared to using them separately or the control.
Yu et al. (2015) [256]	Explore the effect of visual stimuli degradation on cognitive workload.	Not mentioned (no information on population characteristics is included in the abstract)	Degradation of visual stimuli.	- Degradation of visual stimuli increases cognitive workload, confirmed by subjective assessments and P300 amplitude attenuation. - A single-trial EEG/ERP detection method achieved 85% accuracy in identifying four workload levels. - Frontal EEG signals carry information useful for differentiating workload levels.

**Table 3:** Comparison of CLT, SRL, and PBL in AI-driven learning environments.

Learning Theory	Strengths	Weaknesses	AI Integration Potential	Empirical Evidence
Cognitive Load Theory (CLT)	Optimizes cognitive efficiency by reducing extraneous load; improves retention through structured instructional design.	Overemphasis on reducing cognitive load may limit engagement with complex tasks; assumes one-size-fits-all instructional pacing.	AI-driven cognitive load monitoring and adaptive feedback can dynamically adjust content complexity in real time.	Two studies found: AI-enhanced CLT-based instruction improved comprehension and retention (e.g., video playback speed optimization).
Self-Regulated Learning (SRL)	Encourages learner autonomy, metacognitive awareness, and self-directed skill development.	Highly dependent on learner motivation and self-discipline; may require structured guidance to be effective.	AI-enhanced learning analytics can track learner engagement and provide personalized feedback to optimize self-regulation.	One study found: AI-driven robotics-based SRL programs improved reading accuracy and cognitive abilities.
Problem-Based Learning (PBL)	Fosters real-world problem-solving, critical thinking, and application-based learning.	High cognitive load can overwhelm learners without adequate scaffolding; effectiveness depends on problem authenticity and learner readiness.	AI-based simulations, intelligent tutors, and problem-solving recommendation engines can adjust PBL task complexity dynamically based on real-time learner performance, bridging cognitive gaps effectively.	Four studies found: AI-driven PBL was applied in various fields, including medical training, law education, and STEM; AI-enhanced scaffolding improved learner decision-making speed by 18% and conceptual application by 22%.

## Abbreviations

Artificial Intelligence and Machine Learning	
AI	Artificial Intelligence
AI-CLT	Artificial Intelligence and Cognitive Load Theory
ML	Machine Learning
GAI	Generative Artificial Intelligence
ITS	Intelligent Tutoring System
PGRL	Physics-Guided Reinforcement Learning
SVM	Support Vector Machine
CNN	Convolutional Neural Network
RNN	Recurrent Neural Network
Cognitive and Educational Theories	
CLT	Cognitive Load Theory
ICL	Intrinsic Cognitive Load
ECL	Extraneous Cognitive Load
GCL	Germane Cognitive Load
LE	Learning Efficacy
PL	Personalized Learning
AL	Adaptive Learning
PBL	Problem-Based Learning
SRL	Self-Regulated Learning
EdNeuro	Educational Neuroscience
ZPD	Zone of Proximal Development
SLD	Specific Learning Disabilities
STEAM	Science, Technology, Engineering, Arts, and Mathematics
STEM	Science, Technology, Engineering, and Mathematics
Neuroscience and Brain Monitoring	
EEG	Electroencephalography
fNIRS	Functional Near-Infrared Spectroscopy
PFC	Prefrontal Cortex
tRNS	Transcranial Random Noise Stimulation
MRI	Magnetic Resonance Imaging
P300	Event-Related Potential Component in EEG Research
GSR	Galvanic Skin Response
HRV	Heart Rate Variability
ECG	Electrocardiogram
Educational Technology and Online Learning	
LMS	Learning Management System
ICBT	Internet-delivered Cognitive Behavioral Therapy
K-12	Kindergarten through 12th Grade Education
ICT	Information and Communication Technology
COGWEB	Cognitive Web-Based Training System
CALL	Computer-Assisted Language Learning
Research Methodologies and Statistical Analysis	
PRISMA	Preferred Reporting Items for Systematic Reviews and Meta-Analyses
RCT	Randomized Controlled Trial
RoB 2	Risk of Bias 2 Tool
ANOVA	Analysis of Variance
AIC	Akaike Information Criterion
BIC	Bayesian Information Criterion
CI	Confidence Interval
RMSE	Root Mean Square Error
RMSEA	Root Mean Square Error of Approximation
SRMR	Standardized Root Mean Square Residual
ICC	Intraclass Correlation Coefficient
OSF	Open Science Framework
Psychological and Medical Terms	
ADHD	Attention Deficit/Hyperactivity Disorder
CBT	Cognitive Behavioral Therapy
TBI	Traumatic Brain Injury
Additional Abbreviations Found in the Manuscript	
BCI	Brain–Computer Interface
PASAT	Paced Auditory Serial Addition Task
VSOP	Vision-Based Speed of Processing Training
TOI	Theory of Intelligence
MTBF	Mean Time Between Failures
MTTR	Mean Time To Repair
HR	Heart Rate
AUC	Area Under the Curve
NPV	Negative Predictive Value
PPV	Positive Predictive Value
SCN	Suprachiasmatic Nucleus
TB	Tuberculosis
TEI	Trait Emotional Intelligence
TFL	TensorFlow
VUCA	Volatility, Uncertainty, Complexity, and Ambiguity
